# Theoretical Improvements in Enzyme Efficiency Associated with Noisy Rate Constants and Increased Dissipation

**DOI:** 10.3390/e26020151

**Published:** 2024-02-09

**Authors:** Davor Juretić, Željana Bonačić Lošić

**Affiliations:** 1 Mediterranean Institute for Life Sciences, Šetalište Ivana Meštrovića 45, 21000 Split, Croatia; 2 Faculty of Science, University of Split, Ruđera Boškovića 33, 21000 Split, Croatia; agicz@pmfst.hr

**Keywords:** enzyme efficiency, entropy production, noise, evolution, catalytic cycle

## Abstract

Previous studies have revealed the extraordinarily large catalytic efficiency of some enzymes. High catalytic proficiency is an essential accomplishment of biological evolution. Natural selection led to the increased turnover number, k_cat_, and enzyme efficiency, k_cat_/K_M_, of uni–uni enzymes, which convert a single substrate into a single product. We added or multiplied random noise with chosen rate constants to explore the correlation between dissipation and catalytic efficiency for ten enzymes: beta-galactosidase, glucose isomerase, β-lactamases from three bacterial strains, ketosteroid isomerase, triosephosphate isomerase, and carbonic anhydrase I, II, and T200H. Our results highlight the role of biological evolution in accelerating thermodynamic evolution. The catalytic performance of these enzymes is proportional to overall entropy production—the main parameter from irreversible thermodynamics. That parameter is also proportional to the evolutionary distance of β-lactamases PC1, RTEM, and Lac-1 when natural or artificial evolution produces the optimal or maximal possible catalytic efficiency. De novo enzyme design and attempts to speed up the rate-limiting catalytic steps may profit from the described connection between kinetics and thermodynamics.

## 1. Introduction

There would be no evolution without dissipation. For isothermal conditions, dissipation and entropy production are different names for the same fundamental physical quantity for irreversible thermodynamics which measures the speed of irreversible, far-from-equilibrium processes. In biological research, one can ask about the most basic level at which we can still see a considerable increase in entropy production.

Enzymes are housekeeping cellular macromolecules performing all biosynthetic and moving functions. Enzymes with a uni–uni catalytic mechanism convert a single substrate into a single product (Michaelis–Menten kinetics [1,2]). The assumption is that they have one catalytic site, which only interacts with a specific substrate and converts it into a single specific product. Their enzyme efficiency is the ratio k_cat_/K_M_, where k_cat_ is the catalytic constant and K_M_ is the Michaelis–Menten constant related to the enzyme’s affinity to the substrate. The other name for k_cat_ is the turnover number or cycle completion time. The other name for k_cat_/K_M_ is the specificity or catalytic constant.

Our previous publications (collected in [3,4]) examined how to model enzyme catalytic efficiency increase when the partial entropy production value is maximized in the rate-limiting catalytic steps. The value has the units M^−1^s^−1^ and can be huge for the most efficient enzymes. Thus, it should not be confused with the dimensionless thermodynamic efficiency from physics that is restricted to 0–1 numbers (0 to 100%). Also, k_cat_/K_M_ is not the thermodynamic enzyme efficiency of biological molecular motors and membrane proton pumps, which perform free-energy transduction and are modeled with two or more connected cycles [5,6]. Nevertheless, in the Introduction of [4], there is a section named “Catalytic efficiency increases together with entropy production”. It did not lead to rigorous examination when it did and did not happen.

The metabolic heat production is due to enzymes. Using the microcalorimetry method, Sica et al. found the proportionality between enzyme activity, k_cat_, and observed heat flow [7]. Thermal power was directly proportional to the reaction rate for dihydrofolate reductase (EC 1.5.1.3). Todd and Gomez [8] extended that observation to representative enzymes from each EC classification (a total of 11 different enzymes), assuming the validity of the Michaelis–Menten equation [1]. They found a reasonably good agreement between kinetic parameters k_cat_, K_M_, and k_cat_/K_M_ assayed colorimetrically and with other methods. Riedel et al. [9] confirmed the agreement of the calorimetric and kinetic parameters k_cat_ and K_M_ for catalase, urease, alkaline phosphatase, and triosephosphate isomerase.

There are indications that the first several hundred million years of the Archaean age were enough to develop enzymes catalyzing the same reaction with many orders of magnitude higher activity than the best inorganic catalysts [10,11,12]. Life accelerated spontaneous inorganic evolution billions or quintillions of times [11,13,14,15,16,17]. To gain better insights into how this has been achieved and whether further efficiency improvements are theoretically possible, we explore the connection between the thermodynamic and biological evolution of enzymes in this study. The work will focus on how an irreversible increase in entropy can increase catalytic efficiency.

Surprisingly, inquiries about whether enzyme efficiency has anything to do with total entropy production attracted scant attention in the published literature. Offered answers ranged from generalizations based on the study of two or three points and only one enzyme [18] to the lack of overall correlation between reaction thermodynamics and performance parameters for many enzymes [19]. To some biologists and physicists, it looked evident that biological evolution led to a decrease in entropy, while thermodynamic evolution can lead only to an increase in entropy. Consequently, they could agree with the expectation that biological evolution should strive to produce a minimal amount of entropy. Published contrary conclusions about maximal entropy production during biological evolution [20,21] did not prevail. A reader can find a rich array of literature sources for both viewpoints in a recent book [4].

Michaelis–Menten kinetics [1,2] survived more than 100 years of enzyme catalysis studies [22,23]. It is a good enough reason why a better connection with nonequilibrium thermodynamic parameters should be desirable. The thermodynamic treatment requires the generalization of Michaelis–Menten kinetics so that all catalytic steps are reversible [24]. That does not prevent the highly irreversible nature of some catalytic steps. Thus, we explored the theoretical optimization of generalized Michaelis–Menten-type kinetics when some or all microscopic rate constants exhibit noise arbitrarily far from thermodynamic equilibrium. The variations we introduced are a stepway increase in chosen rate constants, uniform, or Gaussian noise. Remarkably, when equilibrium constants are not altered in the catalytic steps, almost perfect proportionality is revealed between enzyme efficiency and total entropy production.

To avoid generalities, we examined the well-defined short-term evolution of chosen enzymes, their substrates, and products in a system devoid of other biological molecules. The known mechanism of action and all microscopic rate constants calculated or estimated from the experimental data were our main criteria for selecting the enzymes. We set up the initial nonequilibrium state by choosing the out-of-equilibrium substrate and product concentrations. We constructed different software tools to reproduce the measured performance parameters and study possible improvements in our simulations because we wanted to employ deterministic and stochastic models, which complement each other. Former models took less time to produce reproducible results. Stochastic models imitate biological mechanisms that harness molecular noise to create variable beneficial outcomes [25].

Allowing for normal noise in microscopic rate constants has several advantages. Firstly, it is a more realistic description of in vivo biochemical kinetics in a highly noisy cellular microenvironment. Secondly, it considers that experimental data are signals extracted from noise. Thirdly, it allows a comprehensive exploration of rate constant combinations associated with higher enzyme efficiency. The last, but not the least, essential advantages of considering noise are the implications of coupled increases for entropy production and enzyme efficiency during biological evolution.

The proportionality between the biochemical and physical descriptions of the enzyme’s hallmarks (k_cat_/K_M_ and entropy production) does not depend on noise distribution or the programming language used to incorporate noise. However, homeostatic conditions must be assumed to maintain proportionality. These are physiological conditions for in vivo enzyme activity or quasi-steady-state constraints for batch reactor experiments, achieved by continuously removing excess products and adding substrates (the chemiosmotic situation).

In cases when some analytical function is a good fit for efficiency-to-dissipation dependence, its shape is highly dependent on imposed constraints and on the manner of introducing noise in the system. Based on this study, dissipation and catalytic efficiency are correlated through a common dependence on the rate constants in a stable environment.

This work deals with the total entropy production role of very different single-cycle enzymes. The well-known triosephosphate isomerase kinetics was the testing ground for different optimization methods and constraints on rate constant variations. Other explored enzymes also have their own individuality and specific importance. Thus, we first calculated their reference state parameters for the subsequent exploration of how much we can improve their performance. We addressed the question of the influence of model complexity (two, three, or four states) on performance parameters and efficiency–dissipation proportionality. We also explored changes in the partial entropy production of catalytic steps to find those that increased catalytic efficiency. The main text examined only one of three evolutionary-related β-lactamases and a single carbonic anhydrase isoenzyme to save space. We recommend reading the Supplementary Materials to better understand those enzyme sets’ relationships.

## 2. Methods

### 2.1. Selected Enzymes for the Computational Modeling

**Figure 1:**
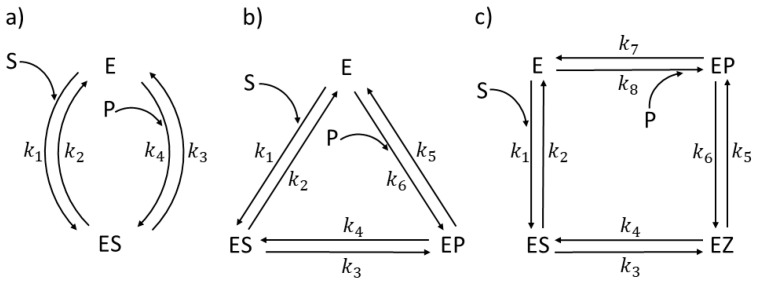
Schemes for the enzyme reactions of the Michaelis–Menten type with (**a**) two, (**b**) three, and (**c**) four states.

We selected the following enzymes with rate constants measured or estimated: *Escherichia coli* β-galactosidase (βG, EC 3.2.1.23) [26] and *Streptomyces murinus* glucose isomerase (GI, EC 5.3.1.5) [27,28] for two functional states (Figure 1a), β-lactamases (EC 3.5.2.6) from three bacterial strains-*Staphylococcus aureus*, *Escherichia coli*, and *Bacillus cereus* enzymes (respectively labeled as PC1, RTEM, and Lac-1) [29,30] for three functional states (Figure 1b), *Commamonas testosteroni* ketosteroid isomerase (KSI, 5.3.3.1) [31], rabbit muscle triosephosphate isomerase (TPI, EC 5.3.1.1) [32], and human carbonic anhydrase I and II (CA I and CA II, EC 4.2.1.1)) [33] for four functional states (Figure 1c). We used the modification of the four-state reversible kinetic scheme for the three CA isoenzymes shown in Figure 13 to include the buffer as the second substrate in the fourth catalytic step. That is the only exception from the strictly uni–uni catalytic mechanism.

### 2.2. Description of Enzyme Kinetics in Terms of Nonequilibrium Thermodynamics

To evaluate enzyme efficiency and its total entropy production in a quasi-steady state and simulated dynamical changes in the concentration of substrates, products, free enzymes, and enzyme complexes with ligands, we used T. Hill’s diagram method [5,6]. Namely, each enzyme can be found in different states, either as free or in complexes, among which possible transitions are shown in Figure 1. The first-order rate constants, *k_i_*, *characterize transitions*, where *i* is odd in the forward direction and even in the backward direction. For the binding transitions with the substrate or product, we use k*_i_* = k_i_*[S] and k*_j_* = k_j_*[P], where k_i_* and k_j_* are the second-rate constants and [S] and [P] are concentrations of the substrate and product. The equilibrium constant K*_i_* in the *i*th catalytic step is defined as the forward-to-backward rate constant ratio K*_i_* = k*_2i_*_−1_/k*_2i_*.

The entropy production density is the sum of force-flux products corresponding to individual transitions (for review, see [34,35]). Total entropy production for an enzyme reaction with a single cycle is given by [30,35,36]

(1) $$\sigma =\frac{JX}{T}$$

where *J* is the steady-state net reaction flux, *X* is the overall steady-state thermodynamic force, and *T* is the absolute temperature assumed to be constant. *J* is the probability current equal to the reaction rate per total enzyme concentration [30,37]. The flux depends on the steady-state occupancy of functional states for an enzyme going through the catalytic cycle and rate constants. The steady-state overall reaction flux corresponds to the flux of an arbitrary enzyme transition *i*→*i* + 1, as all transition fluxes within a single cycle are equal and read [5,6,35]

(2) $$J=J_{i}=k_{2i-1}p_{i}-k_{2i}p_{i+1}$$

where $p_{i}$ is the stationary probability of the *i*th state that can be expressed through the ratio of the sum of the directional diagrams of the state *i* to the sum of the directional diagrams of all states as

(3) $$p_{i}=\sum_{i}/\sum$$

Reaction flux *J* is a function of the forward and backward reaction rate constants. For instance,

(4) $$J=\frac{k_{1}k_{3}-k_{2}k_{4}}{k_{1}+k_{2}{+k}_{3}+k_{4}}$$

for the two-state model shown in Figure 1a,

(5) $$J=\frac{k_{1}k_{3}k_{5}-k_{2}k_{4}k_{6}}{k_{1}\left({k_{3}+k_{4}+k}_{5}\right)+k_{2}k_{4}+k_{2}k_{5}{+k}_{3}k_{5}+k_{6}\left({k_{2}+k_{3}+k}_{4}\right)}$$

for the three-state model shown in Figure 1b and

(6) $$J=\frac{k_{1}k_{3}k_{5}k_{7}-k_{2}k_{4}k_{6}k_{8}}{\Sigma_{1}+\Sigma_{2}+\Sigma_{3}+\Sigma_{4}}$$

(7) $$\Sigma_{1}=k_{2}k_{4}k_{6}+k_{2}k_{4}k_{7}+k_{2}k_{5}k_{7}+k_{3}k_{5}k_{7}\;\Sigma_{2}=k_{1}k_{5}k_{7}+k_{4}k_{6}k_{8}+k_{1}k_{4}k_{6}+k_{1}k_{4}k_{7}\;\Sigma_{3}=k_{1}k_{3}k_{7}+k_{2}k_{6}k_{8}+k_{3}k_{6}k_{8}+k_{1}k_{3}k_{6}\;\Sigma_{4}=k_{2}k_{4}k_{8}+k_{1}k_{3}k_{5}+k_{3}k_{5}k_{7}+k_{2}k_{5}k_{7}$$

for the four-state model shown in Figure 1c.

The thermodynamic force in the cycle that drives the flux equals the sum of forces in each transition

(8) $$X=X_{1}+\ldots +X_{n}$$

where *n* is the number of states and $X_{i}$ is the thermodynamic force of the transition *i*→*i* + 1, which is equal to the difference in chemical potentials

(9) $$X_{i}=\mu_{i}-\mu_{i+1}$$

The chemical potential for the state *i* is given by [5,6]

(10) $$\mu_{i}=G_{i}+RTlnp_{i}$$

where $G_{i}$ is the Gibbs free energy of an enzyme in the state *i* and R is the gas constant. Thus, the thermodynamic force becomes

(11) $$X_{i}=G_{i}-G_{i+1}+RTln\left(\frac{p_{i}}{p_{i+1}}\right)$$

For the transition *i*→*i* + 1, the relation between the difference in the Gibbs free energy and the first-order rate constants is

(12) $$G_{i}-G_{i+1}=RTln\left(\frac{k_{2i-1}}{k_{2i}}\right)$$

giving the thermodynamic force

(13) $$X_{i}=RTln\frac{k_{2i-1}p_{i}}{k_{2i}p_{i+1}}$$

For the two-state model

(14) $$X_{1}=RTln\frac{k_{1}\left(k_{2}+k_{3}\right)}{k_{2}\left(k_{1}+k_{4}\right)}$$

(15) $$X_{2}=RTln\frac{k_{3}\left(k_{1}+k_{4}\right)}{k_{4}\left(k_{2}+k_{3}\right)}.$$

Thus,

(16) $$X=RTln\frac{k_{1}k_{3}}{k_{2}k_{4}}=RTlnK$$

where *K = K*_1_* · K*_2_ is the equilibrium constant. For the three-state model

(17) $$X_{1}=RTln\frac{k_{1}\left(k_{3}k_{5}+k_{2}k_{4}+k_{2}k_{5}\right)}{k_{2}\left(k_{1}k_{4}+k_{1}k_{5}+k_{4}k_{6}\right)}$$

(18) $$X_{2}=RTln\frac{k_{2}\left(k_{1}k_{4}+k_{1}k_{5}+k_{4}k_{6}\right)}{k_{3}\left(k_{1}k_{3}+k_{2}k_{6}+k_{3}k_{6}\right)}$$

(19) $$X_{3}=RTln\frac{k_{3}\left(k_{1}k_{3}+k_{2}k_{6}+k_{3}k_{6}\right)}{k_{1}\left(k_{3}k_{5}+k_{2}k_{4}+k_{2}k_{5}\right)}$$

Then, the overall thermodynamic force is

(20) $$X=RTln\frac{k_{1}k_{3}k_{5}}{k_{2}k_{4}k_{6}}=RTlnK$$

where K = K_1_ · K_2_ · K_3_.

In the case of four states, where

(21) $$X_{i}=RTln\frac{k_{2i-1}\Sigma_{i}}{k_{2i}\Sigma_{i+1}}$$

and $\Sigma =\Sigma_{1}+\Sigma_{2}+\Sigma_{3}+\Sigma_{4}$ with Σ*_i_* given by (7), the overall thermodynamic force becomes

(22) $$X=RTln\frac{k_{1}k_{3}k_{5}k_{7}}{k_{2}k_{4}k_{6}k_{8}}=RTlnK$$

where K = K_1_ · K_2_ · K_3_ · K_4_.

The dissipation is defined as the product of flux and force. At a constant temperature, the absolute temperature T makes the only difference between the entropy production and the dissipation function. Thus, for isothermal conditions, the dissipation function φ and the total entropy production σ have the absolute temperature T as the proportionality factor: φ = T · σ. Entropy production can be expressed in inverse seconds and labeled as P when divided by R: P = σ/R. Similarly, the dissipation function divided by RT is labeled as φ/RT = σ/R = P. In most figures, we use the dissipation term to plot the functional relationship between k_cat_/K_M_ values at the *y*-axis (in M^−1^s^−1^ units) and the dissipation/RT values at the *x*-axis (in s^−1^ units).

Hill’s equations above are valid only for the steady-state kinetics. However, these steady states can be very far from equilibrium. That is the primary advantage over applications of classical irreversible thermodynamics to small departures from thermodynamic equilibrium under which fluxes are linearly proportional to forces. All enzymes examined in this paper exhibit a nonlinear relationship between fluxes and forces. Nonlinearity allows for more efficient power channeling [38]. We also assumed that all studied systems can jump among quasi-steady states in deterministic or stochastic ways. Random encounters among molecules and accidental conformational changes happen in noisy and crowded environments of any living cell. The analytical approach using the FORTRAN computer language is suitable for modeling deterministic changes. FORTRAN source code produced the same output when random numbers were called to simulate the stochastic noise. This does not take into account random encounters among enzymes and small molecules (substrates, products) when we codify Equations (1)–(22). Agent-based modeling better accounts for the need to consider noisy dynamics, random encounters, and variable outcomes while preserving mass conservation for all forms of ligands and different enzyme conformations. It is, however, considerably slower in comparison to deterministic modeling. Therefore, we constructed novel source codes using the FORTRAN and NetLogo languages to perform our simulations. Although the results often agreed, some significant differences justified the application of both modeling techniques.

Further, catalytic constants (*k*_cat_), Michaelis–Menten constants (*K*_M_), and the specificity constant (*k*_cat_/*K*_M_) for all three schemes for the enzyme reactions shown in Figure 1 can be defined (see Section 1 for their meaning). These kinetic parameters have been measured for almost all enzymes, albeit after assuming the single-cycle enzyme turnover or some irreversible catalytic steps. For the two-state reversible model (Figure 1a) [39]

(23) $$k_{cat}\;=k_{3}$$

(24) $$K_{M}\;=[S]\frac{{k_{2}+k}_{3}}{k_{1}}$$

(25) $$\frac{k_{cat}}{K_{M}}=\frac{k_{1}k_{3}}{\left[S\right](k_{2}+k_{3})}$$

for the three-state model [37,40]

(26) $$k_{cat}=\frac{k_{5}}{1+\frac{k_{4}}{k_{3}}+\frac{k_{5}}{k_{3}}}$$

(27) $$K_{M}=[S]\frac{K_{2}\frac{k_{5}}{k_{1}}+\frac{1}{K_{1}}\left(\frac{k_{5}}{k_{4}}+1\right)}{1+K_{2}+\frac{k_{5}}{k_{4}}}$$

(28) $$\frac{k_{cat}}{K_{M}}=\frac{k_{1}k_{3}k_{5}}{\left[S\right](k_{2}k_{4}+k_{2}k_{5}+k_{3}k_{5})}$$

and for the four-state model [41,42]

(29) $$k_{cat}=\frac{k_{3}}{1+\frac{k_{3}}{k_{7}}+\frac{k_{3}}{k_{5}}\left(1+\frac{1}{K_{2}}\right)\left(1+\frac{1}{K_{3}}\frac{k_{5}}{k_{7}}\right)}$$

(30) $$K_{M}=\frac{\left[S\right]}{K_{1}}\frac{1+K_{1}\frac{k_{3}}{k_{1}}+\frac{1}{K_{2}}\frac{k_{3}}{k_{5}}\left(1+\frac{1}{K_{3}}\frac{k_{5}}{k_{7}}\right)}{1+\frac{k_{3}}{k_{7}}+\frac{k_{3}}{k_{5}}\left(1+\frac{1}{K_{2}}\right)\left(1+\frac{1}{K_{3}}\frac{k_{5}}{k_{7}}\right)}$$

(31) $$\frac{k_{cat}}{K_{M}}=\frac{{k_{1}k_{3}k_{5}k}_{7}}{\left[S\right](k_{2}k_{4}k_{6}+k_{2}k_{4}k_{7}+k_{2}k_{5}k_{7}+k_{3}k_{5}k_{7})}$$

We refer the reader to the Supplementary Materials files “Linear Specificity-Dissipation Relationships” and “Flux-Turnover Relationships” for the mathematical understanding of (a) the quasi-proportionality between enzyme efficiency (specificity) and entropy production within the small-change constraints in the equilibrium constants for catalytic steps, and (b) the conditions of the linear-like relationship between the forward catalytic constant (turnover rate) and the net S→P flux.

## 3. Software and Programs Used in This Paper

The same Equations (1)–(33) are employed in the construction of our source codes by using various computer languages. The source codes differ in applied constraints, while the presented figures use the selected parameters from one or more program outputs as we describe in their legends. Using the Box–Muller transform [43], we introduced normal noise into microscopic rate constants. That transform is

(32) $$g_{i}=\sqrt{-2lns_{1}}\cos\left(2\pi s_{2}\right)+shift$$

or

(33) $$g_{i}=\sqrt{-2lns_{1}}\sin\left(2\pi s_{2}\right)+shift$$

For instance, the rate constant *k_i_ = k_i_^exp^g_i_* is noisy when *s_1_* and *s_2_* are random numbers chosen from the unit interval (0, 1) obtained by the standard FORTRAN generator *random_number*, and *k_i_^exp^* is its experimental value. We are the authors of the FORTRAN source codes. Fifteen codes are available for download in the Supplementary Materials (SMs). Each is associated with the main text or Supplementary Materials figures we constructed using relevant software output. For some of our FORTRAN programs, shift = +1 or shift = +2, are used instead of shift = 0 to avoid negative numbers for rate constants.

We also used agent-based modeling [44,45,46,47] to construct source codes for our simulations. Modeling flexibility, inherent dynamics, the ability to model individual behavior, spatial consideration, and the logical entrance of complexity and noise in the system are some advantages of mimicking biological processes with agent-based modeling.

The dynamics can be simulated using the NetLogo language without solving differential equations. NetLogo (http://ccl.northwestern.edu/netlogo/, accessed on 26 January 2024) is a multiagent simulation environment that is simple to use and suitable for modeling the stochastic dynamics of biological processes [48,49,50,51]. Agent-based models are stochastic by nature. For instance, scientists have constructed NetLogo models for the stochastic interaction of an enzyme with its substrates, products, and inhibitors [52,53]. We used the same parent NetLogo source code as the inspiration for all our NetLogo programs. “Enzyme Kinetics” was created by Stieff and Wilensky in 2001: https://ccl.northwestern.edu/netlogo/models/EnzymeKinetics, accessed on 26 January 2024. In our NetLogo source codes (see Supplementary Materials), noise is introduced into selected microscopic rate constants either through random-float values (uniform noise) added to them or by multiplicating them with Gaussian random number values (normal noise, see Equation (32)). Additional noise in the rate constants is due to chance encounters among ligands and [E]_free_ and for transitions between enzyme conformations, which is also specified with several different random-float values.

Furthermore, random changes occur in all computational steps (“ticks”). Ticks can be in chosen time units. Agent-based programming requires dimensionless numbers as the input, but a suitable multiplication factor converts these numbers into micromoles or moles. D.J. is the author of all NetLogo source codes mentioned in the main text and the Supplementary Materials (17 codes). They are available for download in the Supplementary Materials.

For the source codes we developed in the FORTRAN computer language, we verified that, no matter how much noise was introduced, all results and all the data inserted in corresponding figures are exactly reproducible when the same program is repeatedly run for the same number of interstate jumps. When random numbers *s_1_* and *s_2_* were called once, the corresponding Box–Muller transform was identical for all noisy rate constants. Noise was then canceled in ratios of selected rate constants. For instance, the expressions (k_cat_/K_M_)/Dissipation (the slope of the k_cat_/K_M_ dependence on dissipation) from the Supplementary Materials file “Linear Specificity-Dissipation Relationships” contain only the ratios of rate constants. Thus, the slope never changes if random numbers *s_1_* and *s_2_* are called once. It resulted in the perfect proportionality between the catalytic efficiency and entropy production when equilibrium constants for all catalytic steps were fixed.

Noise survived only in expressions containing some of the selected constants that could not be rendered as belonging to such ratios. In other programs, random numbers *s_1_* and *s_2_* were called for each of the selected rate constants, and there was no noise cancelation in their ratios. In some cases, we used the Box–Muller transform to generate noise in selected equilibrium constants using the expression *K_i_ = K_i_^exp^g_i_* (see Equation (32)). The legend of each figure specifies how we used the Box–Muller transform to present the results. When the implicit assumption is that noise does not exist, we used the stepwise increase in the selected rate constant to cover the range, which included the observed k_i_ value.

Our NetLogo source codes extended the simulation “Enzyme Kinetics “(Stieff and Wilensky: https://ccl.northwestern.edu/netlogo/models/EnzymeKinetics, accessed on 26 January 2024) of the traditional Michaelis–Menten model for the reversible E+S↔ES transition and irreversible complex dissociation ES→E+P. We extended it to all reversible transitions. For instance, our four-state schemes used the additional conformational states EZ, EP or EX, and EZ (see Figure 1c and Figure 13). We implemented noise in the microscopic rate constants by using a broader usage of the NetLogo tools as described and regularly updated by Prof. Wilensky’s group [54].

## 4. Triosephosphate Isomerase (TPI): The Favorite Enzyme for Computational Optimization of Michaelis–Menten-Type Kinetics

Triosephosphate isomerase (TPI, EC 5.3.1.1) is an essential enzyme in glycolysis [55,56]. Its central housekeeping role is the very fast catalytic interconversion of dihydroxyacetone phosphate (DHAP) and glyceraldehyde-3-phosphate (GAP). There would be no net yield of ATP from the anaerobic glucose metabolism without TPI forward activity (DHAP→GAP). Of all enzyme-catalyzed reactions, the free-energy profile was first determined for TPI [57]. The seminal works of J. Knowles [32], J. Albery [58], and other authors described TPI as a perfect enzyme, in the sense that it is a perfectly evolved enzyme with catalytic efficiency close to the diffusion limit. In 1984, J. Richard [59] estimated that k_cat_/K_M_ for TPI increased 3 × 10^10^ times compared to the inorganic DHAP to GAP conversion. Enzyme efficiency inside the diffusion limit was confirmed for the wild-type TPI enzymes isolated from many species [60].

As a reversible enzyme working close to the thermodynamic equilibrium, TPI can be easily induced to work in the backward direction (GAP→DHAP). Its central physiological role is maintaining the delicate balance between glycolysis and gluconeogenesis. However, since TPI belongs to the most ancient enzymes [61], its biological evolution involved it in the pentose phosphate pathway, triacylglyceride accumulation, and many other moonlighting functions [62,63]. With such a broad spectrum of activities and functions, it is not surprising that the TPI enzyme has attracted the medical community’s interest. TPI inhibitors are promising as antiprotozoal drugs for the treatment of diseases caused by *Trypanosoma cruzi*, Trypanosoma brucei, Plasmodium falciparum, Giardia lamblia, Leishmania mexicana, Trichomonas vaginalis, and *Entamoeba histolytica* [64]. The upregulation of the TPI gene is common in many cancers [65]. At the same time, TPI deficiency or reduced activity causes the accumulation of DHAP connected to severe diseases, such as hemolytic anemia, recurrent infections, cardiomyopathy, and fatal neuromuscular dysfunction [66].

S. Blacklow asserted [67] that the TPI enzyme “can improve no further as a catalyst”, assuming the constraints of free diffusion and in vivo levels of its substrates. In the meantime, researchers proposed electrostatic screening [68,69], TPI oligomerization [70], elevated temperature for TPI from thermophilic cells [71], and other mechanisms [72] for how TPI catalytic efficiency can be increased above the observed values. Ideally, the mutations or modifications making TPI more resistant to oxidative damage and a more efficient catalyst can help prevent and treat Alzheimer’s disease [72,73].

We stressed in our previous contributions [74,75] that increasing the TPI catalytic turnover and efficiency above observed “perfect” values is theoretically possible when enzyme kinetics is connected to the maximal partial entropy production principle from irreversible thermodynamics [4]. Regarding the simulation of TPI kinetics, we shall attempt to answer the following questions: (a) Does TPI performance change after noise is considered? (b) If it does change, is it possible to find the combination of microscopic rate constants resulting in an at least ten-fold increased performance regarding the k_cat_/K_M_ value calculated from the experimental data? (c) How is the entropy production by TPI related to corresponding enzyme efficiency values? (d) Are any published optimization methods better at finding high forward k_cat_/K_M_ values than different means of noise introduction?

Let us first present the observed values for TPI kinetic parameters [32,74] to easily compare all our simulations with the experimental values (Table 1). Triosephosphate isomerase can be found in four functional states [58]. According to Figure 1c, 1 is the free enzyme (E), 2 is the enzyme–substrate-bound complex (ES), 3 is a transition state intermediate (EZ), and 4 is the enzyme–product-bound complex (EP). The reference steady state [58] is such that the concentration of substrate is [S] = 40 μM and the concentration of product is [P] = 0.064 μM. The values of the kinetic constants k_1_ and k_8_ in Table 1 are obtained, respectively, from the expressions k_1_ = k_1_* · [S] and k_8_ = k_8_* · [P], where the second-order rate constants k_1_* and k_8_* are measured in (Ms)^−1^.

The initial TPI concentration in our simulations ranged from 10 to 50 nM. Mass conservation for all enzyme conformations is always taken into account in all simulations. All NetLogo programs also required the mass conservation of ligands (substrates, products, and their intermediate TPI-bound forms). That requirement was entered into our FORTRAN software as the [S]+[P] = constant condition when we allowed for changes in the concentrations of ligands. The concentration of bound ligands [ES] + [EZ] + [EP] is always much smaller than the [S]_initial_ + [P]_initial_ concentration because bound ligand concentrations cannot exceed the initial low concentration of free TPI enzymes. Thus, the mass conservation of ligands is considered a good approximation in our FORTRAN source codes that examined how different parameters change after changes in the substrate and product concentrations.

### 4.1. Stepwise Changes in Rate Constants

**Figure 2:**
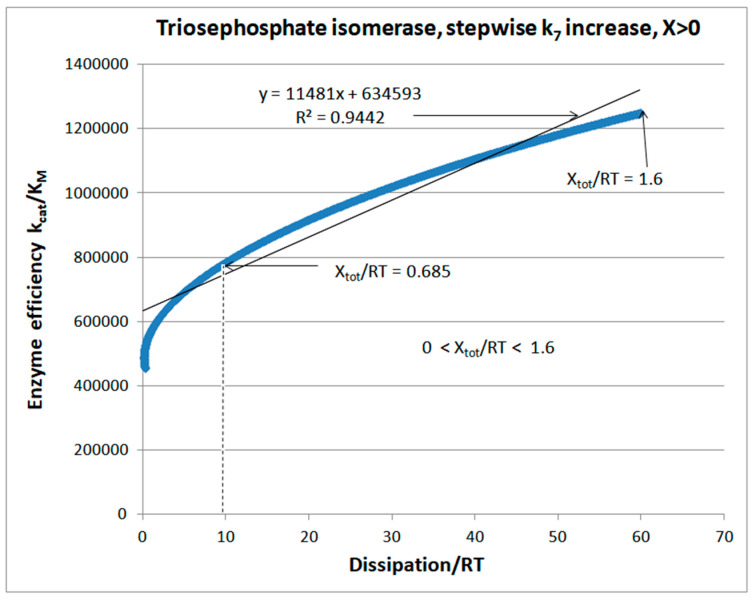
The catalytic efficiency dependence on dissipation after the stepwise increase in the last forward rate constant k_7_ for positive force values. The k_7_ value jumped 10.0 units in each of the 1000 deterministic steps in our source code Simulation-S1-TPI-FORTRAN (see Supplementary Materials), starting with the k_7_ = 10 s^−1^. The K_4_ = k_7_/k_8_ is then calculated from fixed k_8_ and variable k_7_. There are no changes in the other equilibrium constants. Their values follow from Table 1 as K_1_ = k_1_/k_2_, K_2_ = k_3_/k_4_, and K_3_ = k_5_/k_6_. The X_tot_/RT values also go through the stepwise increase. The near-equilibrium force value of 0.685 was kept constant, as in our previous simulation of TPI kinetics [74]. There was no change in the initial concentrations of substrates (40 μM) and products (0.064 μM). The catalytic efficiency dependence on dissipation has a surprisingly good linear fit with R^2^ = 0.9442.

Let us first consider how catalytic efficiency depends on overall entropy production in a deterministic manner when the implicit assumption is that noise does not exist. The FORTRAN program is convenient to use for such a study. Figure 2 illustrates how the TPI efficiency changes after a stepwise increase in the microscopic rate constant k_7_. All other rate constants and the equilibrium constants K_1_, K_2_, and K_3_ are kept at their observed values (see the calculated values of the rate constants and the values for the initial concentrations of substrates and products from Table 1). Since the equilibrium constant K_4_ = k_7_/k_8_ also goes through the stepwise increase, the expected outcome of the first simulation scenario is a regular increase in the chemical affinity or force (expressed as X_tot_/RT values).

Negative force values correspond to negative backward flux (GAP→DHAP) and positive dissipation, while positive force values correspond to positive forward flux (DHAP→GAP) and positive dissipation. Both limits in the force range, negative and positive, are associated with high dissipation. Still, only the positive limit corresponds to the maximal enzyme efficiency value of 1.25 × 10^6^ M^−1^s^−1^ (Figure 2). That result is an encouraging 1.59-fold increase over the observed value of 7.9 × 10^5^ M^−1^s^−1^ (corresponding to X_tot_/RT = 0.685), but not a significant improvement over the 1.13 × 10^6^ M^−1^s^−1^ value we obtained in an earlier optimization of TPI kinetics [74].

From the output of our source code Simulation-S1-TPI-FORTRAN (see Supplementary Materials), selecting only the positive X_tot_/RT values corresponding to forward performance parameters given in Equations (29)–(31) is easy. The resulting efficiency dependence on dissipation is then well correlated (R^2^ = 0.944) with straight-line proportionality (Figure 2). Thus, from zero forward catalytic efficiency and vanishing entropy production in the thermodynamic equilibrium, there must be an obligatory increase in dissipation, which is tightly coupled to the increase in catalytic efficiency. The same source code allows TPI catalysis in the backward direction (GAP→DHAP). Figure S1 illustrates how TPI kinetics and thermodynamics are connected when X_tot_/RT values go through the stepwise increase from negative, at −5.3, to positive, at 1.6. Negative force values correspond to the net backward flux when the performance parameters in Equations (29)–(31) are no longer appropriate.

We did not ask how to achieve the increase in only the chosen kinetic constant k_7_ in practice without any other change. It is unlikely that random or intentional mutations can ever do so. However, fine-tuning microwave irradiation may produce the nonthermal effect of significantly accelerating the product-release catalytic step (see Section 11). It is easier to answer why the simulations presented in Figure 2 and Figure S1 dealt with the k_7_ stepwise increase. As in [74], we assumed that the product-release rate limits TPI catalytic power. In our notation for rate constants (see Figure 1c), k_7_ is the first-order rate constant, determining the product-release rate.

**Figure 3:**
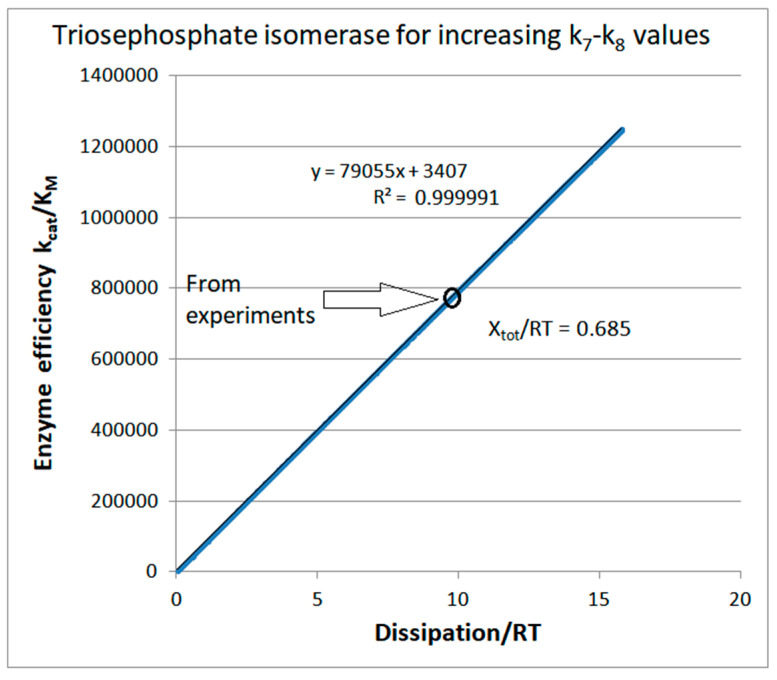
The catalytic efficiency dependence on dissipation for deterministic jumps between steady states, such that increases in the forward rate constant k_7_ (for whatever reason) are constrained by the requirement that the equilibrium constant K_4_ does not change from the observed value K_4_ = 156 [32]. Thus, the backward rate constant k_8_ in the last catalytic step must also go through stepwise increases calculated from the K_4_ = const requirement. Other rate and equilibrium constants remain equal to their initial values (see Table 1). The total force X_tot_/RT remains equal to its initial value of 0.685 through all jumps between the 1000 steady states in our simulation software Simulation-S2-TPI-FORTRAN (see Supplementary Materials). The figure illustrates the perfect proportionality between enzyme efficiency and entropy production when change is not allowed in equilibrium constants for the single-cyclic reversible catalytic steps.

We also explored the stepwise increase of k_7_ and k_8_ when all equilibrium constants and all other rate constants maintain their observed values (see Table 1). Perfect proportionality is obtained between k_cat_/K_M_ and corresponding entropy production values (Figure 3). That proportionality (perfect or less than ideal) is one main result of the present paper. We shall confirm it in the following text for other enzymes and different variation choices for microscopic rate constants whenever the equilibrium constants for all catalytic steps are kept constant. The observed proportionality holds for all our previous publications collected in [3,4] when we used the same constraints to optimize the Michaelis–Menten-type enzyme kinetics.

**Figure 4:**
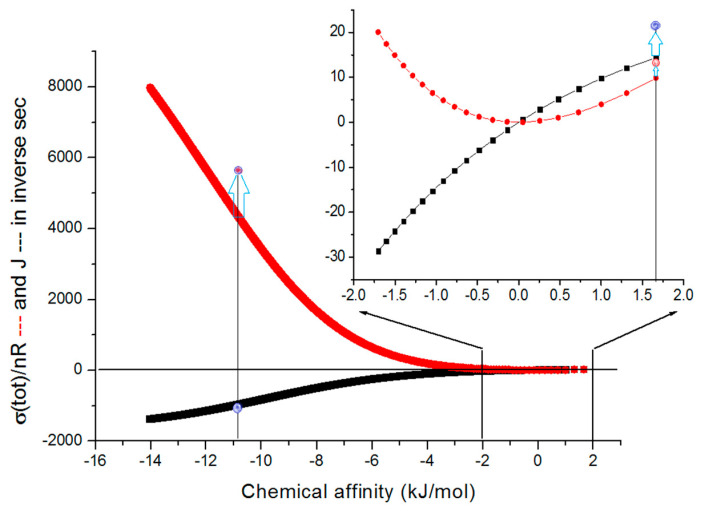
Triosephosphate isomerase thermodynamics and kinetics. Stepwise changes in the substrate and product concentrations are the only causes for the change in the chemical affinity (force) at the *x*-axis and of the entropy production and net flux (both in units of inverse seconds) at the *y*-axis. We assumed that the initial sum of the substrate and product concentrations does not change. Therefore, the decrease in the substrate concentration from its initial concentration of 40 μM is always accompanied by the increase in the product concentration from its initial concentration of 0.064 μM. Other parameters in our simulation software Simulation-S4-TPI-FORTRAN-f90 are the same as in our 2017 paper [74], and we used the symbols from that paper to facilitate the comparison with that and other older simulations. In the figure, we compared our results (the vertical line for the positive force with arrows in the insert) with the simulation results of Šterk et al. [76] (the vertical line for the negative force with arrows in the main figure). We constructed the insert after modifying the Simulation-S4 source code for an indicated narrow range of affinities.

The next task is to answer how thermodynamic and kinetic parameters change for the TPI catalytic cycle when we limit deterministic changes to decreasing substrate and increasing product concentrations. The answer is provided in Figure 4, which illustrates how the net flux and overall dissipation vary with force changes.

The catalytic activity optimizations in the forward direction when the substrate is converted into the product are better connected with the physiological role of the TPI enzyme in glycolysis. We published one example of such optimization in 2017 [74]. It was for the fixed positive force (chemical affinity) corresponding to X_tot_/RT = 0.685 (the vertical line in the insert of Figure 4). The optimization example for the reverse process (product-to-substrate conversion) leads to decreased catalytic efficiency for the forward process. The dissipation and net flux for the reverse process increased by several orders of magnitude when the applied force has a high negative value (the vertical line in the main figure). It is a pathological situation with no connection to TPI’s role in the cellular metabolism. Still, calculated maximum rates for highly negative forces were described as the basic methodology for predicting rate constants and optimizing the TPI kinetics [76,77,78] (see Section 11 for more details).

It all depends on the choice of the optimization procedure. We chose to maximize the partial entropy production in the rate-limiting product-release step (the fourth catalytic step in the forward direction) [4]. We noticed in 2017 [74] how that choice led to the concomitant increase in the optimal net flux (from 14.4 to 20.77 s^−1^), optimal catalytic constant (from 432 to 686 s^−1^), optimal catalytic efficiency (from 7.86 × 10^5^ to 1.13 × 10^6^ M^−1^s^−1^), and optimal overall entropy production (from 9.9 to 14.2 s^−1^). Within the restriction we used (fixed equilibrium constants for each catalytic step at their values calculated from the experimental data), there was a common 30% increase in flux, efficiency, and dissipation. Figure 3 above illustrates how a regular 30% increase follows from the constant slope and perfect proportionality between enzyme kinetic parameters and its overall entropy production.

### 4.2. Computational Optimizations of TPI Catalytic Activity When Noise Is Included

**Figure 5:**
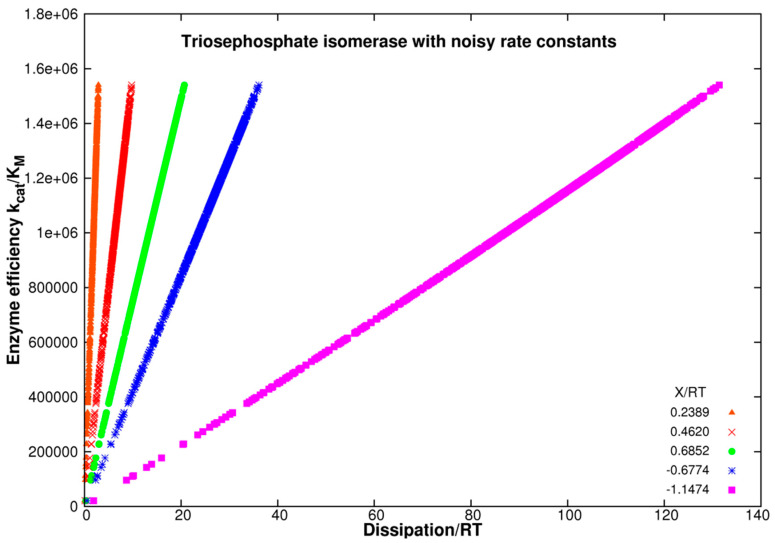
Enzyme efficiency k_cat_/K_M_ as a function of dissipation/RT for triosephosphate isomerase when kinetic constants k_7_ and k_8_ in the last transition vary due to the introduction of Gaussian noise. Representative initial values of rate constant k_8_ are 25 s^−1^, 32 s^−1^, 40 s^−1^, 100 s^−1^, and 160 s^−1^. In each case, we used the K_4_ = const restriction and the experimental data for the kinetic constants shown in Table 1 for transitions between other catalytic steps. However, different k_8_ values resulted in the five different equilibrium constants K_4_ = 160, 125, 100, 40, and 25 to span five force values X_tot_/RT ≡ X/RT from positive to negative (see inserted X/RT values and corresponding symbols). The green points (forming a green line) closely correspond to Figure S2, which we constructed using the Simulation-S3-TPI-FORTRAN software (see Supplementary Materials) for K_4_ = 156.25. We adjusted that software four times to collect the results for the four remaining K_4_ values entered into Figure 5.

Figure 5 illustrates the advantage of using noise when looking for the combination of rate constants corresponding to higher enzyme efficiency. We took the representative initial values of rate constant k_8_ = 25 s^−1^, 32 s^−1^, 40 s^−1^, 100 s^−1^, and 160 s^−1^. Fixed equilibrium constants for each k_7_–k_8_ pair are then K_4_ = 160, 125, 100, 40, and 25, respectively, each calculated using the experimental value k_7_ = 4000 s^−1^. We then introduced random normal noise in forward (k_7_) and backward (k_8_) rate constants for the rate-limiting product-releasing step. Random normal noise was called once in our simulation software Simulation-S4-TPI-FORTRAN (see Supplementary Materials) as the Box–Muller transform (see Section 3, Equation (32)) with the shift +2 to ensure that only positive rate constants k_7_ are the output. There was no need to call that function again for the multiplication with the observed k_8_ value because we kept the no-change requirement for all equilibrium constants K_i_ (i = 1, 2, 3, 4) from our 2017 paper [74] for each K_4_ choice. We used the same restrictions in deriving the partial entropy production theorem [4,30,74].

The best enzyme efficiency values due to introduced noise are approximately the same and about 30% higher from the best result we obtained after exploring maximal partial entropy production for all catalytic steps [74]. For instance, the highest efficiency of 1.6 × 10^6^ M^−1^s^−1^ in Figure S2 for k_7_ = 4000 s^−1^ and K_4_ = 156.25 is associated with the highest total dissipation in the RT units (20.3 s^−1^) due to the perfect proportionality between the main enzyme performance parameter and the main physical parameter in irreversible thermodynamics (Figure S2). Thus, noise introduction does not change the almost perfect straight-line relationship between k_cat_/K_M_ and corresponding entropy production values.

Enzyme efficiency k_cat_/K_M_ as a function of dissipation/RT is shown in Figure 5 for the forces X/RT equal to 0.2389, 0.4620, 0.6852, −0.6774, and −1.1474 corresponding to the equilibrium constants K_4_ = 100, 125, 160, 40, and 25, respectively. We assumed a constant sum of free substrates and free products. It is a good approximation for the mass conservation of ligands only if the initial free enzyme concentration (50 nM) is much smaller than the concentrations of [S]+[P] for all points and all forces. Figure 5 joins the results of five FORTRAN programs that include noise in the last forward catalytic step.

Careful examination of the case X_tot_/RT = −1.1474 reveals a slight curvature in the efficiency as a function of the dissipation (magenta symbols). The slope

$$\frac{k_{cat}/K_{M}}{Dissipation}$$

is not constant because the k_7_ rate constant follows the noise we introduced into k_8_ (see Equation (S3) from the Supplementary Materials file “Linear Specificity-Dissipation Relationships”). The best linear fit slope increases when negative force values approach thermodynamic equilibrium and decreases when positive force increases. Thus, we examined slope changes and goodness of linear fit changes for a wider span of force values ranging from −3 to +4 (Figure S3). For that task, we constructed ten different simulation codes. K_M_ exhibits small changes due to random changes in k_7_ (see Section 2, Equation (30)). Figure S3 shows the output of these programs. It illustrates how the slope and the perfection of the seemingly straight-line proportionality increase with the approach to thermodynamic equilibrium when net force and entropy production vanish.

Next, we studied how the noise introduction affects various computational optimizations for TPI catalysis. In Figure S4, variations of K_1_ and K_4_ were introduced by the multiplication of K_4_ = k_7_/k_8_ (see Table 1) with the normal noise. The fixed force restriction X = X_tot_/RT = 0.684 [74] ensured concomitant variations in K_4_ and K_1_. There was no explicit requirement for maximal entropy production. Still, after going randomly through the 1000 quasi-steady states, our software Simulation-S5-TPI-FORTRAN (see Supplementary Materials) finds that the maximal overall dissipation corresponds to optimal enzyme efficiency (Figure S4). The seven-fold efficiency improvement from 7.86 × 10^5^ to 5.585 × 10^6^ M^−1^s^−1^ follows after a four-fold dissipation increase (see Table 1).

The best combination of the backward rate constants k_2_ and k_8_ (k_2_ = 74 s^−1^ and k_8_ = 2438 s^−1^) resulted in an even higher k_cat_/K_M_ of 8.903 × 10^6^ M^−1^s^−1^. The enzyme working in that state has 11 times higher catalytic activity (the highest point in Figure S4) than the value of 7.86 × 10^5^ M^−1^s^−1^ calculated from the experimental data (Table 1). Required changes in rate constants are two orders of magnitude changes in k_2_ (decrease) and k_8_ (increase). These rate changes describe the inhibition of substrate release from the ES complex and the stimulation of product association with the free enzyme. The corresponding overall dissipation per RT of 21.3 s^−1^ is approximately double the value calculated from the experimental data. Still, the dissipation needed to reach the maximal efficiency state is halved compared to maximal dissipation (Figure S4).

Interestingly, the same dissipation value of 21 to 22 s^−1^ is connected with the two very different catalytic efficiency values of 8.9 × 10^6^ and 1.94 × 10^6^ s^−1^, respectively. Thus, when specific restrictions are imposed, the nonlinear system may be able to jump between two quasi-steady states characterized by high and low efficiency and a minor change in dissipation. How to force the system to live in about a 10-fold higher efficiency state with only a 2-fold higher price in terms of overall dissipation is outside the scope of this paper.

**Figure 6:**
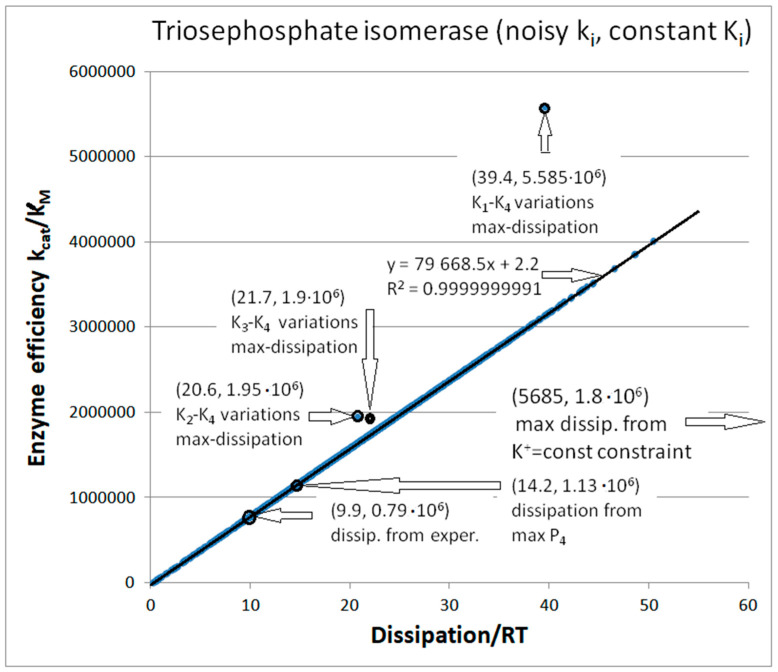
The map of dissipation–efficiency values for different constraints in the case of TPI kinetics. The straight-line efficiency dependence on dissipation follows after the similar restrictions we described in the legend of Figure 5 (green line) and Figure S2. Pairs of (x, y) values with higher (dissipation/RT, k_cat_/K_M_) values from those in Figure S2 resulted because we introduced the same normal noise in all kinetic constants, not just in the k_7_–k_8_ pair. Specifically, we multiplied each of the four forward kinetic constants with the same Box–Muller transform containing two random numbers and a positive shift of +2 (see Section 3), which was called only once in the simulation software. The +2 shift ensured the absence of negative values for some kinetic constants. The corresponding simulation software Simulation-S6-TPI-FORTRAN calculated backward rate constants from the constant K_1_ to K_4_ requirement (their referent values can be calculated from the corresponding rate constant values in Table 1). That requirement ensured, combined with the normal noise introduction in each forward k_i_, that (a) noise was canceled in the ratio of kinetic constants in each catalytic step and (b) all catalytic constants were different in each of the 1000 changes among steady states. See the main text for the meaning of the points (highlighted circles) obtained using different restrictions or optimizations with or without introduced noise.

Optimal efficiency can be obtained for fixed force when other pairs of equilibrium constants are varied by introducing noise. We did not show corresponding efficiency–dissipation dependence because the optimal k_cat_/K_M_ values for the dissipation maximum were considerably lower from the 8.9 × 10^6^ M^−1^s^−1^ value obtained after K_1_–K_4_ variations. We obtained the Figure 6 coordinates (20.6, 1.95 × 10^6^) and (21.7, 1.9 × 10^6^) for the best efficiencies after the K_2_–K_4_ and K_3_–K_4_ variations. An overall conclusion from Figure 6 is that our maximal total entropy production requirement and corresponding restrictions on equilibrium constants for the chosen catalytic steps can produce higher catalytic efficiencies for the fixed force than the maximal selected partial entropy production requirement.

The primary purpose of Figure 6 is to illustrate the relationships among different methods for obtaining higher than referent values for catalytic efficiency (Table 1). That task led to the map of dissipation–efficiency points when the *x*-axis is for dissipation and the *y*-axis is for enzyme efficiency. Perfect efficiency–dissipation proportionality is a straight-line fit to 1000 points after each kinetic constant is multiplied with the normal noise invoked only once in the corresponding FORTRAN program. It is the consequence of assuming fixed values for all equilibrium constants K_i_, meaning that the overall force is also identical for all data points (their referent values can be found in Table 1). Our previous publications did not consider noise and variable equilibrium constants [4,30,74]. The first two highlighted points, (9.9, 0.79 × 10^6^) and (14.2, 1.13 × 10^6^), are centered at the linear fit. They are the dissipation and efficiency values calculated from the experimental data, and the modest improvement achieved after the requirement that partial entropy production P_4_ in the rate-limiting product-release step is maximal [4,74,75].

We discussed above the results after introducing noise in the pairs of equilibrium constants K_1_–K_4_, K_2_–K_4_, and K_3_–K_4_. These are off-line points in Figure 6, respectively: (39.4, 5.585 × 10^6^), (20.6, 1.95 × 10^6^), and (21.7, 1.9 × 10^6^). Thus, Figure 6 clearly shows the advantage of the noisy substrate and product association with the free enzyme (the highest point). After considering many different optimization methods for entropy production (either ours or by other authors), the K_1_–K_4_ variations with a constant force restriction resulted in the best theoretical increase in TPI catalytic efficiency above its observed value [32].

Computational optimizations of TPI kinetics by some other authors [76,77] used substrate and product concentrations similar to each other. It reversed the net flow in the direction of product→substrate due to the negative flux and force, and resulted in higher total entropy production values of several orders of magnitude. For instance, Šterk et al. [76] used the constraint k_1_* · k_3_ · k_5_ · k_7_ = K^+^ = constant equal to the observed value. That constraint also led to maximal total entropy production. Such optimization required that the product of all kinetic constants in the forward direction and all kinetic constants in the backward direction k_2_ · k_4_ · k_6_ · k_8_* remain fixed when other parameters change. As expected, for the backward-directed enzyme turnover, the corresponding optimal efficiency for the forward catalysis of 1.8 × 10^6^ M^−1^s^−1^ (the right-hand arrow pointing outside Figure 6) was substantially smaller than our best results.

**Figure 7:**
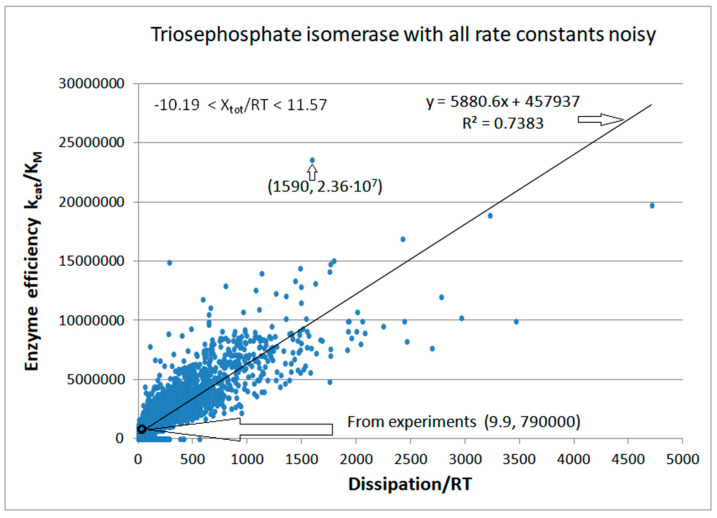
The dependence of catalytic efficiency on dissipation when all rate constants are noisy. Box–Muller transforms g_i_ without shift (see Section 3) were called and multiplied with each of eight rate constants k_i_ (i = 1, 2, …8). Multiplication with g_i_ > 0 introduced normal noise in these constants. For the g_i_ values that did not satisfy the g_i_ > 0 condition, we kept observed k_i_ values (see Table 1). The main loop from our simulation software Simulation-S7-TPI-FORTRAN contained 10,000 steps. After all steps, we examined the kinetic and thermodynamic parameters for maximal values in the catalytic efficiency, overall entropy production, and possible correlation between enzyme efficiency and total dissipation.

We next introduced normal noise in microscopic rate constants with a sole restriction that all rate constants must be positive. When noise is introduced without shift (see Equation (32)) in all rate constants, some k_i_ can vanish or become negative. To avoid such cases, we replaced negative with observed k_i_ values (see Table 1). Figure 7 illustrates that reasonable proportionality exists between efficiency and entropy production when there are no other restrictions on kinetic constants and equilibrium constants for the TPI enzyme. The advantage of calling random numbers eight times (once for each of eight kinetic constants) is an extended range of possible steady states and forces. The highest efficiency state has a 30-fold better efficiency and 160-fold higher dissipation compared to values calculated from experiments. The corresponding force for that state is X_tot_/RT = 6.335.

The basic assumption we used in calculating entropy production values is that each of the 10,000 computational steps probes a new quasi-steady state in which all parameters of interest can be calculated using the T. Hill method [5,6]. We found the maximal efficiency value in the 1078th step. It corresponds to an unusually high information entropy of 1.181 and a low Michaelis-Menten constant of K_M_ = 0.000015. Interestingly, only the kinetic constants k_2_, k_6_, and k_7_ significantly differed from their experimental values, all being much smaller, at 56, 15, and 4 times, respectively. An increase in the k_1_ value (from 400 to 1144 s^−1^) may have resulted from the increased substrate concentration or an increased second-order rate constant for the association between the substrate and enzyme to form the ES complex. There was no change from the experimental values for the kinetic constants k_4_, k_5_, and k_8_.

Enzyme turnover became slightly slower (k_cat_ decreased from 432 to 348 s^−1^), but the division with considerably smaller K_M_ (from 5.5 × 10^−4^ to 1.474 × 10^−5^) ensured a surprisingly high efficiency. As is usually the case, the most illustrative representation is the profile of changes in the equilibrium constants or free-energy changes. The equilibrium constant K_1_ increased about 160 times (from 0.057 to 9.07) and the K_3_ constant increased nearly 15 times (from 0.667 to 9.75). It led to a significant increase in the total equilibrium constant (from 1.98 to 564) despite a decrease in K_2_ (from 0.333 to 0.174) and K_4_ (from 156.25 to 36.59).

There were 3580 points corresponding to the force X = X_tot_/RT ≤ 0. Thus, for 35.8% of sets with random values for kinetic constants, the enzyme can still work in the reverse direction, converting products into substrates. Most k_i_ octuplets simulated the major physiological role of the TPI enzyme in converting DHAP to GAP. The best case of k_cat_/K_M_ = 2.36 × 10^7^ M^−1^s^−1^ is also for the forward-directed net flux. However, we used the same forward catalytic efficiency definition for X > 0 and X ≤ 0. All the experimental data in the literature were extracted for the force X > 0 and flux J > 0 under the conditions when the substrate concentration greatly exceeded the product concentration. The initial concentrations were [S] = 40 μM and [P] = 0.064 μM. Variations in k_1_ and k_8_ allowed changes in the second-order rate constants or in the concentrations. The extreme case was when X = −10.19 was obtained with k_1_ = 3.4 s^−1^ and k_8_ = 27.0 s^−1^. If the change in k_1_ occurred only due to the change in [S], the substrate concentration would decrease almost 120 times. Therefore, although we included the points with negative force and flux in this figure, and other simulations from the literature considered such cases [76,77], there is no experimental or physiological justification for retaining them.

### 4.3. Simulating Dynamics Using an Agent-Based Modeling Approach

Agent-based programming requires dimensionless numbers as the input. However, when these numbers are specified as 40,000 for substrates, 64 for products, and 50 for enzymes (for the TPI kinetics), they correspond to [S]_initial_ = 40 μM, [P]_initial_ = 0.064 μM, and [E]_initial_ = 0.1 μM. The mass conservation of all ligand forms ([S], [P], [ES], [EZ], and [EP]) and all enzyme forms ([E]_free_, [ES], [EZ], and [EP]) is an explicit requirement for each tick in all our NetLogo programs. Thus, [S]_initial_ + [P]_initial_ = [S] + [P] + [ES] + [EZ] + [EP] and [E]_initial_ = [E]_total_ = [E]_free_ + [ES] + [EZ] + [EP], because we left the system to itself and never added ligands or enzymes.

Since the initial product concentration is small (64 nM), each stepwise increase in the product concentration is seen as a jump from one straight-line fit to another in four steps, “a” to “d”. It increased the product concentration to 67 nM. Thus, the proportionality between enzyme efficiency and entropy production (dissipation) remained almost perfect. Maximal efficiency values close to 3 × 10^6^ M^−1^s^−1^ are about four-fold higher than those calculated from experiments. A similar four-fold increase exists for the corresponding dissipation.

When the simulation time was extended to 2137 ticks, the product concentration increased from 64 to 74 nM, while the driving force decreased from X_tot_/RT = 0.68 to X_tot_/RT = 0.54 with the same stepwise slope increase for efficiency–dissipation dependence (Figure S5). The best efficiency value of 3.3 × 10^6^ M^−1^s^−1^ corresponded to the dissipation/RT = 27.2 s^−1^. Free enzyme concentration dropped from 100 to 12 nM for this case of a more extended simulation.

**Figure 8:**
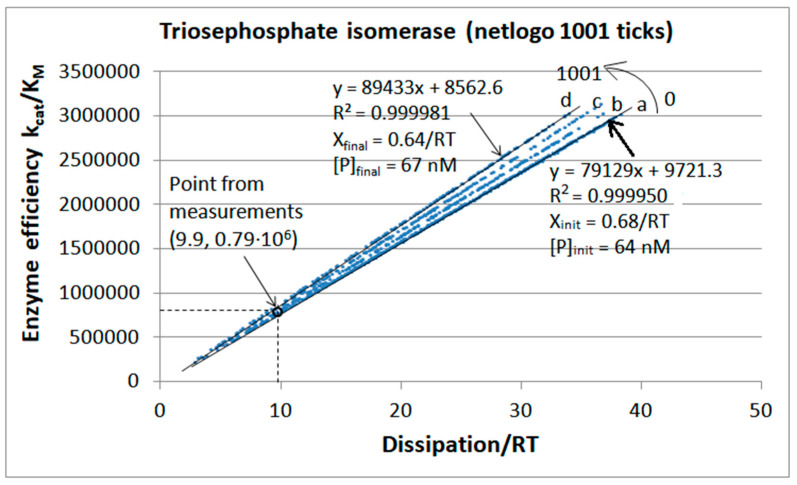
NetLogo simulation of the catalytic efficiency dependence on dissipation for TPI kinetics. The initial enzyme concentration was 100 nM. All other initial values and assumptions were identical to those we used previously [74]. Due to the dynamics inherent to the NetLogo agent-based language, the assumption about unchanged equilibrium constants from that paper could be only partially retained. Noise is introduced through different random-float values, not by Gaussian random number values. Additional noise is due to random encounters among ligands and [enzyme]_free_ and among enzyme conformations [ES]-[EX] and [EX]-[EP] also specified with several different random-float values. The source code for the simulation is available as Simulation-S8-TPI-NetLogo (see Supplementary Materials).

The insight from Figure 8 and Figure S5 would be that maximal catalytic efficiency remains approximately the same during the system relaxation toward thermodynamic equilibrium. The slope of the efficiency–dissipation line keeps increasing toward an infinitely high value at the thermodynamic equilibrium when dissipation vanishes. Also, the perfection of the straight-line approximation for the fit connecting all (x, y) values keeps increasing in discrete jumps (for each unit change in the product concentration) while the chemical affinity decreases. The same time-development rule holds when the equilibrium is spontaneously approached from high positive or negative initial forces (see Figure S3). Better efficiency to dissipation proportionality for positive forces stems from the $k_{cat}/K_{M}$ definition of catalytic efficiency, where both the catalytic constant and the Michaelis–Menten constant are defined for the forward direction $\left[S\right]\to [P]$ (see Section 2 and Equation (S3) from the Supplementary Materials).

For more extended simulations, the concentrations of enzyme conformations ES, EZ, and EP after each step (tick) go through the typical Michaelis–Menten kinetics: slow initial increase, a faster, nearly constant rise, a broad maximum with minor changes, and prolonged decrease. That pattern repeats itself with the ES complex, after some delay with the EZ complex, and finally with the EP complex.

**Figure 9:**
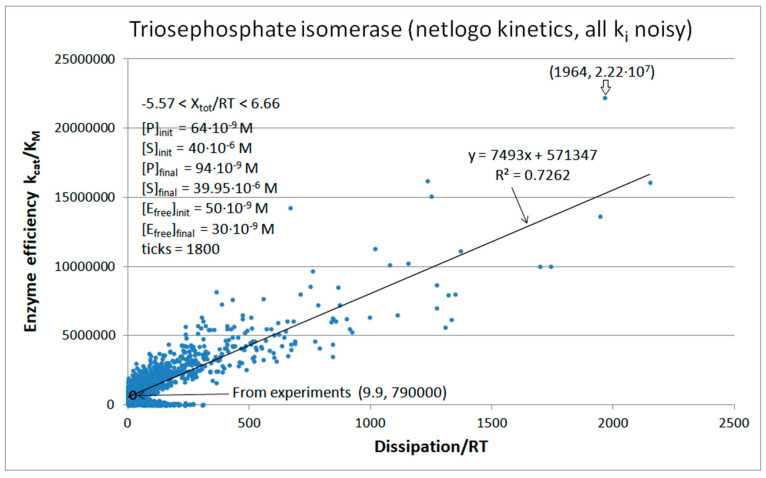
The catalytic efficiency dependence on dissipation when each kinetic constant, k_i_, is independently multiplied with the Gaussian noise function, g_i_ (see Section 3), for the reversible 4-state triosephosphate kinetic scheme. Initial conditions were the same as in Table 1 ([E]_free_ = 50 nM). We constructed the program Simulation-S9-TPI-NetLogo (see Supplementary Materials) to prepare this figure. It was stopped at the 1800th tick. At the 1109th tick, a positive force of 4.26 resulted in the best efficiency of 2.22 × 10^7^ M^−1^s^−1^ and significantly increased k_cat_ = 1085 s^−1^. The final concentrations of enzyme conformations were [E_free_] = 30 nM, [ES] = 6 nM, [EX] = 8 nM, and [EP] = 6 nM.

We next examined if a broader scope search for better enzyme performance is possible when Gaussian noise, g_i_ (see Section 3), is multiplied with each microscopic rate constant, k_i_ (Figure 9). The best catalytic efficiency of k_cat_/K_M_ = 2.22 × 10^7^ M^−1^s^−1^ is indeed better than previous NetLogo simulations and similar to the best result we obtained after a FORTRAN language simulation for the TPI kinetics (Figure 7).

## 5. Ketosteroid Isomerase (KSI) Case: What Is Different When the Operating Range Is Farther from Equilibrium?

P. Talalay discovered, in 1951 [79], the *Pseudomonas testosteroni* bacterium (presently named *Commamonas testosteroni* [80]) from the soil beneath a rosebush on the Berkeley campus. The bacterium could grow in a medium containing testosterone as its only carbon and energy source. That was a clever and brave approach because, at that time, many steroid metabolites were known, but enzymic transformations of steroid hormones and metabolites were yet undiscovered. P. Talalay and his collaborators purified highly active small bacterial enzyme ketosteroid isomerase from that bacterium and reported their findings from 1955 onward [79]. The alternative name for the KSI enzyme is 3-oxo-Δ^5^-steroid isomerase (EC:5.3.3.1).

A. Radzicka and R. Wolfenden reported typical high values for the catalytic constant, catalytic efficiency, and catalytic proficiency of KSI as, respectively, 6.6 × 10^4^ s^−1^, 3.0 × 10^8^ M^−1^s^−1^, and 1.8 × 10^15^ M^−1^ [13]. Catalytic proficiency is the catalytic efficiency rate enhancement (k_cat_/K_M_)/k_uncat_ when a nonenzymatic reaction rate constant k_uncat_ can be found for a corresponding spontaneous chemical reaction without the enzyme (1.7 × 10^−7^ s^−1^ in our case). Thus, KSI is one of the fastest enzymes with extraordinary catalytic power. The formation of essential steroid hormones would take months to millions of years without enzymes such as KSI [81]. The equilibrium constant K_eq_ = 2400 [82] corresponds to far-from-equilibrium conditions, high positive force, and the preference for the forward isomerization rate of 5-androstene-3,17-dione (a substrate for KSI) to its conjugate isomer 4-androstene-3,17-dione. Elucidating how the KSI reaction mechanism is connected to structure, kinetics, electrostatics, and thermodynamics was a challenging but worthy task in the last 50 years [83,84,85,86]. Hopefully, the rational design of KSI enzymes with augmented catalytic efficiency will benefit green chemistry goals for the pharmaceutical industry in manufacturing specialized steroid chemicals [87].

Mammalian steroid isomerases have multifunctional activity and a more complex structure than bacterial KSI enzymes [88]. Although crucial in all mammals, their structure–function connection has not been as extensively examined as in the case of the model enzyme KSI from bacteria. Thus, we shall use the best-predicted KSI rate constants for bacterial KSI [31] that agree well with those reported earlier [82,89].

**Figure 10:**
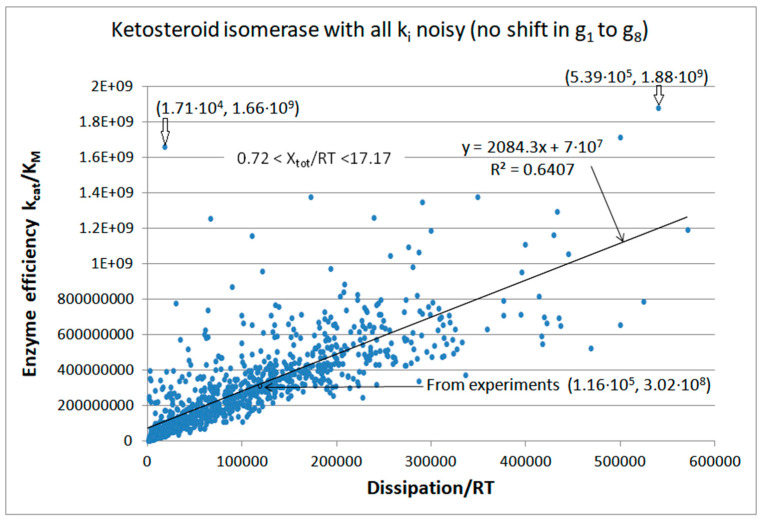
The catalytic efficiency dependence on dissipation when all rate constants are noisy for the ketosteroid isomerase kinetics. A spectrum of quasi-steady states with different k_i_ octuplets resulted after the multiplication of each observed k_i_ (see Table 2) with a separately called normal noise g_i_. There was no shift in eight Box–Muller transforms with the cosine function (see Section 3, Equation (32)). The if–else condition in our Simulation-S10-KSI-FORTRAN software code ensured that negative or zero k_i_ values were replaced with their experimental values. The program went through the 1000 steps, requiring the overall force X to be positive in each step.

Our first task was a broad exploration of possible system states when noise is introduced into each of the eight rate constants for the four-state kinetic scheme (Figure 10). Our FORTRAN simulation kept the concentrations of substrates and products fixed at their initial values (Table 2, last column: [S] = 10^−4^ M, [P] = 5 × 10^−5^ M). Nevertheless, due to random changes in all rate constants, the force changed in the range 0.72 < X_tot_/RT < 17.17. The best efficiency value required a 4.6 times higher dissipation. However, the third best efficiency value from the (1.71 × 10^4^, 1.66 × 10^9^) point reveals that 5-fold higher efficiency can be achieved when the corresponding entropy production is almost 10-fold smaller than their experimental values. That is a rare case when the choice of rate constants results in high catalytic activity despite the low dissipation for the KSI enzyme.

As for the case of triosephosphate isomerase, perfect efficiency–dissipation proportionality followed after the no-change requirement in the equilibrium constants for all catalytic steps. When noise is called only once, a nearly perfect linear fit survives for efficiency–dissipation dependence, no matter how many rate constants, k_i_, are multiplied with the normal noise function. The consequence of fixed equilibrium constants, K_i_, is a constant overall force, too.

**Figure 11:**
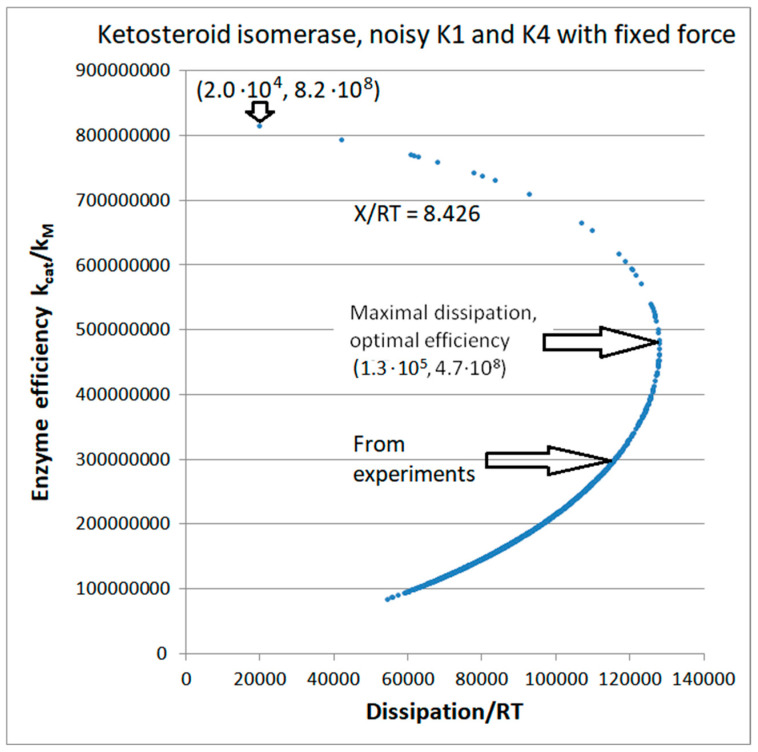
Maximal overall entropy production and associated optimal enzyme efficiency follow from the X_tot_/RT = 8.426 requirement (see Table 2) in the presence of the noisy association–dissociation of the enzyme with the substrate or product. Our Simulation-S11-KSI-FORTRAN software randomly changed equilibrium constants K_1_ and K_4_ while going through 1000 steps.

Regular dependence of enzyme efficiency on overall dissipation follows when noise is introduced only into one or two kinetic constants without fixed K_i_ requirements (Figure 11 and Figure S6). However, that dependence is very different if the overall force X_tot_/RT is allowed to vary too (Figure S6), and when overall force is kept at the constant initial value X_tot_/RT = 8.426 (Figure 11, see Table 2). Figure 11 confirms the observation from Figure S4 that the maximum in overall entropy production exists when variations in K_1_ and K_4_ equilibrium constants are introduced and the fixed overall force is maintained in all simulation steps. Total entropy production is maximal in point (1.3 × 10^5^, 4.7 × 10^8^) (Figure 11). The corresponding optimal efficiency is about 50% higher than the observed value 3.02 × 10^8^ M^−1^s^−1^. Still, the point with the highest efficiency (1.96 × 10^4^, 8.15 × 10^8^) corresponds to a dissipation 5.9 times smaller than the value 1.16 × 10^5^ s^−1^ calculated from the experimental data (see Table 2). That is another rare case when randomly chosen equilibrium constants within an imposed restriction (constant overall force) resulted in a high catalytic efficiency despite the low overall dissipation for the enzyme.

**Figure 12:**
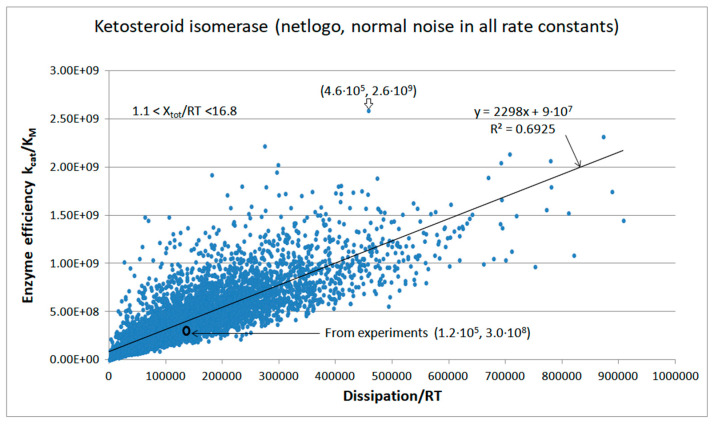
NetLogo simulation of KSI kinetics when the Box–Muller transform without shift is invoked eight times to multiply the eight rate constants k_i_,.This extensive search yielded the highest catalytic efficiency value at the 3911th tick. It was k_cat_/K_M_ = 2.59 × 10^9^ M^−1^s^−1^; thus, it was well inside the diffusion limit range from 10^8^ to 10^10^ M^−1^s^−1^. The corresponding overall force was X_tot_/RT = 12.6. We developed the Simulation-S13-KSI-NetLogo software code (see Supplementary Materials) to construct Figure 12.

Agent-based modeling extended and confirmed the simulation results for KSI kinetics (Figure 12). Typical Michaelis–Menten kinetics for concentration changes, which we described for the NetLogo simulation of TPI kinetics, is also seen for KSI kinetics. Initial concentrations were [E]_free_ = 5 μM, [S]_free_ = 100 μM, and [P]_free_ = 50 μM. Final concentrations at the 6977th tick were [E]_free_ = 4 μM, [S]_free_ = 95 μM, [P]_free_ = 54 μM, [ES] = 0.3 μM, [EX] = 0.4 μM, and [EP] = 0.3 μM. The mass conservation conditions [E_tot_] = [E_free_] + [ES] + [EX] + [EP] and [ligands] = [S]_free_ + [P]_free_ + [ES] + [EX] + [EP] were satisfied through all time jumps (ticks). Freedom to independently change rate constants in each transition and each tick enabled the exploration of a wide range for overall force (1.1 < X_tot_/RT < 16.8), catalytic efficiency, and overall dissipation. The best pair of dissipation–efficiency values (4.6 × 10^5^, 2.6 × 10^9^) corresponded to an approximately 4-fold higher dissipation and almost 10-fold higher efficiency in comparison with the values calculated from observed data (1.2 × 10^5^, 3.0 × 10^8^).

## 6. CA I, CA II, and CA II-T200H (Also Four-State Enzymes)

Carbon dioxide conversion into biomass is essential for the survival and spreading of life in all terrestrial environments. Carbon sequestration is also crucial for the survival of our carbon dioxide-producing civilization, which is unfortunately addicted to fossil fuel burning and breaking all life-supporting balances the biosphere has developed through eons. Nature developed multiple means and different organic structures for the fast conversion of carbon dioxide to bicarbonate—the first step toward carbon fixation. Carbonic anhydrases (CAs) are universal enzymes responsible for that process in all three life domains: Bacteria, Archaea, and Eukarya [90]. With rare exceptions [91], CAs are metalloenzymes containing a metal ion (usually zinc) in their central active-site cavity. From their discovery in red blood cells in 1932, the scientific interest in CAs continued to grow, as seen from the abundance of more than 900 solved CA structures deposited in the Protein Data Bank [92].

The spontaneous reaction of CO_2_ with water can produce bicarbonate ${HCO}_{3}^{-}+H^{+}$, but that reaction is too slow to support respiration [93,94] and other biological processes catalyzed by different CAs. Eight unrelated families of carbonic anhydrase (CA) enzymes represent different ways nature performed the feat of fast catalytic interconversion between carbon dioxide and carbonic oxide [95], reaching the catalytic turnover of 1 μs^−1^ or even higher [96]. There is little or no sequence homology among the CA families α, β, γ, δ, ζ, η, θ, and ι [91,97]. Molecular biologists concluded that convergent biological evolution performed the spectacular function-enhancing feat at least seven times, because different CAs evolved to perform an identical function [95,98,99].

Mammals possess 16 different CA isoenzymes from the alpha-class family [100]. All are metalloenzymes, with the Zn II hydride located at the enzyme center anchored by three histidines. CA isoforms are involved in a variety of physiological functions. Human CA isoforms are well-recognized drug targets for designing isoenzyme-specific inhibitors [101,102] to help fight glaucoma, epilepsy, obesity, cancer, and other diseases. Also, human CA II is one of the most efficient known enzymes. Its calculated catalytic efficiency from experimental data is 1.5 × 10^8^ M^−1^s^−1^ [102]. Earlier efficiency calculations also positioned CA among “perfect” enzymes working close to the diffusion limit [15,60].

The genetic defects of specific CA isoforms can cause osteopetrosis, cerebral calcifications, retinal problems, hyperammonemia, hyperchlorhidrosis, and neurodegenerative and other metabolic diseases [103], which is a good enough reason to look for CA activators [100] or other means for increasing the activity of these isoforms. Memory enhancement can be achieved through CA activation [104]. It opens the possibility for the targeted improvement of brain CA performance to enhance cognition and slow the aging process [100,105]. Some CA mutants can accelerate proton transfer, the rate-limiting step for CA turnover [96]. Another reason for increasing CA activity is the urgent need for the green ways of industrial CO_2_ sequestration [106], which we mentioned above.

Krishnamurthy et al. [94] (Table 1 from [92]) compared all known CA enzymes for their $k_{cat}^{{CO}_{2}}$ and $k_{cat}/K_{M}^{{CO}_{2}}$ values for the catalytic hydration of CO_2_ and the dehydration of bicarbonate:

$${CO}_{2}+H_{2}O\;↔HCO_{3}^{-}+H^{+}$$

That is the first half-reaction. The carbon dioxide ${CO}_{2}$ is the substrate, while the bicarbonate ion $HCO_{3}^{-}$ is the product of the CA catalytic activity in the forward direction. The buffer is the second substrate in the two-substrate ping-pong reaction, which recovers free enzymes.

**Figure 13:**
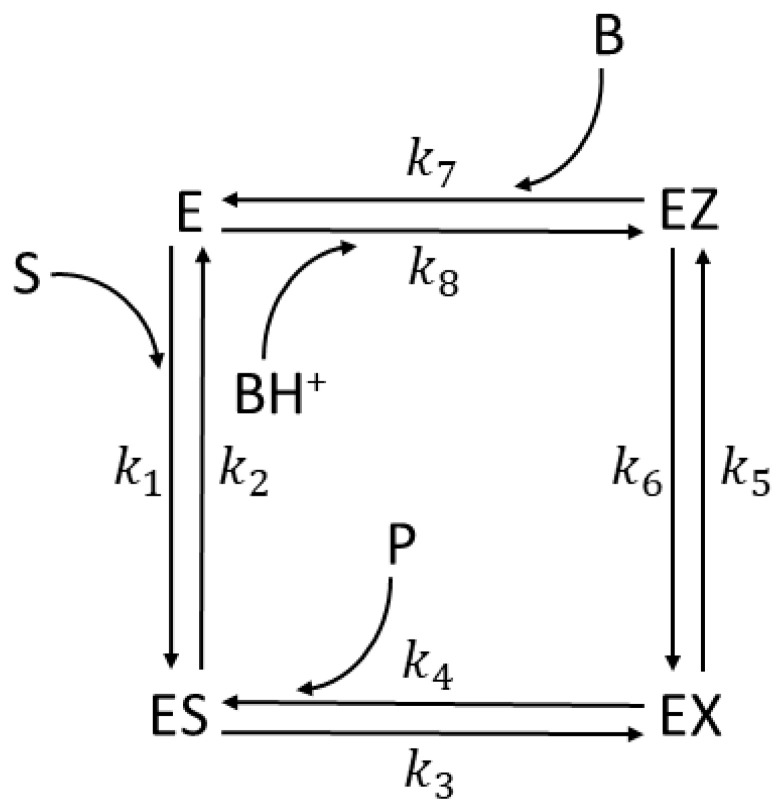
The four-state reversible kinetic scheme for three CA isoenzymes. Each CA converts the ${CO}_{2}$ substrate (S) into the $HCO_{3}^{-}$ product (P) in the second catalytic step. The remaining two catalytic steps in the forward direction serve to recover free enzymes with the help of the buffer (B). Including a buffer in both transitions of the last catalytic step reflects the substantial difference in the performance of all CAs for different buffers [33]. The buffer was 50 mM dimethylimidazole/H_2_SO_4_ (pH = 7.24).

This subsection deals with the theoretical possibilities for catalytic efficiency improvements of human CAs I, II, and the T200H variant of CA II with His200 replacing Thr200 [33]. There may be better models than the four-state kinetic model for reversible Michaelis–Menten-type kinetics (Figure 13). Still, it is based on the publication [33] that contains all microscopic rate constants needed to calculate and compare the enzyme’s performance with associated dissipation. Referent (initial) state values can be found in Table 3.

The simulation of noisy CA I kinetics did not change any of the initial concentrations (Table 3), and it still found in the 246th step a dissipation–efficiency point (5.14 × 10^5^, 1.12 × 10^8^) with 4.5-times higher catalytic efficiency from the calculated value based on the observed kinetic data (Figure S7). The corresponding overall force was positive (X_tot_/RT = 5.0) and closer to the upper end of the force range (X_tot_/RT = 8.3). However, the substantial efficiency increase (4.5-fold) was “paid for” with the 18 times higher overall dissipation. Closer inspection of the performance parameters from the 246th computational step (concerning observed initial values) revealed a 6.3-fold increase in the turnover number and a 2.8-fold increase in the overall force as the main reason for the improved efficiency.

The agent-based simulation of noisy CA I kinetics (Figure S8) slightly changed the initial substrate and product concentrations (Table 3). The 6-fold efficiency increase point, which we found halfway through the simulation, was “paid-for” with the 22-fold dissipation increase. That quasi-steady state corresponded to a nearly three-fold increase in the k_cat_ and overall force.

**Figure 14:**
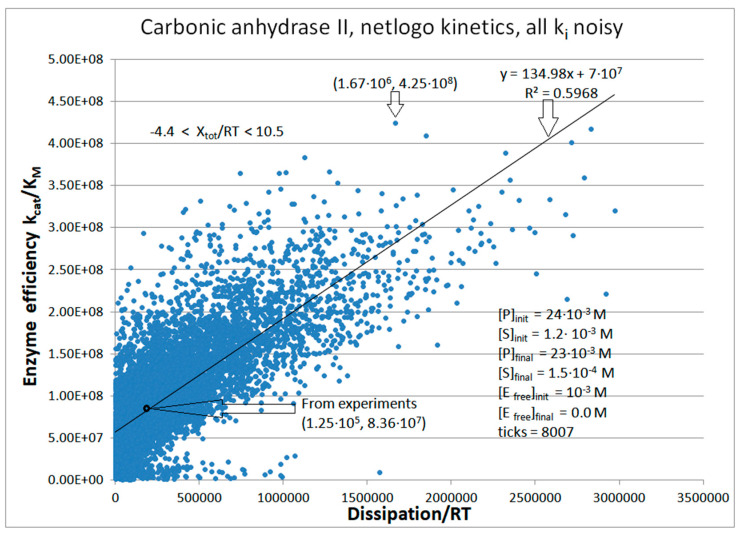
The NetLogo simulation of the relationship between catalytic efficiency and overall dissipation for the carbonic anhydrase II when each of the eight rate constants, k_i_, are multiplied with the independently introduced normal noise function, gi, without shift (see Section 3, Equation (32)). The best efficiency value of 4.25 × 10^8^ M^−1^s^−1^ from the 288th tick is for X_tot_/RT = 4.76. There was no apparent force decrease with the time passage (ticks). From the initial 100 μM free enzyme concentration, the conversion during 8007 ticks ended up with less than 1 μM free enzyme concentration, with [ES] = 1 μM, [EX] = 50 μM, and [EZ] = 49 μM. The Michaels–Menten time-dependence pattern was the same as that seen for CA I. We used our Simulation-S16-CAII-NetLogo software (see Supplementary Materials) to construct Figure 14.

**Figure 15:**
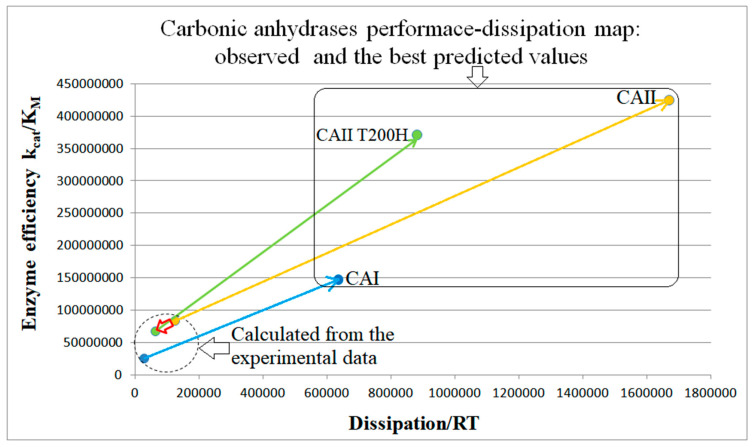
The observed [33] and the best-predicted values for the performance of carbonic anhydrases CA I, CA II, and the T200H mutant of CA II. We collected the NetLogo simulation results from Figures S8 and S9 and Figure 14 to easily compare the enzyme efficiency and overall dissipation calculated from the observed rate constants (dashed circle) and the best-simulated values when the noise was introduced in all k_i_ (rounded rectangle). The arrows connect such points for different isoenzymes (blue for CA I, orange for CA II, and green for the T200H mutant). The small red arrow shows the performance and dissipation decrease for the Thr200→His substitution mutant of carbonic anhydrase II. In contrast, the green arrow indicates the possibility of improving its performance above the observed value for CA II. The potential to enhance the CA II performance (yellow arrow) appears much higher than the CA I (blue arrow).

Human red cell isoenzyme CA II is superior to CA I when their catalytic efficiencies are compared [33] (see Table 3). Thus, simulations for CA II kinetics will have the advantage of starting from a better initial state. Here, we show only the NetLogo simulation (Figure 14). The CA II mutant T200H, constructed by Behravan et al. [33], was an attempt to find the single amino acid substitution that would lead toward the catalytic parameters of CA I. The NetLogo simulation (Figure S9) indicates the evolutionary potential for improving the performance of CA-T200H as being indeed between CA I and CA II, but closer to CA II (Figure 15).

We next used the same constraint of unchanged equilibrium constants in all catalytic steps as for the TPI and KSI enzymes. When identical noise is introduced in all rate constants, the straight-line relationship (perfect proportionality) is revealed between the enzyme efficiency and total dissipation for carbonic anhydrases CA I, CA II, and the T200H mutant of CA II. The correlation between efficiency and dissipation ranged from R^2^ = 0.35 (for T200H mutant) to R^2^ = 0.60 (for CA II) in our NetLogo simulations (Figures S8 and S9 and Figure 14). In Figure 15, we constructed the performance–dissipation map for CA isoenzymes. Values calculated from observed rate constants [33] are confined near the origin of that figure, while the best-simulated values are expanded in the order CA II > CA II T200H > CA I. Improved catalytic efficiency is associated with increased dissipation in the same order.

## 7. Evolutionary Related β-Lactamases

This section extends our earlier studies [3,30], when we examined the evolutionary relationship among bacterial β-lactamases, their kinetic performance parameters, and entropy production. The evolution of β-lactamases, as an example of adaptation in bacteria, is not just of academic interest. Diverse classes of β-lactamases inactivate antibiotics (for instance, ampicillin and cephalosporins) by performing the hydrolysis of their beta-lactam bridge [107]. The rapid global spread of beta-lactamase-mediated bacterial resistance in hospitals has become a severe challenge in treating bacterial infections [108].

We used here the same set of microscopic rate constants for *S. aureus*, *E-coli*, and* B. cereus* enzymes (respectively labeled as PC1, RTEM, and Lac-1) determined during the 1980s [29] together with our estimate for missing backward rate constants [30] that were needed for the calculation of nonequilibrium steady state quantities in the reversible three-state Michaelis–Menten kinetic scheme (Figure 1b). The natural evolution of β-lactamases happened millions of years before the widespread use of penicillin-based antibiotics (β-lactam antibiotics) could accelerate it in the wild-type bacterial species studied during the 1980s [109]. It probably developed as a defense from naturally occurring beta-lactam antibiotics produced by some fungi and bacteria [110]. Thus, evolutionary distances based on β-lactamase sequences determined in the 1970s [3,4,111] should be suitable to study possible connections to the total entropy production as the most crucial quantity in nonequilibrium thermodynamics.

How appropriate is the “perfect” enzyme name for the three-state scheme with some rate constants observed or calculated as representing fast transitions in the case of β-lactamases [29,112]? That general claim about β-lactamases as almost perfect enzymes has been supported for Lac-1 but not for RTEM and PC1 enzymes [113]. Perfect enzymes supplied with their best substrate should be able to operate close to or inside the range 10^8^–10^10^ M^−1^s^−1^ predicted for diffusion-limited enzyme reactions [67]. Collected k_cat_/K_M_ values for the hydrolysis of some characteristic β-lactams by various class A β-lactamases [114] are considerably smaller from the lower end of the diffusion limit despite the “close to the diffusion limit, i.e., 10^8^ M^−1^s^−1^” assertion by these authors. However, the latent potential for these lactamases to evolve further toward higher turnover numbers and catalytic efficiency exists when thermodynamic principles are considered together with kinetic restrictions [3,30].

We used Gaussian noise to explore the combinations of microscopic rate constants and associated dissipation, leading to substantially improved catalytic activity for the PC1, RTEM, and Lac-1 β-lactamases. We also wanted to answer whether efficiency–dissipation proportionality exists for the three-state kinetic scheme named the Haldane reversible three-step model (Figure 1b) [115,116]. The serendipitous discovery from this subsection is that a linear-like relationship survives between the total entropy production increase and evolutionary distance increase (from a putative common ancestor) even after dissipation is calculated for the maximal catalytic efficiency points reached after the noise introduction.

### 7.1. PC1 β-Lactamase

When we maintain the same restrictions of unchanged initial values for the equilibrium constants (except for the changes in the substrate and product concentrations), identical normal noise introduction in all forward kinetic constants leads to only slight changes in the nearly perfect proportionality between catalytic efficiency and dissipation (Figure S10). Besides noise, an additional reason for changes in k_1_ and k_6_ is a decrease in the free substrate concentration and an increase in the free product concentration during the enzyme cycling scheme E+S ↔ ES ↔ EP ↔ E+P. It produces a slight decrease in the first equilibrium constant K_1_ = k_1_* · [S]/k_2_ and an increase in the third equilibrium constant K_3_ = k_5_/(k_6_* · [P]. Increased product concentration is the main reason for the gradual force decrease, from the initial X_tot_/RT = 11.4 to final X_tot_/RT = 10.8, after 5168 ticks of the NetLogo simulation. At the 381st NetLogo simulation tick, we found the best efficiency value of k_cat_/K_M_ = 4.18 × 10^7^ M^−1^s^−1^, which corresponded to the forward rate constants k_1_ = 1.35 × 10^5^ s^−1^, k_3_ = 717 s^−1^, k_5_ = 398 s^−1^, the catalytic constant k_cat_ = 252 s^−1^, and the dissipation/RT = 2823.6 s^−1^. That is the same 4.1-fold increase for all these parameters concerning their observed values (see Table 4).

The next goal is to look for limits to the evolvability of PC1 β-lactamase subject to the variable rate constants k_1_ and k_2_ in the first catalytic step (association–dissociation between the free substrate and free enzyme: E+S↔ES). Figure S11 illustrates how the multiplication of k_1_ and k_2_ with the, respectively, Box–Muller normal noise functions named g_1_ and g_2_ can find a quasi-steady state with 6.5 times higher catalytic efficiency and merely 1.2 times higher dissipation in comparison with those values calculated from experiments (Table 4). That is a significantly better result than all previous optimizations [30] based on the requirement of maximal partial entropy production in proton transfer catalytic steps 2 (ES↔EP) and 3 (EP↔E+P). For instance, joint optimizations of both catalytic steps for maximal transitional entropy production in these steps find about two-fold higher efficiency, which is “paid for” by the 183 times higher dissipation. The maximum total entropy production requirement combined with the obligatory K^+^ = k_1_ · k_3_ · k_5_ = const constraint leads to 333 times lower catalytic efficiency (Figure S11, and [30]).

No further gain in enzyme efficiency follows after normal noise is independently introduced in four or all six rate constants. The maximal k_cat_/K_M_ ranged from 5.9 × 10^7^ to 6.2 × 10^7^ M^−1^s^−1^. Thus, we shall keep the best result from Figure S11 (6.5 × 10^7^ M^−1^s^−1^) to compare the evolutionary potential with other enzymes.

### 7.2. RTEM β-Lactamase

**Figure 16:**
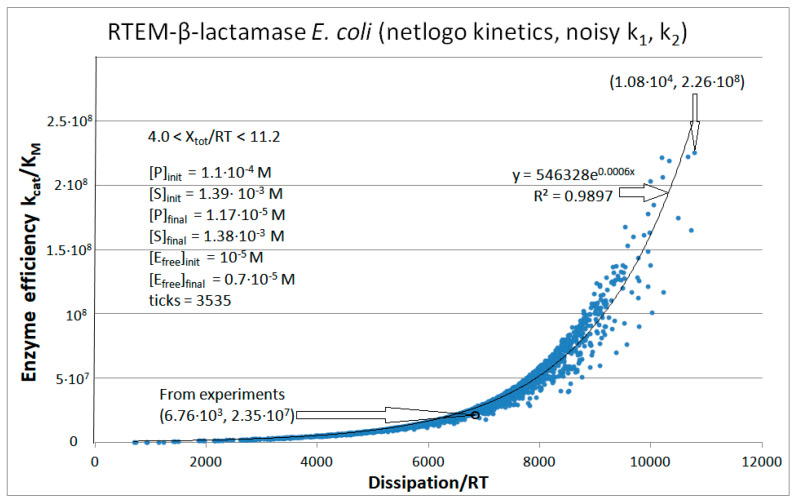
The catalytic efficiency dependence on dissipation when noise is introduced twice in the NetLogo simulation for RTEM β-lactamase kinetics—in the forward rate constant k_1_ and the backward rate constant k_2_. The best catalytic efficiency from this NetLogo simulation (2.26 × 10^8^ M^−1^s^−1^) corresponds to the highest dissipation/RT: 1.08 × 10^4^ s^−1^. Optimal k_1_ increased 2.4-fold, while optimal k_2_ decreased 14-fold, significantly increasing the irreversibility of the substrate interaction with the enzyme. The best-case efficiency is associated with the X_tot_/RT = 11.18 for the 3163rd tick. We used our Simulation-S21-RTEM-NetLogo software (see Supplementary Materials) to construct Figure 16.

Figure S12 and Figure 16 for the NetLogo simulations of the RTEM β-lactamase kinetics are analogs to Figures S10 and S11 for PC1 β-lactamase kinetics. Since RTEM β-lactamase is the evolutionarily more advanced enzyme [3,4,30], the simulations had a head start and ended with higher values for the best catalytic efficiency. The k_cat_/K_M_ = 9.02 × 10^7^ M^−1^s^−1^ point from Figure S12 also corresponds to the maximal dissipation, to the highest values for the forward rate constants, and to the only slightly lower X_tot_/RT = 7.71 compared to the initial value X_tot_/RT = 7.74. That followed from an early 572nd tick when all performance parameters increased about 3.8 times from their initial values (see Table 4).

As for the NetLogo simulation of the PC1 β-lactamase kinetics, variations in the kinetic constants k_1_ and k_2_ resulted in the exponential dependence of the catalytic efficiency on the overall dissipation (Figure 16). It is essential to introduce twice the normal noise, once in the forward direction and once in the backward direction (see Section 3). The beneficial consequence is the possibility of separating the enzyme efficiency from the dissipation increase in favor of a former quantity. The best catalytic efficiency is already well inside the diffusion-limited range.

### 7.3. Lac-1 β-Lactamase

For the case of the Lac-1 β-lactamase kinetics, we explored several options for the independent noise introduction in each kinetic constant from the chosen pairs (Figures S13–S15). It turned out that the k_1_–k_2_ pair is the best choice because it led to the catalytic efficiency value of 1.25 × 10^8^ M^−1^s^−1^, which is also inside the range 10^8^–10^10^ M^−1^s^−1^ for diffusion-limited enzyme reactions [67].

When normal noise is introduced only once in the forward rate constants k_1_, k_3_, and k_5_, with the proviso that the equilibrium constants K_1_* = k_1_*/k_2_, K_2_ = k_3_/k_4_, and K_3_* = k_5_/k_6_* do not change from their observed values, perfect proportionality follows for all efficiency–dissipation pairs (Figure S16). Due to the higher initial product concentration (see Table 4), the constraints K_1_* = const1, K_2_ = const2, and K_3_* = const3 are almost equivalent to the requirement that the initial equilibrium constants K_1_, K_2_, and K_3_ never change during the NetLogo simulation for Lac-1 β-lactamase kinetics. Since (k_cat_/K_M_)/dissipation expression depends only on equilibrium constants and the ratios of the rate constants (see Supplementary Text file, Equation (S2)), there is no reason for the slope change in the efficiency–dissipation dependence (Figure S16). The best efficiency value of 9.68 × 10^7^ M^−1^s^−1^ is close to the diffusion-limit range’s lower end (10^8^ M^−1^s^−1^). It was reached at the 2182nd tick for the X_tot_/RT = 8.23 and 3.7-fold higher turnover of k_cat_ = 7086 s^−1^.

### 7.4. Summary for *β-Lactamases*

To summarize, we have seen nearly perfect kinetic–thermodynamic proportionality for PC1 (Figure S10), RTEM (Figure S12), and Lac-1 (Figure S16). We constructed corresponding FORTRAN programs which confirmed it for all three β-lactamases (Figure 22). That is also the confirmation of an excellent efficiency–dissipation proportionality for the triosephosphate isomerase kinetics (Figure 3, Figure 5, Figure 6, Figure 8 and Figures S2 and S5) and for the results we obtained, but which did not show for KSI and CA isoenzymes. It will likely hold whenever the no-change condition is imposed for the equilibrium constants in all catalytic steps (see Supplementary Materials for more details). That conclusion did not change when the same Box–Muller transform (Equation (32) from Section 3) was used only once to introduce the noise in forward rate constants k_1_, k_3_, and k_5_.

The capture–release initial step leads to different relationships when the no-change condition is imposed on all first- and second-order rate constants, except k_1_ and k_2_ (Figures S11 and S15 and Figure 16 for β-lactamases). Noise was introduced twice in the corresponding NetLogo simulations—in the forward rate constant k_1_ and the backward rate constant k_2_. A fast enzyme efficiency increase can then occur for limited dissipation. The potential for the exponential-like efficiency increase is likely to be a general phenomenon for all Michaelis–Menten enzymes after lowering the activation barrier for the E+S→ES transition and increasing the activation barrier for the reverse ES→E+S transition.

**Figure 17:**
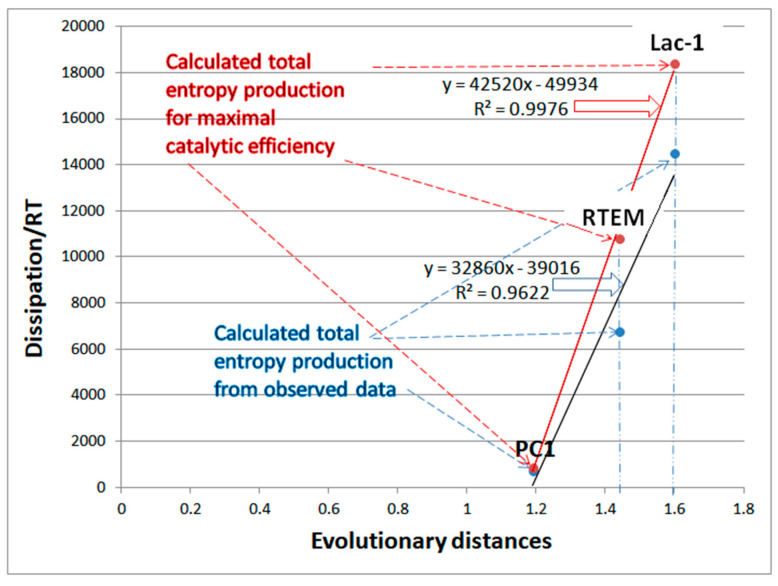
We compared evolutionary distances of 1.19, 1.44, and 1.60 for, respectively, β-lactamases PC1, RTEM, and Lac-1 [3,4,30] with numerical values for the total entropy production either calculated from experimental data (blue points) ([30] and Table 4) or for cases of maximal catalytic efficiency when normal noise is present in the E+S ↔ ES step (red points) (see Figures S11 and S15 and Figure 16). The figure illustrates the proportionality between overall entropy production and evolutionary distance when natural or artificial evolution produces the optimal or maximal possible catalytic efficiency.

Figure 17 illustrates the relationship between the evolutionary distance and overall entropy production for PC1, RTEM, and Lac-1 lactamase. We found blue points and a corresponding fit line (black) from the simulation of experimental data [3,30]. The dissipations associated with the red points (and red fit line) are from the best catalytic efficiency points in Figures S11 and S15 and Figure 16. The dissipation increased in an almost linear manner for more evolved β-lactamases. Noise introduction and searching for the highest enzyme efficiency confirmed the proportionality between the time passage (evolutionary distance) and overall entropy production. 

## 8. β-Galactosidase

β-galactosidase (βG, 3.2.1.23) also belongs to universal enzymes used by microbes and mammals. Microbial βG has a unique role in molecular biology, firstly due to Jacob and Monod’s model for the regulation of gene expression [117], secondly because of numerous molecular biology procedures using its bright blue reaction product, and thirdly for the confirmation of the Michaelis–Menten mechanism at the single-molecule level [118,119,120]. No less important is the βG role in the food industry [121]. The conventional βG use for preparing dairy products with reduced lactose content has been recently extended as a catalyst for lactose upgrading into valuable sweet glycosides, which support the growth of beneficial gut microbes [121,122]. In this subsection, we used published microscopic rate constants [26,119,123] to study how βG catalytic efficiency depends on its entropy production (Table 5). Our contribution to Table 5 was calculating all relevant kinetic and thermodynamic parameters using initial published values. For k_cat_/K_M_ and P, we verified that other authors obtained identical results using different methods (Case A from [18]).

In our NetLogo simulation, we first introduced noise only in the encounters among substrates and enzymes that form or dissociate the ES complex (Figure S17). It amounts to independent variations in k_1_ and k_2_. As expected, there was a steep increase in catalytic efficiency for the moderate dissipation increase, as we already observed for the substrate capture–release in the case of β-lactamases. The maximal efficiency point has the coordinates 10^4^ s^−1^ and 5.2 × 10^7^ M^−1^s^−1^ in the efficiency–dissipation plot (Figure S17). It is close to the point associated with the highest dissipation.

**Figure 18:**
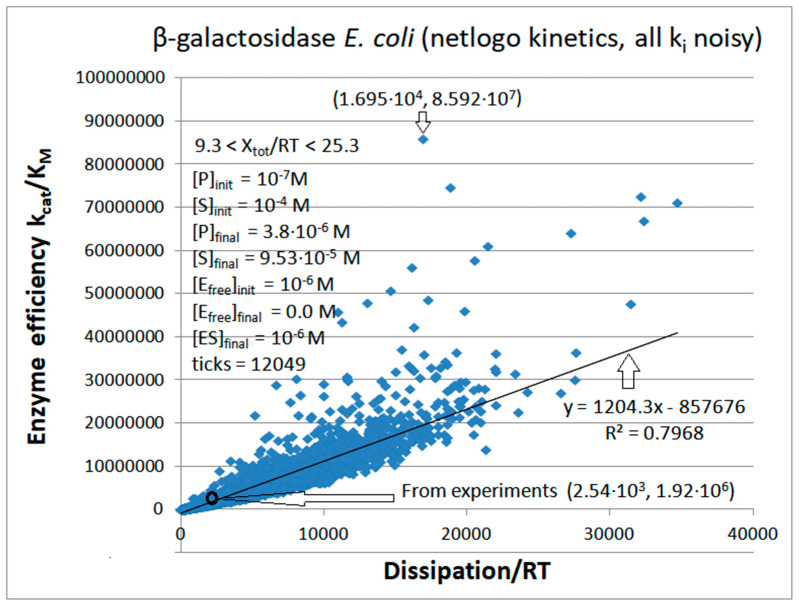
The catalytic efficiency dependence on dissipation when normal noise is introduced independently in all kinetic constants k_i_ for the β-galactosidase kinetics (see Section 3 and Table 5). We decreased the probability of stochastic jumps between enzyme conformational states and the enzyme to ligand association–dissociation to examine the initial system states with minor changes in substrate concentration. The highest catalytic efficiency was found at the 390th tick when the overall force was at the upper end of its range X_tot_/RT = 25.3. We used our Simulation-S27-beta-GAL-NetLogo software (see Supplementary Materials) to construct Figure 18.

**Figure 19:**
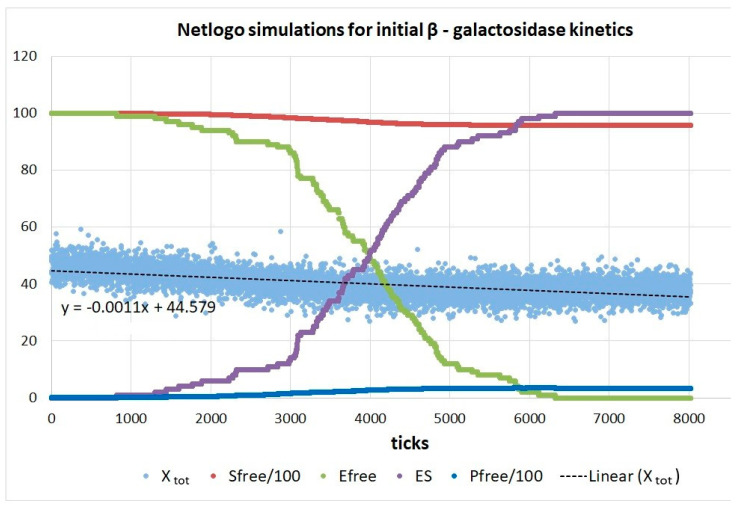
Force and concentration dependence on the time steps (ticks) through 8021 ticks from the second NetLogo simulation (see Table S1) as the system relaxes from the initial state values. From the 6323rd tick onward, all free enzymes have been converted into the ES complex. Still, the substrate-to-product conversion reached a stable state with a 28 times substrate excess, because we intentionally slowed down the conversion to increase the chance of finding the catalytic efficiency value inside the diffusion limit. We used the same Simulation-S27-beta-GAL-NetLogo software (see Supplementary Materials) to construct Figure 19.

The next task was to examine a vast efficiency–dissipation space by introducing changes in all four rate constants, k_i_ (Figure 18). The best value we found of k_cat_/K_M_ = 8.59 × 10^7^ M^−1^s^−1^ is close to the diffusion limit. The NetLogo runs are not completely reproducible. For instance, the second run with the identical agent-based software finds the better enzyme efficiency of 1.09 × 10^8^ M^−1^s^−1^ during a smaller number of re-setting steps (Figure 19 and Table S1).

Figure 19 illustrates how the concentrations of [S], [E], [ES], and [P] change in the second NetLogo simulation (see Table S1), together with noisy changes in the overall affinity (force) during 8021 ticks. Still, there is a slow relaxation of the overall force and dissipation during the program run (only the force relaxation is shown in Figure 19). Initially, faster and nonlinear relaxation occurs when transformations among different conformations are sped up.

We inspected all kinetic and thermodynamic parameters from the Simulation-S27-beta-GAL-NetLogo software output (see Supplementary Materials). Some of them are listed in Table S1. In the first run (Figure 18), a 40-fold decrease occurred in the rate constant k_2_ for the ES complex dissociation back to the free enzyme and free substrate (compared to Table 5 value). It resulted in a 40 times efficiency increase. The k_1_ increase up to three times also contributes to stronger substrate–enzyme binding and a simulation result of higher catalytic activity. The second conclusion from the NetLogo simulations presented in Figure 18 and Figure 19, and Table S1, is that significantly increased catalytic efficiency does not need maximal nor close to maximal dissipation. The third conclusion is that any means for increasing the irreversibility of the first catalytic step in the forward direction would increase the enzyme efficiency, since the enzyme already acts according to standard Michaelis–Menten kinetics by having the highly irreversible product-release step. We also presented the turnover numbers (k_cat_) in Table S1. In the two-state model for generalized (reversible) Michaelis–Menten kinetics, the turnover number k_cat_ equals the forward rate constant k_3_ for dissociating the ES complex into enzyme E and product P. Our NetLogo simulations did not change much the initial (observed) k_3_ = 730 s^−1^. Thus, the best enzyme efficiency increase in both Table S1 runs is mainly due to the considerably smaller Michaelis–Menten constant K_M_ than the observed K_M_ (see Table 5 and Table S1).

We also performed the FORTRAN simulation in the presence of noise with the same initial values (Figure S18). We called random numbers s_1_ and s_2_ only once (see Section 3). We multiplied the identical Box–Muller transform, gi, containing shift +1 with each of the four kinetic constants, k_i_, to eliminate the cases of negative k_i_. Two equilibrium constants, K_1_ and K_2_, went through small changes (decrease) because we allowed for the stepwise changes in the substrate and product concentrations. Mass conservation for ligands was approximately satisfied with the condition [S] + [P] = const for all ten thousand computational steps by our Simulation-S28-beta-GAL-FORTRAN software (see Supplementary Materials). We obtained the best result for the highest efficiency at the 9532nd step. It was less impressive (9.48 × 10^6^ M^−1^s^−1^) when compared to the best result from the NetLogo simulation. The force decrease during 10,000 steps exhibited a similar gradual decrease from higher initial values as in Figure 19.

**Figure 20:**
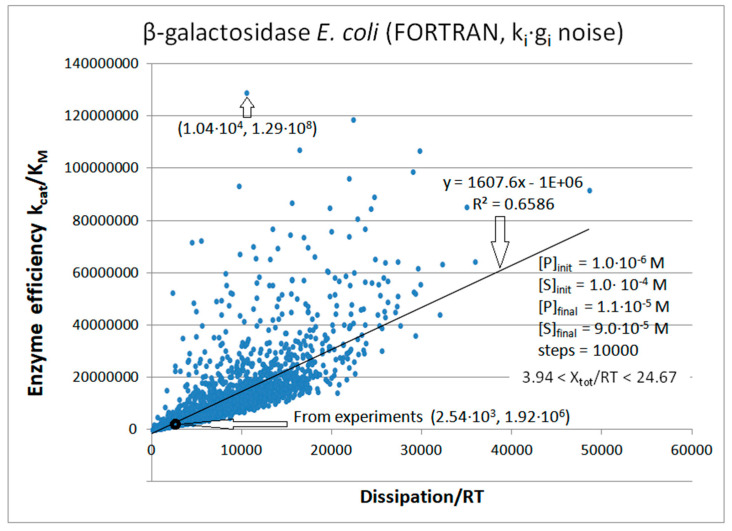
The catalytic efficiency dependence on dissipation when the normal noise function, g_i_, with shift +1 is called separately four times to multiply each rate constant, k_i_ (i = 1, 2, 3, 4), in our simulation for β-galactosidase kinetics. The highest catalytic efficiency was found at the 6540th computational step when the total force was X_tot_/RT = 20.8. We used our Simulation-S29-beta-GAL-FORTRAN software (see Supplementary Materials) to get the kinetic and thermodynamic parameters we needed to construct Figure 20.

A considerably more comprehensive search for the best dissipation–efficiency coordinate pairs occurred when we called the Box–Muller transform separately four times, once for each of the four rate constants, k_i_. The k_i_g_i_ products gained the freedom to vary independently from each other. The output of such a FORTRAN program (Figure 20) contains two catalytic efficiency values close to the lower range of the diffusion limit (10^8^ M^−1^s^−1^).

## 9. Glucose Isomerase

Glucose isomerase (GI abbreviation) fulfills nutritional requirements mainly in bacteria [124]. It is also known as xylose isomerase because GI reversibly isomerizes D-glucose and D-xylose to D-fructose and D-xylulose, respectively. Glucose-to-fructose conversion is relatively inefficient but critical for the commercial production of high-fructose corn syrup (HFCS) due to its specificity (the absence of nonmetabolizable or toxic side products) and mild ambient conditions [125]. Together with other industrial applications through the decades, such as bioethanol production [126], GI maintained a high market share in the food industry, among other industrial enzymes, despite its inherently low activity [124,127]. That is why there were frequent research efforts to use molecular engineering to improve GI performance for different applications [125,126,128,129,130]. Besides academic interest, that is also why theoretical research is devoted to enhancing GI performance when kinetic and thermodynamic limits are considered.

Considering previous examples for other enzymes, the best option is to initiate research with all the microscopic rate constants inferred from the observed data. Unfortunately, the best example [27] refers to GI preparation from *Streptomyces murinus*, which has very low measured activity. Nevertheless, the principles we employed in this section to significantly improve GI performance may be applicable for predicting the activity gains of the most promising GI variants for green industry applications. We also wanted to test our hypothesis about catalytic efficiency proportionality to dissipation by using the example of an inefficient enzyme working close to the thermodynamic equilibrium. The drawback is the restriction to the two-state model (Figure 1a); that is, the reversible Briggs–Haldane mechanism used in early and recent proposals for the kinetic mechanism of immobilized GI [27,131,132,133,134]. In the quasi-steady state, the solution is the Michaelis–Menten equation and the corresponding performance parameters k_cat_, K_M_, and k_cat_/K_M_. In our notation (Figure 1a), k_3_ = k_cat_ and K_M_ = (k_2_ + k_3_)/k_1s_, where k_1s_ is the second-order rate constant. Unusual experimental conditions used to determine these kinetic parameters include an elevated temperature (65 °C) besides the immobilization of enzymes (Table 6). These conditions are not responsible for the observed low k_cat_ and k_cat_/K_M_ values. If anything, they slightly increase the performance parameters.

Figure S19 results from the NetLogo simulation with noise independently introduced in all rate constants. It illustrates the absence of a strong proportionality relationship between catalytic efficiency and entropy production for an inefficient enzyme, such as glucose isomerase. The best point with the coordinates (0.21, 0.21) was found at the 1715th tick. It is associated with the Xtot/RT = 4.7, about 2.5-fold higher k_1_, 9-fold smaller k_2_, 24-fold higher equilibrium constant K_1_, and approximately 11-fold higher partial entropy production P_1_. Thus, the association and dissociation of the substrate with the enzyme should be shifted toward the ES complex formation to gain a significant 5.8-fold increase in the enzyme efficiency and a 2.3-fold increase in the turnover number.

Imposing some constraints on the system can recover the efficiency–dissipation relationship. For instance, rate constants k_1_ and k_2_ can be independently multiplied with the normal noise without changes in rate constants k_3_ and k_4_, other than those caused by the increased product concentration. The correlation R^2^ jumps to 0.881 for the k_cat_/K_M_ dependence on dissipation. However, the NetLogo simulation goes through a restricted search space and finds lower values for the best efficiency.

We also performed FORTRAN simulations to verify that different software and ways for noise introduction can still produce an approximately linear response in catalytic efficiency to the dissipation (Figure S20). Normal noise was called only once and used to multiply all four rate constants. The best catalytic efficiency of 0.18 M^−1^s^−1^ was comparable to the best result for the NetLogo simulations with noise. It was also considerably better than the 0.0215 M^−1^s^−1^ catalytic efficiency, easily calculated from the Dobovišek et al. optimization [28]. Incidentally, Dobovišek’s result was obtained for the positive force X_tot_/RT = 0.51 when the net flow was in the forward direction, and our two-state expressions for k_cat_ and K_M_ (see Section 2) are appropriate to use for the calculation of k_cat_/K_M_. It emerged due to the unique nature of the quasi-steady state that these authors obtained after imposing the no-change constraint for the product of forward rate constants: $K^{+}=k_{1}^{*}k_{3}$ = const.

**Figure 21:**
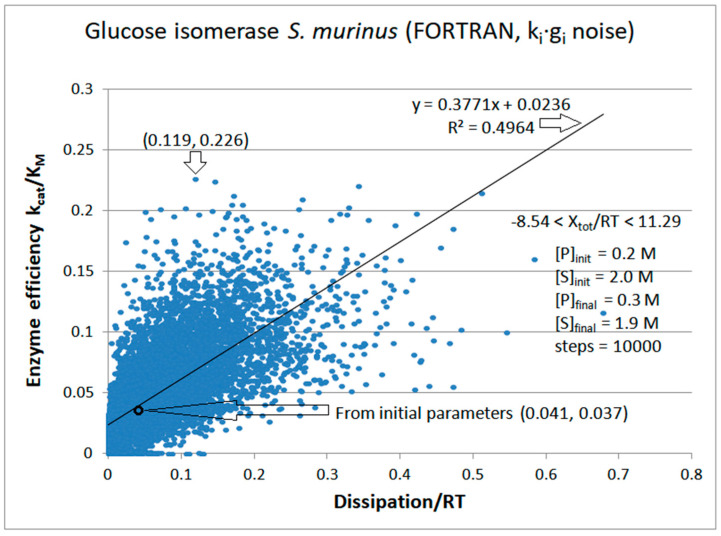
The catalytic efficiency dependence on dissipation when the normal noise function, g_i_, with shift +1 is called separately four times to multiply each rate constant, k_i_ (i = 1, 2, 3, 4), in the simulation for the glucose isomerase kinetics. The main loop from our Simulation-S32-GI-FORTRAN software (see Supplementary Materials) went through the 10,000 steps. We used its output to construct Figure 21.

Normal noise with shift +1 (see Section 3) was called four times in the next FORTRAN simulation so that each rate constant was multiplied with its own Box–Muller transform (Figure 21). The best catalytic efficiency of 0.226 M^−1^s^−1^ result was found for the lower overall dissipation in the RT units (0.12 s^−1^) compared to the previous NetLogo simulation (Figure S19).

## 10. An Overall Summary of All Results

When variations are allowed in concentrations and microscopic rate constants, an artificial evolution of enzymes is, in theory, possible. We explored the differences among reference parameters (calculated from the observed values) for ten studied enzymes and the best fold improvements for the catalytic efficiency k_cat_/K_M_ after noise introduction. The research idea was to examine corresponding changes in the overall entropy production. Is there any connection between the critical parameter for enzyme evolution and the most important parameter for the thermodynamic evolution of nonequilibrium systems? We provide the analysis of corresponding changes in kinetic–thermodynamic parameters in Table 7. We reached a simple conclusion after examining the best results for the increase in enzyme efficiency. There is no exception for increased dissipation. When noise is independently introduced in all of the microscopic rate constants, the improvement in the highest enzyme efficiency ranges from 4.5 to 67 times, while the entropy production increase ranges from 3 to 161 times in the cases where we used our FORTRAN source codes to perform simulations. For our NetLogo simulations, k_cat_/K_M_ improvements ranged from 5 to 45 times, while entropy production increases ranged from 4 to 198 times. Thus, for isothermal conditions, entropy production or dissipation can be regarded as the thermodynamic performance parameter, which indicates how efficient enzymes are in opening the gates for decreasing electrochemical gradients.

We visually picked up the conditions with high catalytic efficiency. That can be automated using an iterative procedure in which the best performance parameters are chosen as initial until no further improvements occur. Table 7 illustrates that enzymes differ in their evolutionary potential but have similar positive associations among their kinetic and thermodynamic performance parameters. Namely, a joint increase in k_cat_/K_M_ and total entropy production argues for the fundamental connection between more efficient free-energy transduction into essential biochemical reactions and the level of thermodynamic irreversibility.

Assuming that K_M_ does not change for a chosen enzyme, the observed proportionality between the enzyme’s entropy production and the specificity number, k_cat_/K_M_, implies a linear increase in catalytic efficiency, k_cat_, with dissipation. When noise is present in rate constants, approximate K_M_ constancy will still hold for no changes in the k_3_/k_1_ ratio (two-state Equation (24)), the k_5_/k_1_ and k_5_/k_4_ ratio (three-state Equation (27)), or the k_3_/k_1_, k_3_/k_5_, and k_5_/k_7_ ratio (four-state Equation (30)) when the equilibrium constants do not change. The turnover number, k_cat_, has a similar meaning and value to the cyclic flux J. We calculated entropy production as the bilinear JX product in thermodynamic forces X (in the RT units) and fluxes J (in inverse seconds). For the constant force X in isothermal conditions, entropy production and dissipation must be proportional to the flux J. Thus, k_cat_/K_M_ to the dissipation proportionality must hold for all conditions of small changes in K_M_ and slight differences between J and k_cat_ (see Supplementary Materials for more mathematical details).

**Table 7:** Fold improvement for enzyme efficiency and the corresponding fold increase in the overall dissipation in the best cases concerning values found from experiments. * The fold factor is the ratio of the best efficiency/dissipation and observed efficiency/dissipation.

Enzyme (# Functional States, Figure #)	Simulation Software Abbreviation (Noisy k_i_)	Efficiency Fold-Improv.	Dissipation Fold Increase	Eff/Disssip. (Fold Factor) *	Best Eff. (M^−1^s^−1^)
Glucose isomerase (2, 21)	S32-fortran (all k_i_ noisy)	6.1	2.9	1.9 (2.1)	0.226
Glucose isomerase (2, S19)	S30-netlogo (all k_i_ noisy)	5.8	5.2	1.0 (1.1)	0.213
β-galactosidase (2, 18)	S27-netlogo (all k_i_ noisy)	44.8	6.7	5.1 × 10^3^ (6.7)	8.59 × 10^7^
β-galactosidase (2, 20)	S29-fortran (all k_i_ noisy)	67.2	4.1	1.2 × 10^4^ (16.4)	1.29 × 10^8^
Lac-1 β-lactamase (3, S15)	S24-netlogo (noisy k_1_,k_2_)	4.8	1.3	6.4 × 10^3^ (3.6)	1.3 × 10^8^
RTEM β-lactamase (3, 16)	S21-netlogo (noisy k_1_,k_2_)	9.6	1.6	2.1 × 10^4^ (6.0)	2.3 × 10^8^
PC1 β-lactamase (3, S11)	S19-netlogo (noisy k_1_,k_2_)	6.5	1.2	7.6 × 10^4^ (5.2)	6.5 × 10^7^
Carbonic anhyd. I (4, S7)	S14-fortran (all k_i_ noisy)	4.5	18.1	218 (0.25)	1.1 × 10^8^
Carbonic anhyd. I (4, S8)	S15-netlogo (all k_i_ noisy)	5.9	22.4	231 (0.26)	1.47 × 10^8^
Carbonic anhyd. II (4, 14)	S16-netlogo (all k_i_ noisy)	5.1	13.4	254 (0.38)	4.25 × 10^8^
Carbonic anhyd. T200H (4, S9)	S17-netlogo (all k_i_ noisy)	5.5	14.0	421 (0.39)	3.71 × 10^8^
Ketosteroid isomerase (4, 10)	S10-fortran (all k_i_ noisy)	6.2	4.6	3.5 × 10^3^ (1.34)	1.88 × 10^9^
Ketosteroid isomerase (4, 12)	S13-netlogo (all k_i_ noisy)	8.6	3.8	5.7 × 10^3^ (2.26)	2.59 × 10^9^
Triophosphate isomerase (4, 7)	S7-fortran (all k_i_ noisy)	29.9	160.6	1.5 × 10^4^ (0.19)	2.4 × 10^7^
Triophosphate isomerase (4, 9)	S9-netlogo (all k_i_ noisy)	28.1	198.4	1.1 × 10^4^ (0.14)	2.2 × 10^7^

## 11. Discussion

### 11.1. Dissipation, Evolution, and the Catalytic Power of Enzymes

The evolution of the universe can be described as universal evolution. It created time, space, and myriad beautiful objects, such as galaxies, stars, planets, and living beings [135]. An invisible, but not less important, product of universal evolution is increased entropy and total entropy production. Evolution in physics is firmly connected to entropy production. A new phase of universal evolution started with the appearance of objects that can be associated with the massive jump in entropy production. The first living cells are one class of such objects, originating in an aqueous environment endowed with rich chemistry. The same volume of some bacterial cells, mitochondria, or chloroplast produces many orders of magnitude higher dissipation than an equivalent average volume of a sun-like star, despite the star’s much higher temperature [136,137]. The specific variety of complex life and mineralogy we enjoy here on Earth is not likely to exist anywhere else in the universe [138]. Thus, we should protect it, study it, and, if possible, understand it as a natural consequence of universal evolution.

Standard evolutionary theory [139] has a simple answer to the question of how new variations can arise: random mutations and natural selection ensure the adaptation of organisms to their environment. Thus, a particular noise class (chance genetic changes) is adapted to provide a better fit among organisms and environments. That view has been extended recently by considering the physicochemical evolutionary driving forces [140], including the maximization of dissipation [20].

Thermodynamic and biological evolution are connected. The major thermodynamic process for living cells is a large outflow of entropy [141]. Only a small portion of available free energy is used by cells for synthetic and mechanistic goals. For instance, the free energy converted into chemical bonds is a minor contribution compared to the free energy change from catabolism. Still, an almost perfect correlation exists between the total heat released and the amount of dry mass grown or the total amount of oxygen consumed during the aerobic growth of a yeast culture [142]. Theoretical studies also concluded that a higher maximal growth rate would be achieved by replicating a system capable of producing more heat [143,144]. Thus, higher entropy production can be an advantage during the evolution of organisms. As a rule, total entropy production reaches its maximum value before it decreases when microorganisms are fully supplied with free-energy sources and engaged in vigorous growth during their short-term evolution in batch experiments. This pattern is recapitulated in the life of every individual organism. Metabolic heat production per surface area reaches the maximal value early, with a subsequent decline over the lifetime [145].

Metabolism is the work of enzymes. Despite the enzymes’ complexity, they represent a “cleaner” opportunity than organisms for investigating evolution [146]. The simplest and most successful description of how many such enzymes work is generalized Michaelis–Menten kinetics [39,147,148,149]. The increased complexity of life through eons required the means for increasing the catalytic efficiency of such enzymes. Wolfenden and colleagues found with other authors that nothing makes a sharper distinction between life and nonlife than a massive jump in catalytic power, which enzymes show when the speed and specificity of the reaction they catalyze is compared to an equivalent reaction in the presence of inorganic catalysts [11,13,14,15,16,17,150,151,152]. The marvelous biochemistry of enzymes is tied to the evolutionary enhancements of enzymes’ catalytic rate (up to 10^26^-fold, according to Edwards et al. [17]). The corresponding catalytic proficiency for alkylsulfatase is an astronomical number: (k_cat_/K_M_)/k_uncat_ = 10^29^ M^−1^. Dynamic changes essential for understanding the catalytic activity of enzymes are challenging to trace structurally [153]. Structural studies did not help as much as we hoped in answering how enzymes work [154,155]. Since an increase in entropy production speeds up the physical evolution of any nonequilibrium system (it undergoes faster relaxation from the initial far-from-equilibrium state), we can assume a connection with the evolution of catalytic efficiency.

Martyushev and Seleznev [156] anticipated a fruitful connection between optimal kinetics parameters and entropy production for strongly nonequilibrium processes. However, it is surprising that the relationship between the overall dissipation and the frequently measured specificity constant k_cat_/K_M_ was never thoroughly examined. These two parameters connect laboratory biochemistry with the fundamental thermodynamics of nonequilibrium processes. Banerjee and Bhattacharyya’s finding [18] that the more efficient enzyme involves higher total dissipation is in accord with the results presented in this paper. The finding is based only on three pairs of dissipation–efficiency values for a single enzyme (β-galactosidase). Still, it is gratifying that their different method for calculating overall entropy production produced the same result (2553 s^−1^) as T. Hill’s approach [5], which we used in our FORTRAN and NetLogo programs for β-galactosidase (see the last row from Table 5). How changes in dissipation can lead to an increased catalytic efficiency k_cat_/K_M_ was not the main interest of these authors.

**Figure 22:**
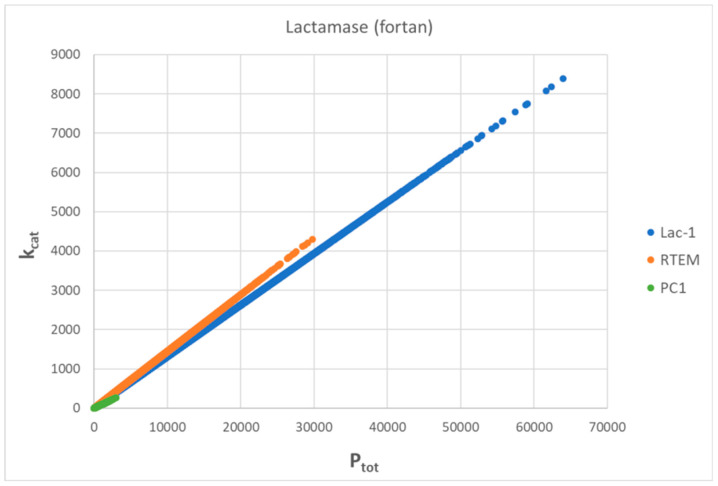
We performed simulations for k_cat_ dependence on the total entropy production in the case of three β-lactamases (PC1, RTEM, and Lac-1). Figure 22 presents our results after constructing three programs in the FORTRAN language. Each forward rate constant was multiplied with the identical normal noise function, while corresponding backward rate constants were determined from the no-change requirement to the equilibrium constants. Table 4 parameters were used for each enzyme. Concentrations were not allowed to change from their initial (observed) values. As for other figures, the P_tot_ label at the *x*-axis is the dissipation/RT in inverse seconds. The figure illustrates the proportional increase or decrease in the turnover number with dissipation from observed (calculated) points for PC1 (689, 61), RTEM (6757, 975), and Lac-1 (14,526, 1905) (see Table 4 and Juretić et al., 2019 [3,30]). The highest points have the coordinates (3035, 268) for PC1, (3 × 10^4^, 4303) for RTEM, and (6.4 × 10^4^, 8394) for Lac-1. As for catalytic efficiencies (Figure 17), both the observed and the highest points (dissipation, kcat) are nearly proportional to the evolutionary distance from the putative common ancestor in the order PC1 (1.19) < RTEM (1.44) < Lac-1 (1.60).

The main results from this paper presented the proportionality between k_cat_/K_M_ and overall dissipation for 10 different enzymes belonging to 6 different EC classifications. However, we could have shown k_cat_ proportionality with overall entropy production for each enzyme. One example of the proportionality between k_cat_ and dissipation is for three β-lactamases (Figure 22).

Besides the proportionality between k_cat_ and dissipation (in the units of inverse seconds), Figure 22 also illustrates the nearly linear connection between the evolutionary distances of PC1 (1.19), RTEM (1.44), and Lac-1 (1.60) lactamase and either k_cat_ or overall dissipation (see Figure 17, [3,30] for the evolutionary distances we put in parenthesis). We obtained the same result after comparing experimental results for the kinetic and thermodynamic parameters of A-class β-lactamases and looking for the maximal partial dissipation in the rate-limiting steps [3,4]. In these and other publications [30], we stressed that the optimization for high turnover numbers should be based on the physical principle of maximum transitional entropy production, not on the uncritical acceptance of the maximal catalytic efficiency or maximal catalytic constant as the selection or optimization criterion.

There was no need in the present study to make an a priory assumption of either a physical or biological principle reigning supreme. We only required some mechanism for reasonable variations in the microscopic rate constants. A crowded cellular milieu and unavoidable errors in translation and transcription offer several such means for noise introduction in kinetic parameters. Stochastic fluctuations are always present and are relevant for applying the Michaelis–Menten-type kinetics inside cells for small volumes and small numbers of interacting molecules [157,158]. Our simulations are, admittedly, a crude and artificial way of considering the noise. Better methods for dealing with physical and biological noise sources are undoubtedly possible. However, we were primarily interested in whether different means of noise introduction can uncover regular relationships between the most critical thermodynamic and kinetic parameters for highly active enzymes that work arbitrarily far from the equilibrium. Using thermal and nonthermal noise through stochastic fluctuations and dynamic disorder [159,160] may have been beneficial during biological evolution [161,162,163,164].

### 11.2. Computational Improvements of the Catalytic Power for Specific Enzymes

The catalytic power of enzymes is measured as k_cat_ or k_cat_/K_M_. Experts in the field did not object to the word “efficiency” when k_cat_/K_M_ was named catalytic or enzyme efficiency. However, it is not the efficiency of biological nanomotors in the range of 0 to 1. Some authors did not recommend using k_cat_/K_M_ as an index for comparing the catalytic effectiveness of enzymes [165]. The majority consensus is that k_cat_/K_M_ is the appropriate measure for the specificity of noncooperative Michaelis–Menten enzymes [23,166,167]. In rare cases, when all microscopic rate constants have been determined [168], k_cat_ and k_cat_/K_M_ can be connected to partial and total entropy production when an enzyme reversibly cycles through all of its functionally important conformations (this work, [3,4]). Moreover, after variations in rate constants around their observed values, we can analyze optimal rate constants k_i_ and dissipations associated with the highest performance parameters k_cat_ and k_cat_/K_M_. What are, if any, the common features of the states with the highest enzyme efficiency, and how do the thermodynamic and kinetic parameters of these states differ from the same values calculated or inferred from the experimental data? Table 8 helps deal with that question. Our choice in this paper was to examine the best k_cat_/K_M_ values for corresponding k_i_, partial, and total dissipation. Table 8 gives the partial entropy production in the first forward catalytic step, because it exhibited the highest increase regarding the observed value. That is the consequence of an increased forward rate constant k_1_ and decreased backward constant k_2_ for substrate-to-enzyme association and dissociation.

In the case of triosephosphate isomerase (TPI), there was a 1454-fold increase in the partial entropy production P_1_ for the E+S↔ES transition, which became a 42% instead of 6% contribution to the total dissipation. We used our Simulation-S9-TPI-NetLogo software (see Supplementary Materials) to produce the results for constructing Figure 9, but other results from the same program gave values for all partial entropy productions. Figure 9 results associate positive force with a significantly increased flux J = 461.40 and catalytic constant k_cat_ = 1085.02 s^−1^ regarding the observed values (see Table 1). After maximizing the total entropy production density, the result from Šterk et al. [76] was J = −1272 s^−1^ (the blue point at the vertical line in the main Figure 4). It is about a 100 times higher net reaction flow in the reverse GAP→DHAP direction when compared to the experimentally observed reaction rate J = 14 s^−1^ facilitating glycolysis [32,74]. Šterk et al. [76] used the steady-state concentrations [S] = 31.45 and [P] = 8.55 μM and a controversial constraint [75] on all forward rate constants. The corresponding force was then highly negative at X_tot_/RT = −4.47. When multiplied with the high negative flux, it produced such a high dissipation that the optimal values (5685, 1.8 × 10^6^) could not be illustrated as the (x, y) point within the confines of Figure 6. The authors found the maximum in the overall entropy production, but it was about 570-fold higher than the calculated value from the experimental data. Klipp and Heinrich [77] obtained an even higher net reaction flow in the backward GAP→DHAP direction ranging from J = −1620 (for the experimental rate constant values when [DHAP] = [GAP] = 40 μM) to J = −4010 s^−1^ (for the separate limit optimization model), the result that was verified and commented on by Bish and Mavrovouniotis [78]. These optimizations for highly negative flux and negative force can only ensure the nonphysiological operation of the TPI enzyme and the loss of its primary function of balancing glycolysis and gluconeogenesis. For instance, the optimized k_cat_ in the forward direction of Šterk et al. [76] is k_cat_ = 222 s^−1^, which is worse than k_cat_ = 432 s^−1^ (experimental data [32]). In contrast, our optimized k_cat_ = 686 s^−1^ [74] and k_cat_ = 1085.02 s^−1^ (this paper) are improvements over the k_cat_ value calculated from the experimental data.

For other enzymes and software simulations in the presence of noise, P_1_ increased one to two orders of magnitude, and its percentage also increased for the best enzyme efficiency results. The single exception is carbonic anhydrase. For CA I, CA II, and the T200H CA II mutant, an absolute increase in P_1_ was not accompanied by an increase in its percentage. A possible reason is a different kinetic scheme for the CA enzyme (Figure 13) and an inadequacy of the standard k_cat_ and k_cat_/K_M_ expression (Equations (29)–(31)) for that scheme.

The best efficiency fold improvement is seen for β-galactosidase, which also reaches the highest efficiency-to-dissipation fold ratio (Table 7). However, ketosteroid isomerase has the best evolutionary potential in our simulations. That can be connected to the two proton transfer reactions catalyzed by KSI [169,170,171] and a powerful electric field [172]. Electric field catalysis needs a strong and correctly oriented field. The measured field of 1.44 × 10^10^ V/m is enough to account for 72% of the total acceleration rate [172]. The transient appearance of billions of volts per meter electric field strength in the interior of active proton-shuffling enzymes frequently speeds up catalysis [4]. The isomerization of 5-androstene-3,17-dione in solution through the same mechanism utilized by KSI is slow. That is why KSI catalytic proficiency is so high. As mentioned in the KSI section, Radzicka and Wolfenden [13] estimated it as 1.8 × 10^15^ M^−1^ based on k_uncat_ = 6 × 10^−7^ s^−1^.

Interestingly, the efficiency fold improvement (Table 7) is similar for the best (KSI) and worst enzymes (glucose isomerase). The k_cat_ = 0.029 s^−1^ (experimental) and k_cat_ = 0.031 s^−1^ or 0.068 s^−1^ (optimal) values for the GI enzyme (see k_3_ results in Table 8) are two orders of magnitude smaller than the turnover numbers 2 s^−1^ and 11 s^−1^ reported in the literature [128,173]. The Converti et al. [27] data we used to initiate simulations pertain to weakly active GI working close to the thermodynamic equilibrium. Nevertheless, our method for the theoretical increase in catalytic activity is robust enough to ensure its close to a 10-fold increase (from 0.0365 to 0.2262 M^−1^s^−1^, Figure 21). In conclusion, the present analysis of the role of total entropy production extends previous approaches to optimizing enzyme kinetics using the maximization of partial entropy production [3,4].

### 11.3. Possible Benefits of Considering Unanswered Questions

Most enzymes did not use their potential to evolve higher catalytic efficiencies due to the absence of selection pressure to maximize it for individual enzymes [19]. When metabolic demand existed, the superstars of enzyme evolution developed, often named perfect enzymes [168]. Our simulations suggested the theoretical possibility of increasing the k_cat_/K_M_ of either moderately efficient or perfect enzymes. In practice, more than one amino acid substitution is needed to improve the performance parameters. Several orders of magnitude improvement typically require at least 5 to 10 beneficial mutations [174].

Living far from equilibrium is an essential asymmetry of present-day life [175,176]. Higher dissipation increased the catalytic efficiency of the enzymes we explored in this paper and the system’s distance from thermodynamic equilibrium. The plausible inference is that some abiotic driving forces, such as proton gradients in alkaline hydrothermal vents, must have operated to maintain far-from-equilibrium situations and high entropy production during the emergence of life on Earth. According to that assumption, bioenergetics and vectorial biochemistry are older than the genetic code and the first universal common ancestor [3]. It enabled the enzymeless and cell-less synthesis of amino acids, sugars, nucleotides, and lipids. Nonlinearity and far-from-equilibrium conditions are two requirements for driving the protometabolism toward autocatalysis and self-organization. The accelerated accumulation of organic molecules followed in the presence of the long-lived abiotic protonmotive force to jump-start the development of life [177]. The efficiency of organic synthesis with protoenzymes was likely low compared to present-day enzymatic catalysis. However, such self-reinforcing reactions increased the efficiency of dissipating available free-energy gradients. The present-day connection between dissipation and catalytic efficiency we studied in this paper is thus likely to reflect the linkage between the higher dissipation potential and the accelerated synthesis of ever more complex organic compounds, which was already present at the origin of life. Entropy production increases faster due to an enzyme’s activity, albeit in the microscopic world.

Within biology, we cannot find the answer to why dissipation was crucial for the emergence of life, as it is essential for the present-day catalytic efficiency of uni–uni enzymes. Can entropy production have an autocatalytic role too? Namely, did increased entropy production promote the selection of the organic structures capable of increasing entropy production? That question has yet to be answered in the biophysics or the physics of nonequilibrium processes. The evolution of all systems in the universe may be coupled with decreasing their free energy in the least possible time [178]. Thus, living systems and biological macromolecules can be regarded as manifestations of physical principles about dissipation intensity rather than ends in themselves [179].

We mainly dealt with the academic interest in answering how measured kinetic parameters are connected to an enzyme’s entropy production. However, there is also a practical goal of enhancing the desired activity of natural enzymes or competing with nature in the rational design of artificial enzymes with better catalytic performance. These research fields are still in their infancy. Microwave irradiation can enhance enzyme activity and entropy production under chemiosmotic conditions [180]. A vortex fluidics device using pressure waves contained within thin films displayed an increase in enzyme efficiency for β-glucosidase and three other enzymes [181]. Faster protein motion can accelerate catalysis, while higher catalytic efficiency and additional heat released in the reaction can speed up the enzyme diffusion [9]. For instance, the catalysis of the exergonic enzymes (ΔG < 0) induced enhanced diffusion, which exhibited a striking proportionality to the energy release rate [182]. Also, the enhancement in biochemical and physical parameters can result from distal mutations that do not change individual equilibrium constants for each catalytic step or the overall equilibrium constant of the reaction [183]. All ways and means for hypothetic positive feedback between Gibbs energy release during enzyme catalysis, nonthermal motion, and increased enzyme performance parameters are likely to be strictly regulated in the cellular environment [184].

De novo enzyme design for green chemistry and medical goals has a huge potential [185,186,187,188,189,190]. It has been recently explored by combining computational methods and directed evolution experiments [191]. Still, something needs to be added to our insights about enzymatic catalysis. Artificial enzymes are generally inferior in catalytic efficiency compared to their natural counterparts [187]. While the role of reorganization energy is recognized in rational protein design [187], that is not the case with the catalytic efficiency to dissipation proportionality for the uni–uni enzymes we described in this paper. After all other means are employed to identify possible beneficial mutations for increasing catalytic efficiency with a given substrate, the computer-aided enzyme design can be extended with an additional selection for higher overall entropy production*.* In principle, mutations can be predicted based on their contribution to total entropy production, not only their contribution to transition state stabilization and reorganization energy.

**Table 8:** Kinetic and thermodynamic parameters for the best NetLogo and FORTRAN results concerning the values found from experiments. The green highlight denotes increased values, yellow denotes decreased values, and orange equals the experimental values.

Figure	Enzyme	Software	k_1_ (s^−1^)	k_2_ (s^−1^)	P_1_ (s^−1^) (%P)	P (s^−1^)	k_3_ (s^−1^)	k_4_ (s^−1^)	k_5_ (s^−1^)	k_6_ (s^−1^)	k_7_ (s^−1^)	k_8_ (s^−1^)
	**TPI**	Exper&calc.	400	7.0 × 10^3^	0.573 (6)	9.883	2.0 × 10^3^	6.0 × 10^3^	6.0 × 10^4^	9.0 × 10^4^	4 × 10^3^	25.60
**Figure 9**		NetLogo	1.05 × 10^3^	303	833 (42)	1.96 × 10^3^	5.4 × 10^3^	12.6 × 10^3^	9.4 × 10^4^	9.97 × 10^4^	6.5 × 10^3^	128
**Figure 7**		FORTRAN	1.14 × 10^3^	126	435 (27)	1.59 × 10^3^	1.05 × 10^3^	6.0 × 10^3^	6.0 × 10^4^	6.15 × 10^3^	937	25.60
	**KSI**	Exper&calc.	8.3 × 10^4^	8.6 × 10^4^	6.22 × 10^3^ (5) (5)	1.16 × 10^5^	1.8 × 10^5^	1.7 × 10^6^	6.4 × 10^5^	43	1.5 × 10^5^	5.0 × 10^4^
**Figure 12**		NetLogo	2.77 × 10^5^	3.7 × 10^4^	7.9 × 10^4^ (18)	4.50 × 10^5^	4.95 × 10^5^	7.1 × 10^5^	1.02 × 10^6^	16	6.9 × 10^4^	7.7 × 10^4^
**Figure 10**		FORTRAN	2.30 × 10^5^	2.5 × 10^4^	7.17 × 10^4^ (13)	5.39 × 10^5^	1.8 × 10^5^	3.97 × 10^5^	6.4 × 10^5^	43	1.5 × 10^5^	2.9 × 10^4^
	**CA I**	Exper&calc.	4.08 × 10^4^	3.8 × 10^4^	1.48 × 10^4^ (52)	2.84 × 10^4^	2.9 × 10^5^	6.24 × 10^5^	9.0 × 10^5^	9.0 × 10^6^	5.5 × 10^6^	4.5 × 10^4^
**Figure S8**		NetLogo	2.0 × 10^5^	2.3 × 10^4^	2.51 × 10^5^(40)	6.36 × 10^5^	4.7 × 10^5^	6.55 × 10^5^	2.0 × 10^6^	9.3 × 10^6^	8.8 × 10^6^	1.8 × 10^4^
**Figure S7**		FORTRAN	1.53 × 10^5^	1.5 × 10^4^	2.13 × 10^5^ (41)	5.14 × 10^5^	1.95 × 10^6^	7.71 × 10^5^	2.3 × 10^6^	8.5 × 10^6^	6.6 × 10^6^	3.1 × 10^4^
	**CA II**	Exper&calc.	1.56 × 10^5^	1.8 × 10^6^	5.33 × 10^4^ (43)	1.25 × 10^5^	1.7 × 10^7^	4.80 × 10^6^	1.2 × 10^6^	1.2 × 10^6^	2.0 × 10^7^	1.0 × 10^6^
**Figure 14**		NetLogo	6.38 × 10^5^	2.5 × 10^6^	5.61 × 10^5^ (34)	1.67 × 10^6^	3.7 × 10^7^	3.93 × 10^6^	1.5 × 10^6^	1.5 × 10^6^	3.0 × 10^7^	6.6 × 10^5^
	**CA II T200H**	Exper&calc.	9.84 × 10^4^	5.4 × 10^4^	4.03 × 10^4^ (64)	6.3 × 10^4^	3.0 × 10^5^	2.16 × 10^5^	2.7 × 10^6^	2.1 × 10^7^	1.8 × 10^7^	9.0 × 10^5^
**Figure S9**		NetLogo	6.49 × 10^5^	6.7 × 10^4^	4.05 × 10^5^(46)	8.82 × 10^5^	7.98 × 10^5^	7.4 × 10^4^	3.2 × 10^6^	2.9 × 10^7^	7.4 × 10^6^	4.6 × 10^5^
	**PC1**	Exper&calc.	3.28 × 10^4^	196	37 (5)	689	173	4.0	96	8.0		
**Figure S11**		NetLogo	1.15 × 10^5^	32	111 (13)	858	173	4	96	11		
	**RTEM**	Exper&calc.	1.71 × 10^5^	1.18 × 10^4^	185 (3)	6.76 × 10^3^	2.8 × 10^3^	6.0	1.5 × 10^3^	4.4 × 10^3^		
**Figure 16**		NetLogo	4.07 × 10^5^	851	1.4 × 10^3^ (13)	1.08 × 10^4^	2.8 × 10^3^	6	1.5 × 10^3^	4.7 × 10^3^		
	**Lac-1**	Exper&calc.	5.27 × 10^4^	2.32 × 10^3^	1.8 × 10^3^ (12)	1.45 × 10^4^	4.09 × 10^3^	50	3.61 × 10^3^	1.72 × 10^3^		
**Figure S15**		NetLogo	1.98 × 10^5^	976	3.1 × 10^3^ (16)	1.95 × 10^4^	4.09 × 10^3^	50	3.61 × 10^3^	1.76 × 10^3^		
	**β-galacto-** **sidas**	Exper&calc.	5.0 × 10^3^	1.83 × 10^4^	5.84 (0.2)	2.55 × 10^3^	730	1.0 × 10^−5^				
**Figure 18**		NetLogo	1.4 × 10^4^	467	628 (4)	1.70 × 10^4^	726	2.25 × 10^−7^				
**Figure 20**		FORTRAN	1.3 × 10^4^	61	1.12 × 10^3^ (11)	1.04 × 10^4^	520	0.0001				
	**Glucose isomerase**	Exper&calc.	0.126	0.021	0.0126 (31)	0.0392	0.029	0.016				
**Figure S19**		NetLogo	0.320	0.002	0.143 (68)	0.211749	0.068	0.088				
**Figure 21**		FORTRAN	0.499	0.004	0.057 (48)	0.119	0.031	0.045				

## 12. Conclusions

Our results stress the hallmark of uni-cycle enzymes as dissipation gates. Enzymes are not Maxwell’s demons that fight the mechanical tendency toward disorder, as Jacob argued in his book *The Logic of Life* [192]. Just the opposite, enzymes open the gates for the incomparably faster equilibration of concentrations than in their absence. When such gates opened during biological evolution, they sped up the spontaneous free-energy transduction into dissipative catalytic cycling by many orders of magnitude.

Selecting enzyme structures exhibiting high catalytic efficiency, k_cat_/K_M_, is the hallmark of biological evolution through natural selection. Together with the production of small molecules essential for life, it is indeed an order-creating function of enzymes. Still, it arises through opening the dissipation gates for a vast increase in dissipation. A search to open dissipation avalanches implies random structural changes (mutations) and a way to simultaneously fix the advantageous changes causing higher enzyme efficiency and dissipation. Thus, random noise and the increase in overall entropy production are prerequisites rather than hindrances to the evolution of complex life.

There are no known rules for repeating the miracle of biological evolution in increasing or improving enzyme efficiency [174,193]. However, a better connection of observed performance parameters with overall or partial dissipation and introducing dynamic disorder can help find such rules. Among other enzymes, we performed simulations with five well-known “perfect” enzymes cycling through generalized Michaelis–Menten-type kinetics near the diffusion limit. Increased catalytic efficiency and increased total entropy production go hand-in-hand, and there exists the scope for the further improvement of catalytic efficiency, even for the enzyme stars of biological evolution, with entropy production providing a thermodynamic measure of this improvement. The take-home message is that increased catalytic efficiency is connected to higher entropy production.

The changes in enzyme activity and specificity depend on noise-channeling constraints. Enzyme efficiency is more or less proportional to overall entropy production when we allow less or more freedom in the choice of restrictions. The efficiency–dissipation proportionality is perfect when we do not permit change in the driving force and equilibrium constants in each catalytic step. When translated into biological terms, it is the requirement that identical enzymes work in steady- or quasi-steady-state homeostatic conditions.

Dissecting entropy production contributions suggested the formation of the Michaelian complex ES as the critical catalytic step. An increased equilibrium constant for the substrate–enzyme association can increase the catalytic efficiency in the forward direction (S→P), the partial entropy production of that step, and the overall dissipation better than other means for increasing the activity for most enzymes.

Thus, within physics, we can find the answer to why dissipation was crucial for the emergence of life, as it is essential for the present-day catalytic efficiency of uni–uni enzymes. It is impossible to separate the enzyme catalytic rate, efficiency, or power from its overall dissipation. The question for further research regards increased catalytic efficiency as the outcome of higher entropy production. The proportionality between evolutionary distances, kinetic parameters, and dissipation also merits further investigation. We postulate that biological evolution proceeded within the laws of universal thermodynamic evolution, but with the ability to accelerate the latter. The origin of enzymes’ prodigious catalytic power is the synergy between thermodynamic and biological evolution. If increasing enzyme efficiency is the natural evolutionary target for some enzymes and the target for the directed evolution of designed enzymes, researchers can explore beneficial mutations based on their contribution to partial and total entropy production.

## Figures and Tables

**Table 1:** Calculated microscopic rate constants and kinetic parameters from the experimental data [32] in the case of the TPI isomerase-catalyzed conversion of DHAP (substrate) to GAP (product) at about 20 °C. The substrate and product concentrations were, respectively, [S] = 40 μM and [P] = 0.064 μM. The initial values presented in this table are identical to those we published in our previous computational optimization of TPI kinetics [74].

Rate Constants	Observed Values [32,74]	Kinetic and Thermod. Parameters	Calculated Initial Values [74]
k_1_*	10^7^ M^−1^s^−1^	[S]	4 × 10^−5^ M
**k_2_**	7000 s^−1^	[P]	6.4 × 10^−8^ M
**k_3_**	2000 s^−1^	[E]	5 × 10^−8^ M
**k_4_**	6000 s^−1^	k_cat_	432 s^−1^
**k_5_**	60,000 s^−1^	K_M_	5.5 × 10^−4^ M
**k_6_**	90,000 s^−1^	k_cat_/K_M_	7.86 × 10^5^ M^−1^s^−1^
**k_7_**	4000 s^−1^	K_eqtot_	3.2 × 10^−3^
k_8_*	4 × 10^8^ M^−1^s^−1^	X_tot_/RT	0.685
k_1_ = k_1_* · [S]	400 s^−1^	P ($\frac{Dissipation}{RT}$)	9.9 s^−1^
k_8_ = k_8_* · [P]	25.60 s^−1^		

**Table 2:** Calculated microscopic rate constants and kinetic parameters from experimental data [89] and the global optimization of experimental data [31] in the case of the 3-oxo-Δ^5^-steroid isomerase catalyzed conversion of 5-androstene-3,17-dione (substrate) to 4-androstene-3,17-dione (product) at 25 °C.

Rate Constants	Calculated Values [89]	Calculated Values [31]	Kinetic and Thermod. Parameters [31]	Initial Values ([31] and This Work)
k_1_*	8.6 × 10^8^ M^−1^s^−1^	8.3 × 10^8^ M^−1^s^−1^	**[S]**	10^−4^ M
**k_2_**	8.6 × 10^4^ s^−1^	8.6 × 10^4^ s^−1^	**[P]**	5 × 10^−5^ M
**k_3_**	1.7 × 10^5^ s^−1^	1.8 × 10^5^ s^−1^	**[E]**	5 × 10^−6^ M
**k_4_**	>3 × 10^5^ s^−1^	1.7 × 10^6^ s^−1^	**k_cat_**	3.5 × 10^4^ s^−1^
**k_5_**	>1 × 10^5^ s^−1^	6.4 × 10^5^ s^−1^	**K_M_**	1.16 × 10^−4^ M
**k_6_**	40 s^−1^	43 s^−1^	**k_cat_/K_M_**	3 × 10^8^ M^−1^s^−1^
**k_7_**	1.3 × 10^5^ s^−1^	1.5 × 10^5^ s^−1^	**K_eqtot_**	2281
k_8_*	8.6 × 10^8^ M^−1^s^−1^	1 × 10^9^ M^−1^s^−1^	**X_tot_/RT**	8.426
**k_1_**		8.3 × 10^4^ s^−1^	$\frac{Dissipation}{RT}$	Initial value (this paper)
k_8_		5 × 10^4^ s^−1^	P	1.16 × 10^5^ s^−1^

**Table 3:** Calculated microscopic rate constants and kinetic parameters from experimental data [33] (Behravan-1990) in the case of substrate (CO_2_) to product $\left({HCO}_{3}^{-}\right)$ interconversion and the proton-transfer buffer (B)-dependent step catalyzed by carbonic anhydrase isoenzymes at 25 °C.

Rate Constants [33]	Initial Values CA I	Initial Values CA II	Initial Values CA II T200H
k_1_*	3.4 × 10^7^ M^−1^s^−1^	1.3 × 10^8^ M^−1^s^−1^	8.2 × 10^7^ M^−1^s^−1^
**k_2_**	3.8 × 10^4^ s^−1^	1.8 × 10^6^ s^−1^	5.4 × 10^4^ s^−1^
**k_3_**	2.9 × 10^5^ s^−1^	1.7 × 10^7^ s^−1^	3.0 × 10^5^ s^−1^
k_4_*	2.6 × 10^7^ M^−1^s^−1^	2.0 × 10^8^ M^−1^s^−1^	9.0 × 10^6^ M^−1^s^−1^
**k_5_**	9.0 × 10^5^ s^−1^	1.2 × 10^6^ s^−1^	2.7 × 10^6^ s^−1^
**k_6_**	9.0 × 10^6^ s^−1^	1.2 × 10^6^ s^−1^	2.1 × 10^7^ s^−1^
k_7_*	1.1 × 10^8^ M^−1^s^−1^	4.0 × 10^8^ M^−1^s^−1^	3.6 × 10^8^ M^−1^s^−1^
k_8_*	9.0 × 10^5^ M^−1^s^−1^	2.0 × 10^7^ M^−1^s^−1^	1.8 × 10^7^ M^−1^s^−1^
**k_1_**	4.08 × 10^4^ s^−1^	1.56 × 10^5^ s^−1^	9.84 × 10^4^ s^−1^
**k_4_**	6.24 × 10^5^ s^−1^	4.80 × 10^6^ s^−1^	2.16 × 10^5^ s^−1^
**k_7_**	5.50 × 10^6^ s^−1^	2.00 × 10^7^ s^−1^	1.80 × 10^7^ s^−1^
**k_8_**	4.50 × 10^4^ s^−1^	1.00 × 10^6^ s^−1^	9.00 × 10^5^ s^−1^
**Kinetic parameters**	**Our initial and calculated values (CA I)**	**Our initial and calculated values (CA II)**	**Our initial and calculated values (CA II T200H)**
**[S]**	1.2 × 10^−3^ M	1.2 × 10^−3^ M	1.2 × 10^−3^ M
**[P]**	2.4 × 10^−2^ M	2.4 × 10^−2^ M	2.4 × 10^−2^ M
**[B]**	5.0 × 10^−2^ M	5.0 × 10^−2^ M	5.0 × 10^−2^ M
**[E]**	1.0 × 10^−4^ M	1.0 × 10^−4^ M	1.0 × 10^−4^ M
**k_cat_**	7.77 × 10^4^ s^−1^	8.05 × 10^5^ s^−1^	2.10 × 10^5^ s^−1^
**K_M_**	3.13 × 10^−3^ M	9.63 × 10^−3^ M	3.10 × 10^−3^ M
**k_cat_/K_M_**	2.48 × 10^7^ M^−1^s^−1^	8.36 × 10^7^ M^−1^s^−1^	6.77 × 10^7^ M^−1^s^−1^
**K_eqtot_**	6.10	6.14	6.51
**X_tot_/RT**	1.81	1.81	1.87
$\frac{Dissipation}{RT}$	CA I (this paper)	CA II (this paper)	CA II T200H (this paper)
P_initial_	2.84 × 10^4^ s^−1^	1.25 × 10^5^ s^−1^	6.29 × 10^4^ s^−1^

**Table 4:** Calculated microscopic rate constants, performance parameters, and dissipation from experimental data [4,29,30] in the case of benzylpenicillin substrate hydrolysis catalyzed at 20 °C by the A-class β-lactamases.

Rate Constants [3]	Observed Values, PC1	Observed Values, RTEM	Observed Values, Lac-1
k_1_*	2.2 × 10^7^ M^−1^s^−1^	1.23 × 10^8^ M^−1^s^−1^	4.1 × 10^7^ M^−1^s^−1^
**k_2_**	196 s^−1^	1.18 × 10^4^ s^−1^	2.32 × 10^3^ s^−1^
**k_3_**	173 s^−1^	2.8 × 10^3^ s^−1^	4.09 × 10^3^ s^−1^
**k_4_**	4.0 s^−1^	6.0 s^−1^	50 s^−1^
**k_5_**	96 s^−1^	1.5 × 10^3^ s^−1^	3.61 × 10^3^ s^−1^
k_6_*	1.0 × 10^6^ M^−1^s^−1^	4.0 × 10^7^ M^−1^s^−1^	8.0 × 10^6^ M^−1^s^−1^
**k_1_**	3.28 × 10^4^ s^−1^	1.71 × 10^5^ s^−1^	5.27 × 10^4^ s^−1^
**k_6_**	8.0 s^−1^	4.4 × 10^3^ s^−1^	1.72 × 10^3^ s^−1^
**Kinetic parameters**	**Initial values** **PC1** **(this paper)**	**Initial values RTEM** **(this paper)**	**Initial values** **Lac-1** **(this paper)**
**[S]**	1.492 × 10^−3^ M	1.390 × 10^−3^ M	1.285 × 10^−3^ M
**[P]**	8.0 × 10^−6^ M	1.1 × 10^−4^ M	2.15 × 10^−4^ M
**[E]**	10^−5^ M	10^−5^ M	10^−5^ M
**k_cat_**	61 s^−1^	9.75 × 10^2^ s^−1^	1.91 × 10^3^ s^−1^
**K_M_**	6.0 × 10^−6^ M	4.15 × 10^−5^ M	7.32 × 10^−5^ M
**k_cat_/K_M_**	1.01 × 10^7^ M^−1^s^−1^	2.35 × 10^7^ M^−1^s^−1^	2.60 × 10^7^ M^−1^s^−1^
**K_eqtot_**	8.69 × 10^4^	2.3 × 10^3^	3.9 × 10^3^
**X_tot_/RT**	11.4	7.74	8.3
$\frac{Dissipation}{RT}$	Initial value PC1 [3]	Initial value RTEM [3]	Initial value Lac-1 [3]
P	689 s^−1^	6757 s^−1^	14,526 s^−1^

**Table 5:** Initial values of microscopic rate constants from experimental data [26] and our calculations of other initial kinetic and thermodynamic parameters in the case of the *E. coli* β-galactosidase-catalyzed conversion of resorufin-b-D-galactopyranoside (substrate) to a fluorescent resorufin (product) at 25 °C.

Rate Constants	Initial Values (This Work and [26])	Initial Kinetic Parameters	This Work
k_1_*	5.0 × 10^7^ M^−1^s^−1^	**[S]**	10^−4^ M
**k_2_**	1.83 × 10^4^ s^−1^	**[P]**	10^−7^ M
**k_3_**	7.3 × 10^2^ s^−1^	**[E]**	10^−6^ M
k_4_*	10 M^−1^s^−1^	**k_cat_**	730 s^−1^
**k_1_**	5.0 × 10^3^ s^−1^	**K_M_**	3.81 × 10^−4^ M
**k_4_**	10^−5^ s^−1^	**k_cat_/K_M_**	1.92 × 10^6^ M^−1^s^−1^
		**Initial thermodynamic parameters**	
		**K_eqtot_**	2.0 × 10^7^
		**X_tot_/RT**	16.81
		$\frac{Dissipation}{RT}$	Initial value (this paper)
		P	2.55 × 10^3^ s^−1^

**Table 6:** Initial values of microscopic rate constants from experimental and estimated data [27,28] for GI, to which we added our calculations of other initial kinetic and thermodynamic parameters in the case of the *Streptomyces murinus*-catalyzed conversion of glucose (substrate) to fructose (product) at 65 °C.

Rate Constants	Observed Values [27]	Calculated Values [28]	Other Relevant Parameters	Initial Values ([28] and This Paper)
k_1_*	3.8 M^−1^min^−1^	0.063 M^−1^s^−1^	**[S]**	2.0 M
**k_2_**	1.23 min^−1^	0.021 s^−1^	**[P]**	0.2 M
**k_3_**	1.75 min^−1^	0.029 s^−1^	**[E]**	0.01 M
k_4_*	4.9 M^−1^min^−1^	0.082 M^−1^s^−1^	**k_cat_**	0.029 s^−1^
k_1_ = k_1_* · [S]		0.126 s^−1^	**K_M_**	0.794 M
k_4_ = k_4_* · [P]		0.0164 s^−1^	**k_cat_/K_M_**	0.0365 M^−1^s^−1^
			**K_eqtot_**	10.61
			**X_tot_/RT**	2.36
			$\frac{Dissipation}{RT}$	Initial value (this paper)
			P	0.0406 s^−1^

## Data Availability

Fifteen FORTRAN source codes are available in the Supplementary Materials for reproducing all results where such codes are mentioned. Seventeen NetLogo codes are also free to download from the Supplementary Materials. The NetLogo codes enable the reproduction of similar figures to the reported figures where such codes are mentioned in the main text and Supplementary Materials.

## Supplementary Materials

The following supporting information can be downloaded at: https://www.mdpi.com/article/10.3390/e26020151/s1.
