# Supplementary material for: Theoretical Improvements in Enzyme Efficiency Associated with Noisy Rate Constants and Increased Dissipation
Source: Entropy (Basel). 2024 Feb 9;26(2):151. doi: 10.3390/e26020151 (PMC10888251; doi:10.3390/e26020151)
Supplement: Supplementary file 1 [file entropy-26-00151-s001.zip › Supplementary Materials final version.pdf]

Supplementary Materials for Theoretical Improvements in Enzyme Efficiency Associated with Noisy Rate Constants and Increased Dissipation

Davor Juretić[1,2,] and Željana Bonačić Lošić[2]*

[1] Mediterranean Institute for Life Sciences, Šetalište Ivana Meštrovića 45, 21000, Split, Croatia; davor.juretic@gmail.com, davor.juretic@medils.hr, juretic@pmfst.hr

[2] Faculty of Science, University of Split, Ruđera Boškovića 33, 21000, Split, Croatia; agicz@pmfst.hr

[*]Correspondence: davor.juretic@gmail.com

This file includes supplementary information:

Figures S1 to S20 with their legends and Table S1

Supplementary Text:
  Section S1. Linear Specificity-Dissipation Relationships
  Section S2. Flux-Turnover Relationships

Links for free download of our 15 FORTRAN and 17 NetLogo source codes:

- Simulation-S1-TPI-FORTRAN-for-FigS1-and-Fig2-final.for
- Simulation-S2-TPI-FORTRAN-for-Fig3-final.for
- Simulation-S3-TPI-FORTRAN-for-FigS2-final.for
- Simulation-S4-TPI-FORTRAN-f90-for-Fig4.f90
- Simulation-S5-TPI-FORTRAN-for-FigS4-final.for
- Simulation-S6-TPI-FORTRAN-for-Fig6-final.for
- Simulation-S7-TPI-FORTRAN-for-Fig7-final.for
- Simulation-S8-TPI-NetLogo-for-Fig8-and-FigS5-final.nlogo
- Simulation-S9-TPI-NetLogo-for-Fig9-final.nlogo
- Simulation-S10-KSI-FORTRAN-for-Fig10-final.for
- Simulation-S11-KSI-FORTRAN-for-Fig11-final.for
- Simulation-S12-KSI-FORTRAN-for-FigS6-final.for
- Simulation-S13-KSI-NetLogo-for-Fig12-final.nlogo
- Simulation-S14-CAI-FORTRAN-for-FigS7-final.for
- Simulation-S15-CAI-NetLogo-for-FigS8-final.nlogo
- Simulation-S16-CAII-NetLogo-for-Fig14-final.nlogo
- Simulation-S17-CAIIT200H-NetLogo-for-FigS9-final.nlogo
- Simulation-S18-PC1-NetLogo-for-FigS10-final.nlogo
- Simulation-S19-PC1-NetLogo-for-FigS11-final.nlogo
- Simulation-S20-RTEM-NetLogo-for-FigS12-final.nlogo
- Simulation-S21-RTEM-NetLogo-for-Fig16-final.nlogo
- Simulation-S22-Lac-1-NetLogo-for-FigS13-final.nlogo
- Simulation-S23-Lac-1-NetLogo-for-FigS14-final.nlogo
- Simulation-S24-Lac-1-NetLogo-for-FigS15-final.nlogo
- Simulation-S25-Lac-1-NetLogo-for-FigS16-final.nlogo
- Simulation-S26-beta-GAL-NetLogo-for-FigS17-final.nlogo
- Simulation-S27-beta-GAL-NetLogo-for-Fig18-and-Fig19-final.nlogo
- Simulation-S28-beta-GAL-FORTRAN-for-FigS18-final.for

Simulation-S29-beta-GAL-FORTRAN-for-Fig20-final.for
Simulation-S30-GI-NetLogo-for-FigS19-final.nlogo
Simulation-S31-GI-FORTRAN-for-FigS20-final.for
Simulation-S32-GI-FORTRAN-for-Fig21-final.for

The compressed codes are free to download from Source-codes-enzymes-corrected.zip

References

Figures S1-S20 and Table S1

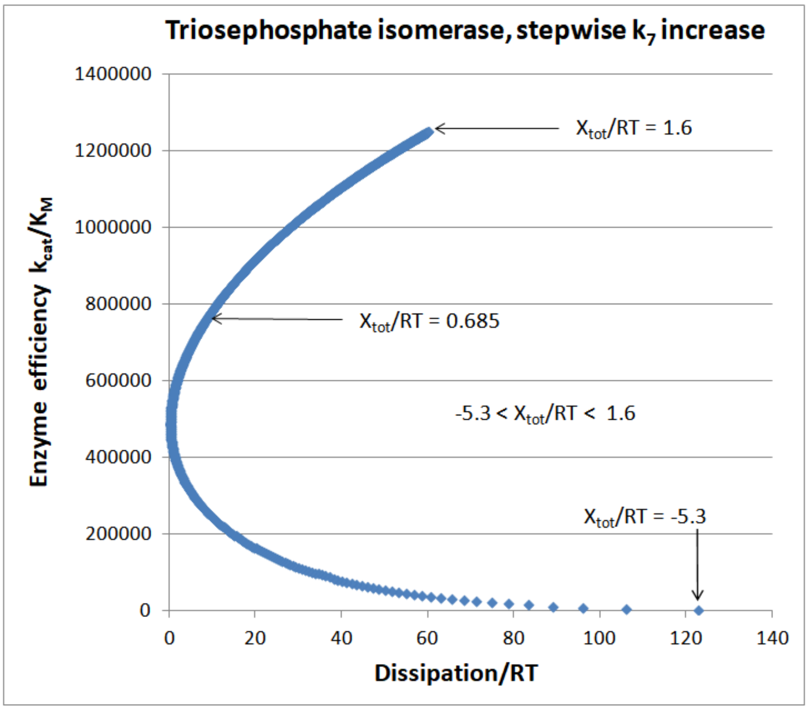

Figure S1. The catalytic efficiency dependence on dissipation after stepwise increase of the last forward rate constant k_7. The k_7 jumped 10.0 units in each of the 1000 deterministic steps in our Simulation-S1-TPI-FORTRAN software. There was no positive force requirement. Other conditions were the same as described in the legend of Figure 2 from the main text.

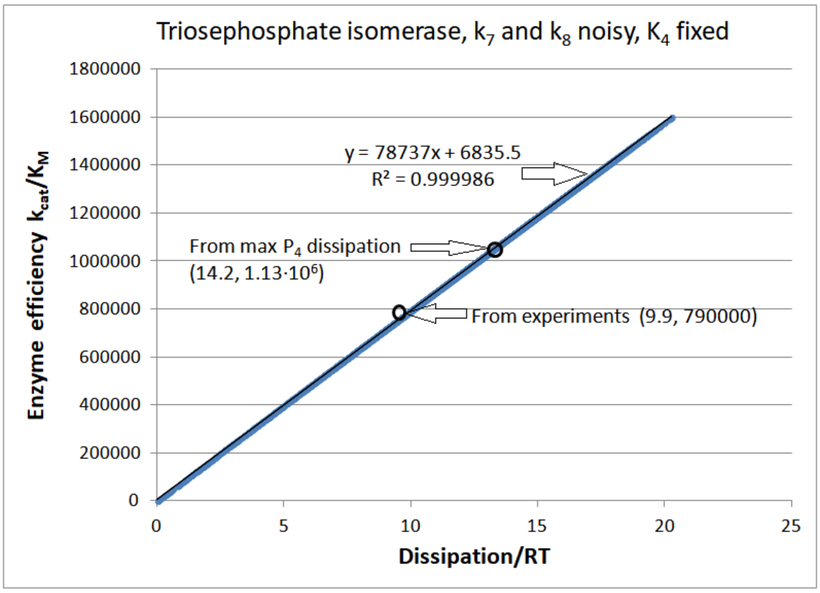

Figure S2. The catalytic efficiency dependence on dissipation for nondeterministic jumps between steady states when forward rate constant k_7 = 4000 s^{-1} is multiplied with the Box-

Muller transform for the random normal noise with the shift +2 (see Methods). The k_8 variation follows from the requirement $k_8=k_7/156.25$ ($K_4=156.25$), that is, from the no-change restriction for the calculated K_4 value from the observed data. The simulation software Simulation-S3-TPI-FORTRAN used to prepare this figure also required fixed K_1, K_2, K_3, and all other parameters at their initial values (see Table 1) for all 10000 computational steps. Highlighted points are for the dissipation and efficiency values calculated from the experimental data and the modest improvement achieved after the requirement that partial entropy production P_4 in the rate-limiting product release step is maximal (see Fig. 6 from the main text).

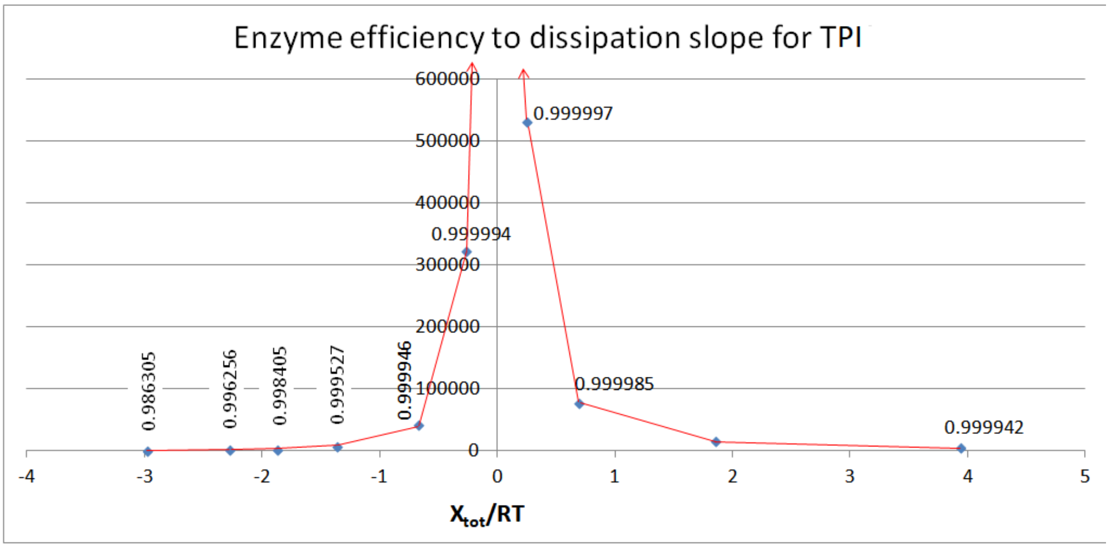

Figure S3. The figure is constructed after calculating linear fit and efficiency to the dissipation slope for 10 different X/RT values from -2.980 to 3.928 (corresponding to the total K_{eq} range from 4 to 4000). The physiological range for X_{tot}/RT values, when the TPI enzyme works in the cellular environment, is likely to be even more restricted to low and mainly positive X values. See the legends of Figures S2 and 5 for details about constructing the 10 software codes in the FORTRAN language used to create Figure S3.

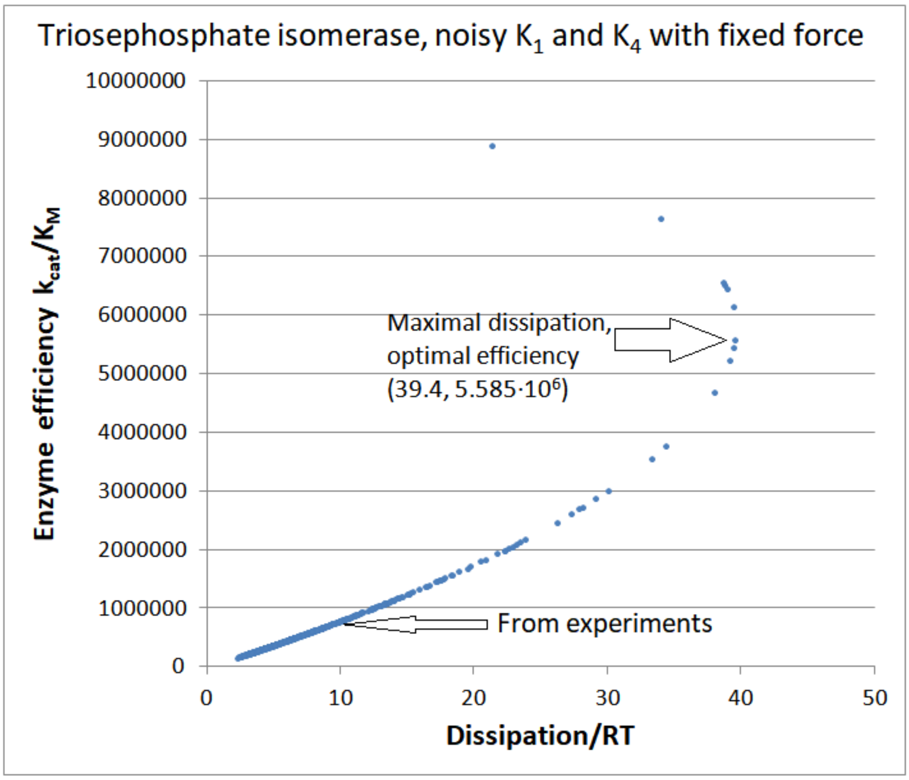

Figure S4. Enzyme efficiency k_{cat}/K_M as a function of dissipation/RT for noisy TPI kinetics with variations in K_1 and K_4 equilibrium constants and fixed overall force X_{tot}/RT. Each of the 2000 points in this figure corresponds to a randomly established steady state when K_4 is multiplied with the normal noise function with a +2 shift. The simulation software Simulation-S5-TPI-FORTRAN found a unique steady state with optimal enzyme efficiency and maximal total entropy production, such that the optimal efficiency (higher arrow y coordinate) is considerably higher than the calculated catalytic efficiency using experimental data (lower arrow y coordinate from this Figure and Figure S2).

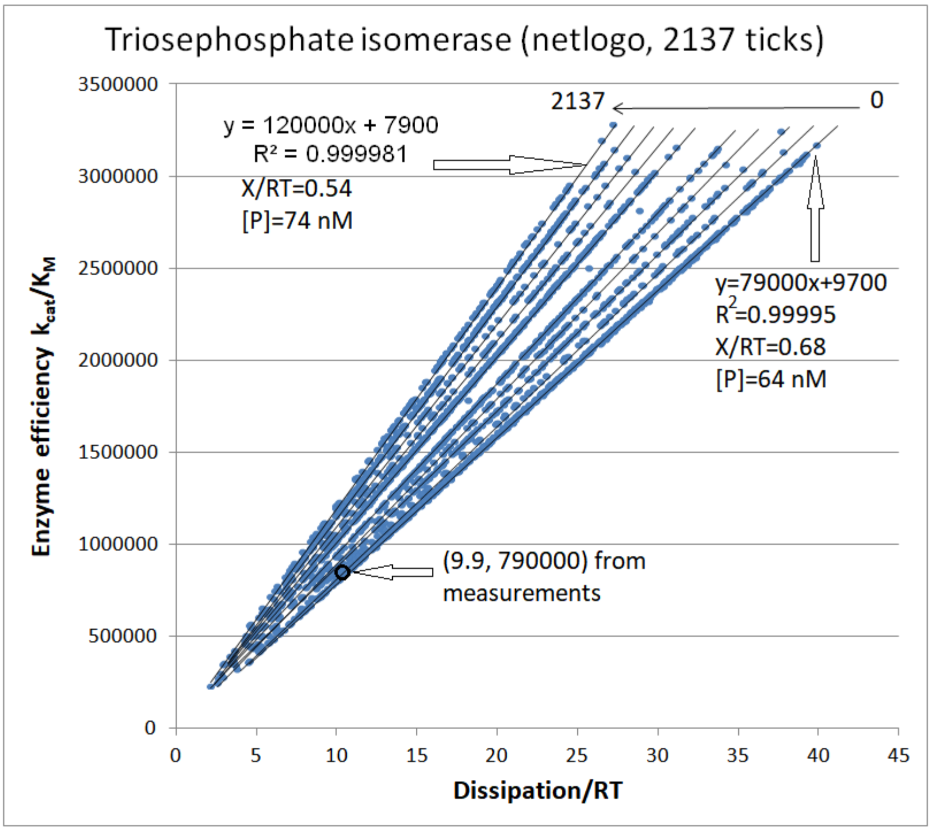

Figure S5. The more extended simulation run using the same software and identical conditions as in Figure 8. The program Simulation-S8-TPI-NetLogo was stopped at 2137 ticks when initial concentrations $[S]_{init}$ = 40 μM, $[P]_{init}$ = 0.064 μM, and $[enzyme]_{free-init}$ = 100 nM changed to $[S]_{final}$ = 39.902 μM, $[P]_{final}$ = 0.074 μM, $[enzyme]_{free-final}$ = 12 nM. Final concentrations of enzyme-ligand complexes were [ES] = 19 nM, [EZ] = 35 nM, and [EP] = 34 nM. Overall force in the RT units (X/RT) dropped from the initial 0.685 to the final 0.538.

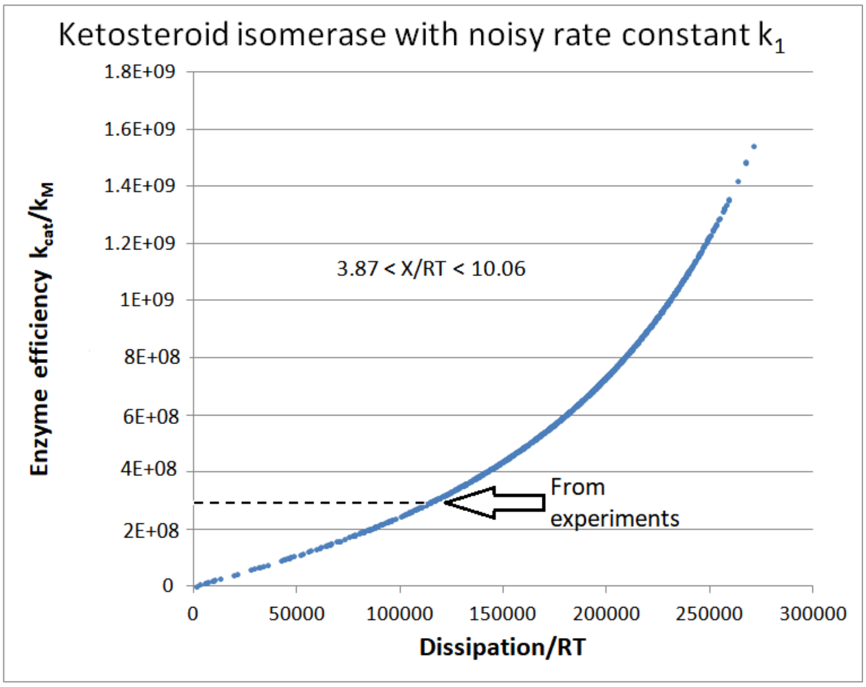

Figure S6. When dissipation increases, enzyme efficiency increases even faster in the presence of noisy associations between substrates and enzymes. We multiplied k_1 with the normal noise function, which included the shift = +2 (see Methods). The Simulation-S12-KSI-FORTRAN runs through a thousand steps. Each step involved a random change in the rate constant k_1 without any other changes from the initial calculated values (Table 2, second column). Thus, noisy X_{tot}/RT values in the range from 3.87 to 10.06 resulted from noisy K_1 values in the range from 0.01 to 4.93.

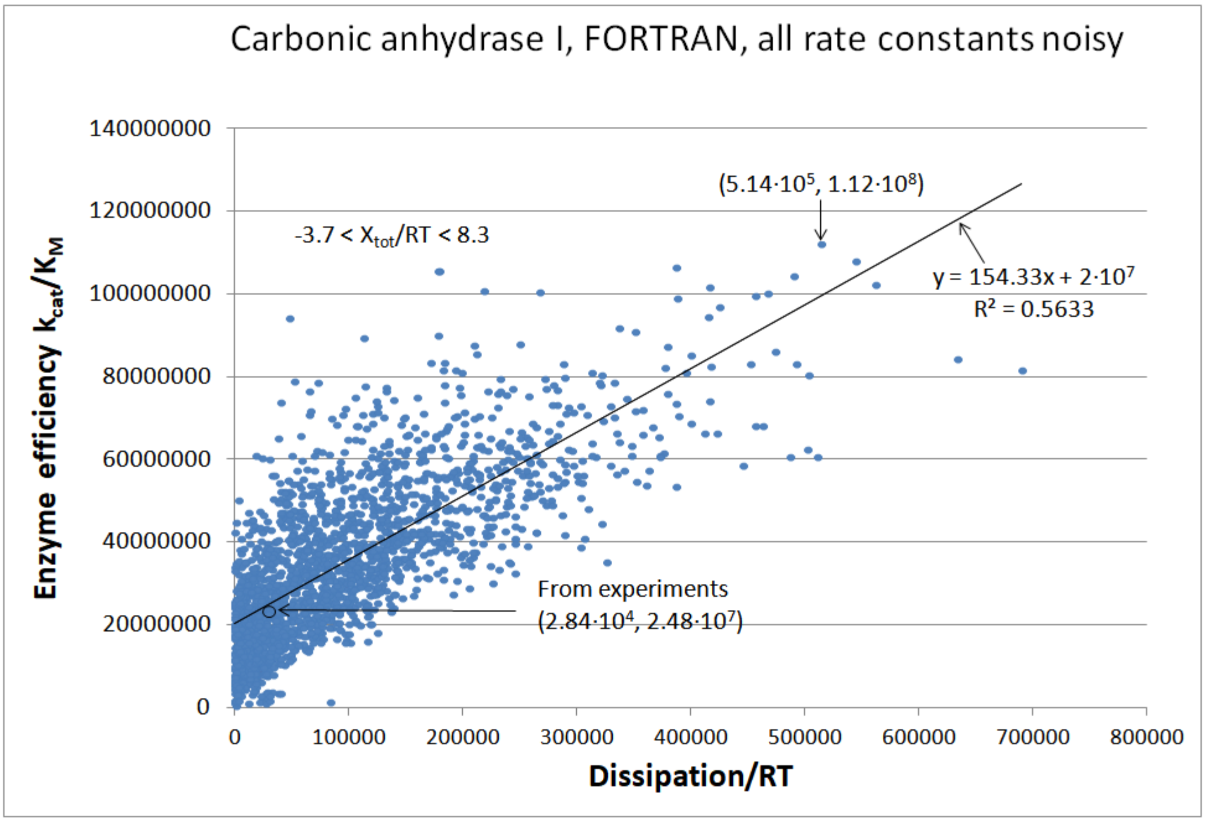

Figure S7. The simulation of the relationship between catalytic efficiency and overall dissipation for carbonic anhydrase I, when each of eight rate constants k_i is multiplied with independently called normal noise function gi without shift (see Methods, eq (32)). Only positive g_i values were allowed while the Simulation-S14-CAI-FORTRAN ran through two thousand steps. There was no change in the substrate, product, and buffer concentration from their initial values (Table 3).

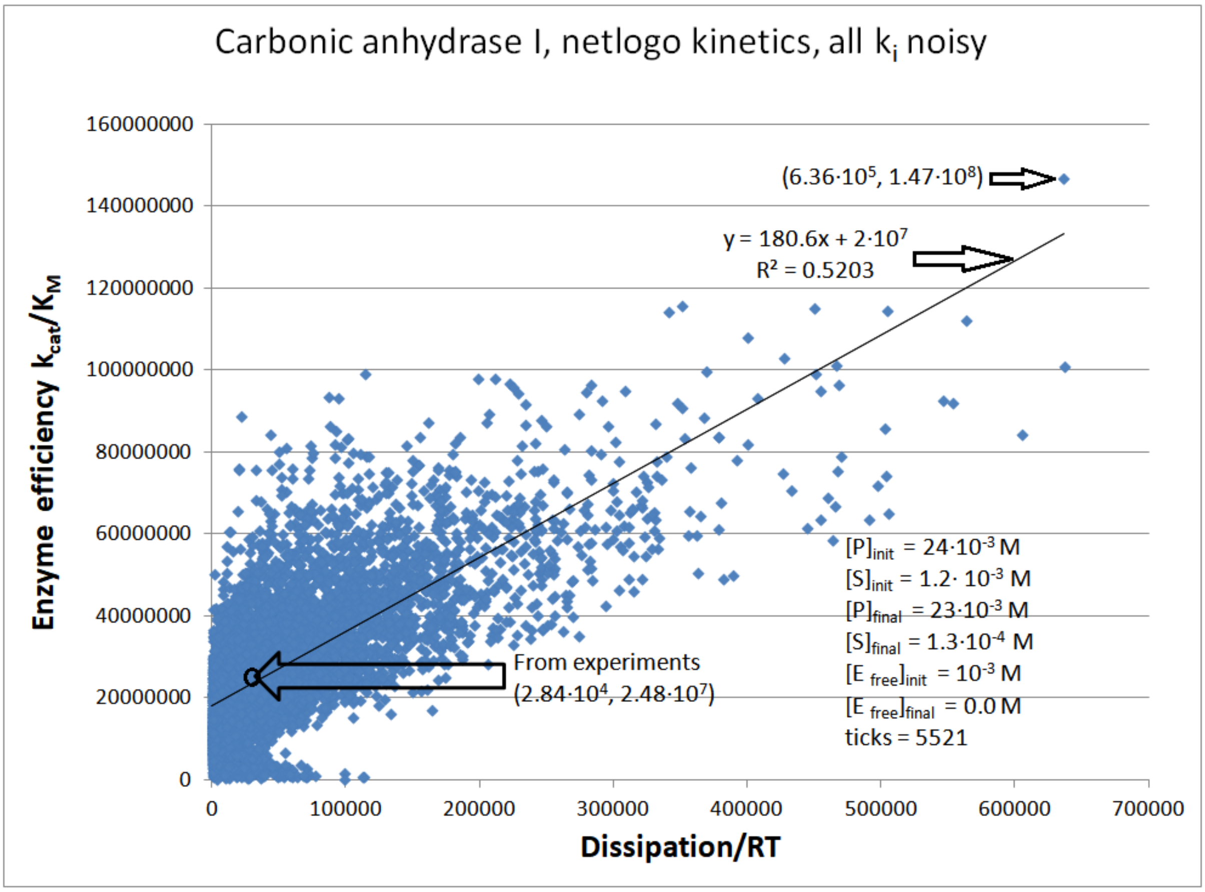

Figure S8. The NetLogo simulation of the relationship between catalytic efficiency and overall dissipation for carbonic anhydrase I, when each of eight rate constants k_i is multiplied with independently introduced normal noise function g_i without shift (see Methods, Eq. (32)). The best efficiency value of $1.47 \cdot 10^8$ $M^{-1}s^{-1}$ resulted in the 2774th tick for $X_{tot}/RT = 6.5$. The overall force in the RT units ranged from -5.0 to 8.5, but there was no significant force decrease with the time passage (ticks). From the initial 100 μM free enzyme concentration, the conversion during 5521 ticks ended up with less than 1 μM free enzyme concentration, [ES] = 1 μM, [EX] = 45 μM, and [EZ] = 54 μM. The Michaelis-Menten time dependence pattern is seen for the ES complex concentration (fast rise followed by plateauing and slow decrease). During the Simulation-S15-CAI-NetLogo run, there was little change in the substrate (CO_2) and product (HCO_3^-) concentration.

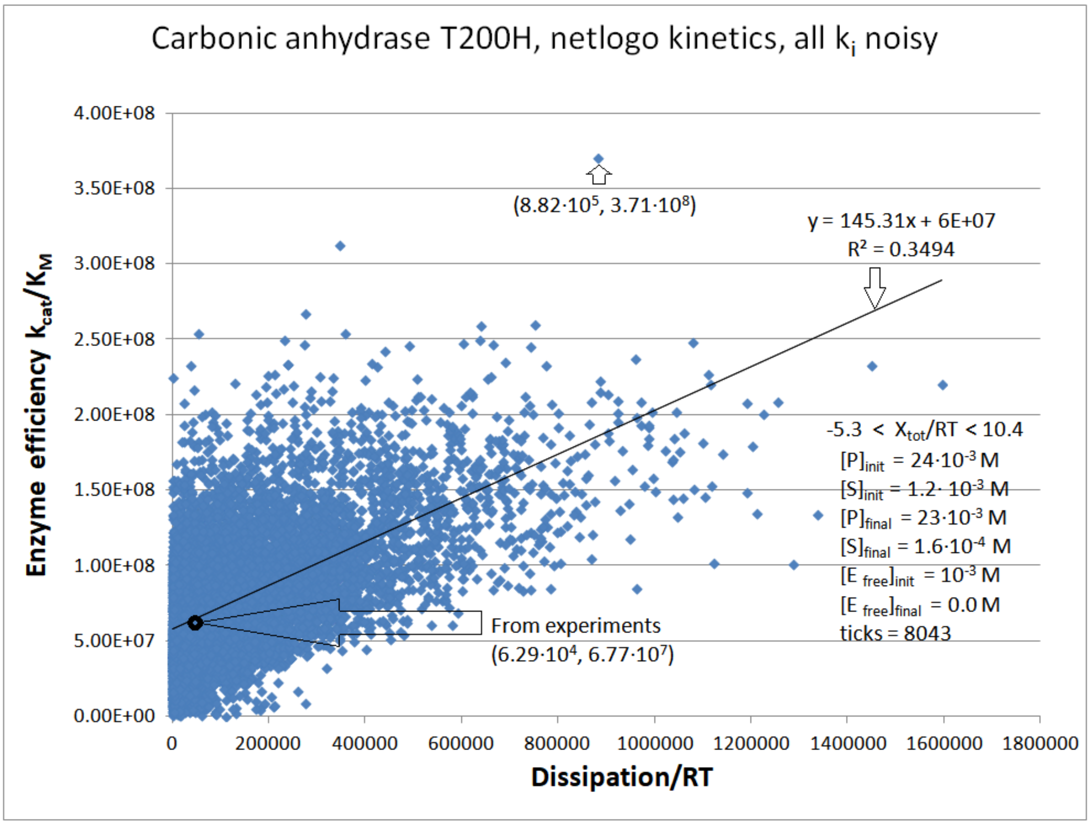

Figure S9. The catalytic efficiency dependence on dissipation for the T200H mutant of carbonic anhydrase II. We used our software Simulation-S17-CAIIT200H-Netlogo to construct Figure S9.

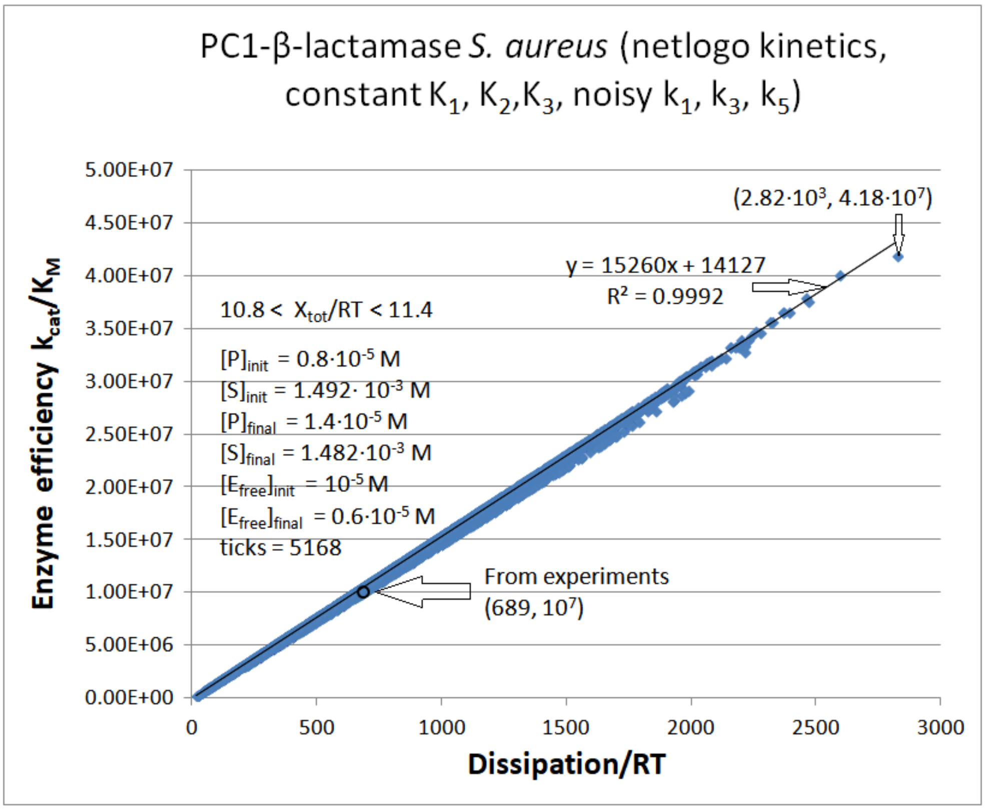

Figure S10. The catalytic efficiency dependence on dissipation when all forward rate constants are noisy but all equilibrium constants $K_1^* = k_1^*/k_2$, $K_2 = k_3/k_4$, and $K_3^* = k_5/k_6^*$ maintain their observed values [1] for the PC1 β-lactamase. As expected for strong and constant positive force, there is close to perfect efficiency-dissipation proportionality despite introduced and inherent noise in encounters among the enzyme and ligand conformers in our simulation when identical normal noise is introduced in k_1, k_2, and k_3. The force decreased from 11.4 to 10.8 as the NetLogo simulation proceeded through 5168 ticks. The best point with the highest dissipation ($2.82 \cdot 10^3$ s^{-1}) and the highest enzyme efficiency ($4.18 \cdot 10^7$ $M^{-1}s^{-1}$) occurred when an overall force of 11.3 was very similar to the initial force (11.4). We used our Simulation-S18-PC1-NetLogo software to construct Figure S10.

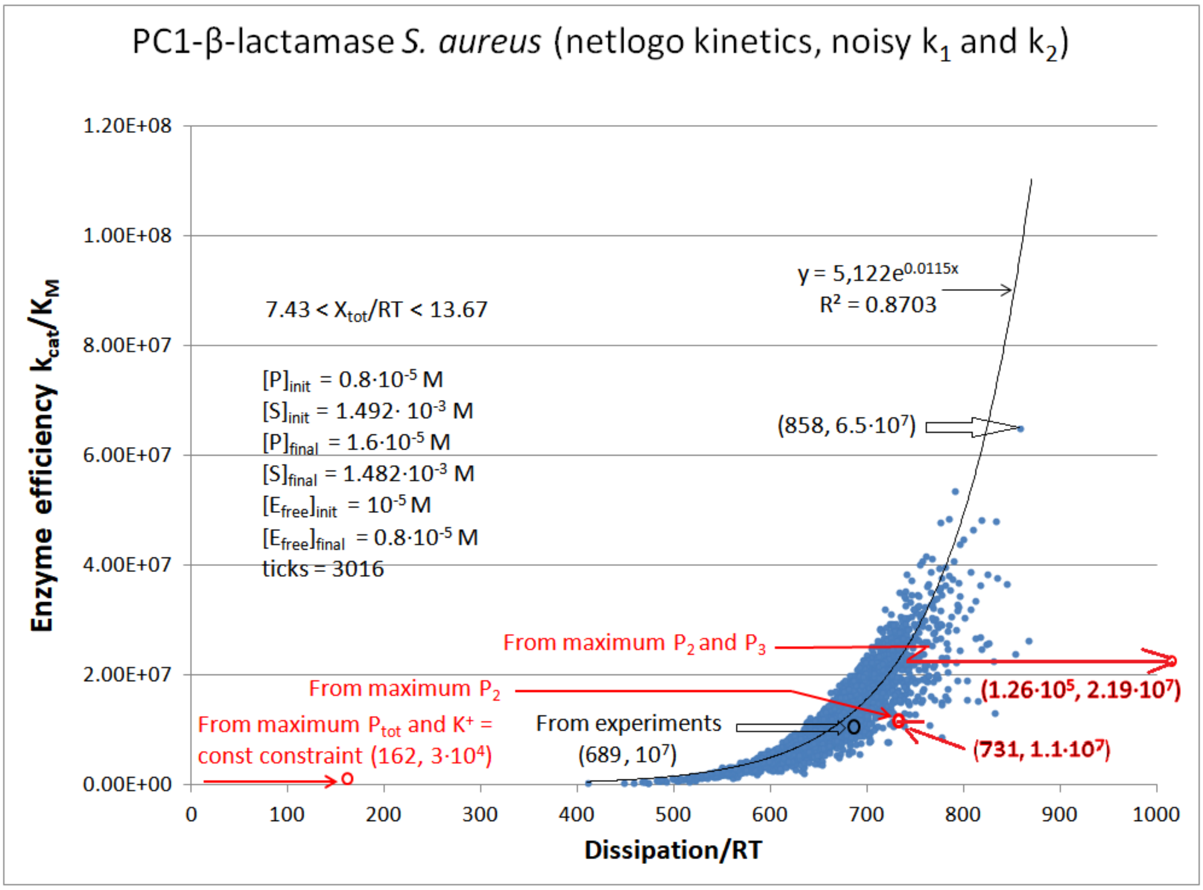

Figure S11. The catalytic efficiency dependence on dissipation when noise is introduced twice in the NetLogo simulation - in the forward rate constant k_1 and the backward rate constant k_2. Variations only in the substrate-enzyme association-dissociation produced close to an exponential increase in the enzyme efficiency after dissipation increased. The best efficiency value of $6.5 \cdot 10^7$ $M^{-1}s^{-1}$ is now close to the lower-end diffusion-limit range (10^8 $M^{-1}s^{-1}$). There was no change in the catalytic constant from observed k_{cat} = 60.8 $^1s^{-1}$ during the 3016 simulation ticks because forward $k_{cat\ (S\to P)} = k_3 \cdot k_5/(k_3+k_4+k_5)$ does not depend on the rate constants k_1 and k_2 for the first catalytic step. Descriptions and arrows in red depict the optimization results from [1,2]. The best efficiency value of $6.5 \cdot 10^7$ $M^{-1}s^{-1}$ corresponds to 6.1-fold higher k_1 and 3.5-fold smaller k_2, which increased 21-fold the first equilibrium constant

$K_1 = k_1/k_2$ from observed 167.5 to the optimal value of 3582.3, with the concomitant increase in the overall force X_{tot}/RT from the referent value of 11.4 to the optimal value of 14.1. We used our Simulation-S19-PC1-NetLogo software to construct Figure S11.

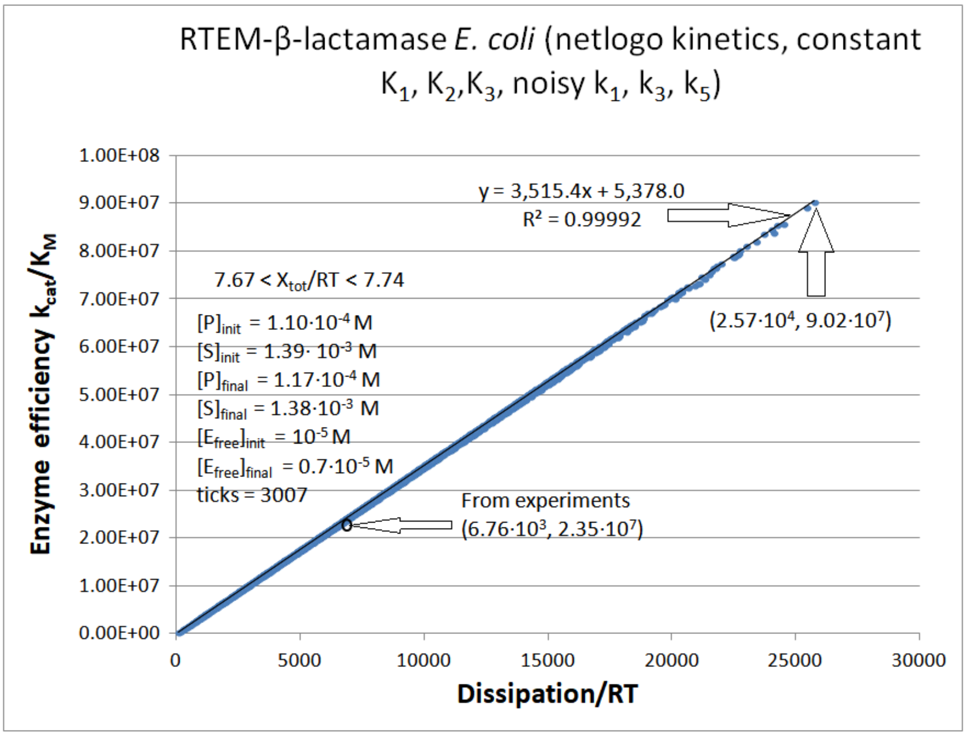

Figure S12. The catalytic efficiency dependence on dissipation when all forward rate constants are noisy but all equilibrium constants $K_1^* = k_1^*/k_2$, $K_2 = k_3/k_4$, and $K_3^* = k_5/k_6^*$ maintain their observed values [1] for the RTEM β-lactamase (see Table 4). We used our Simulation-S20-RTEM-NetLogo software to construct Figure S12.

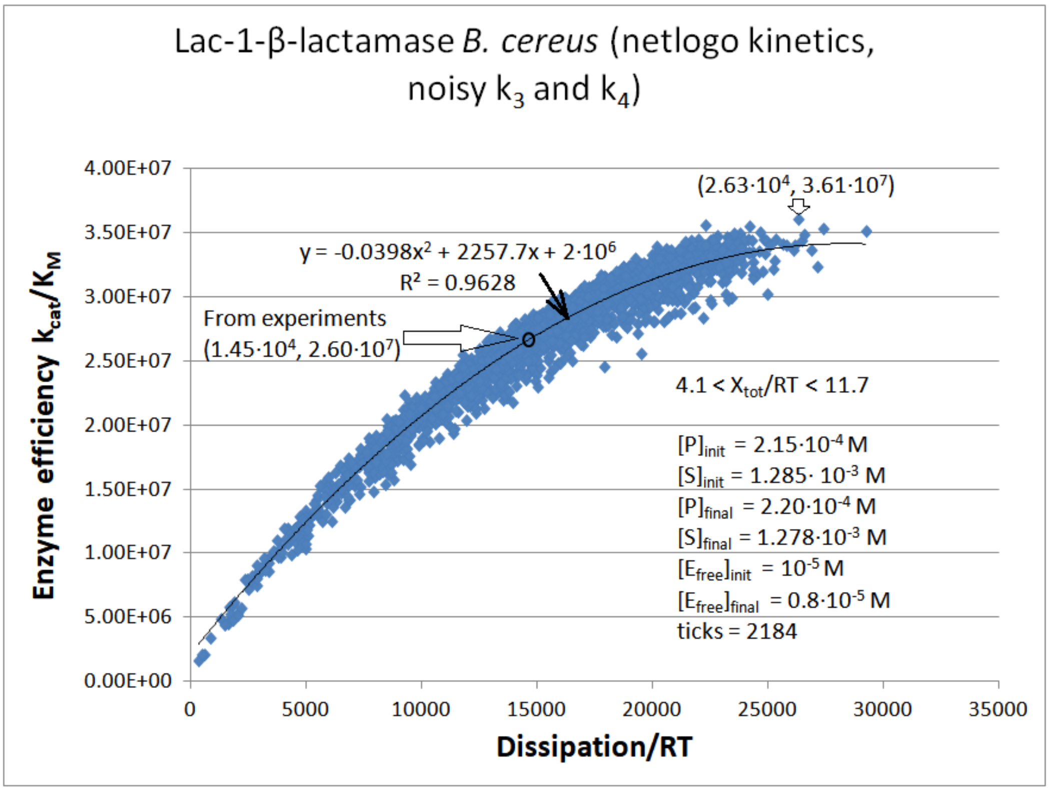

Figure S13. The catalytic efficiency dependence on dissipation when noise is introduced twice in the NetLogo simulation for Lac-1 β-lactamase kinetics - in the forward rate constant k_3 and the backward rate constant k_4. We used our Simulation-S22-Lac-1-NetLogo software to construct Figure S12.

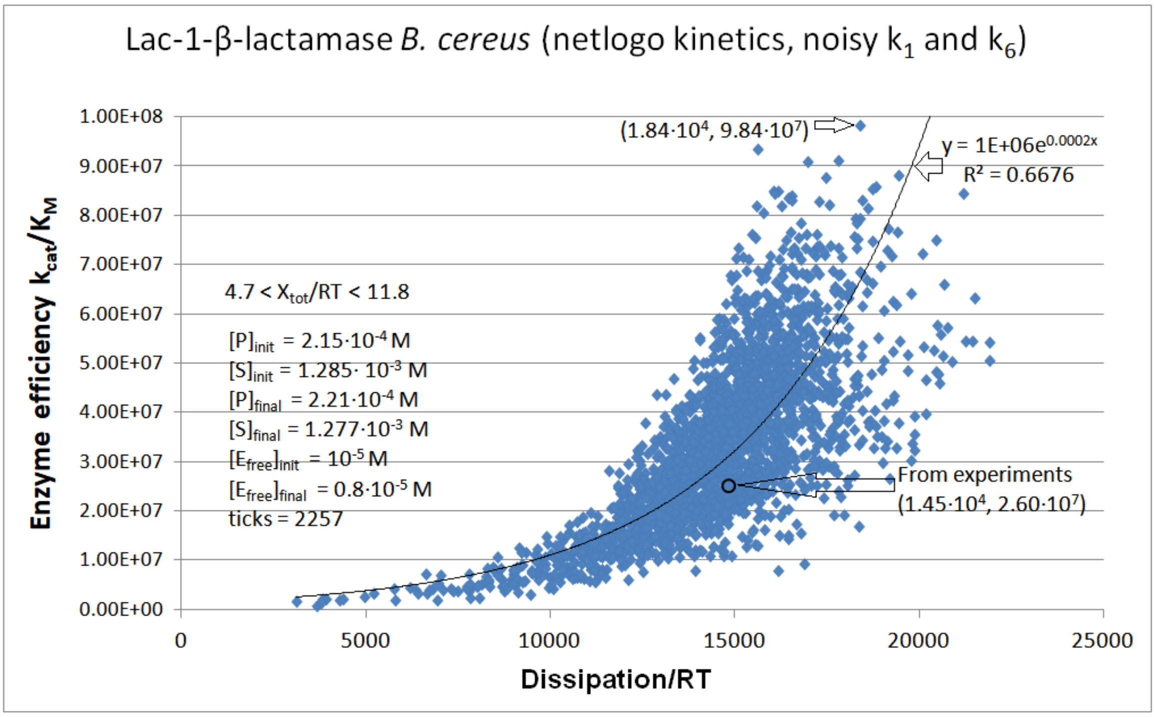

Figure S14. The catalytic efficiency dependence on dissipation when noise is introduced twice in the NetLogo simulation for Lac-1 β-lactamase kinetics - in the forward rate constant k_1 and the backward rate constant k_6. We used our Simulation-S23-Lac-1-NetLogo software to construct Figure S14.

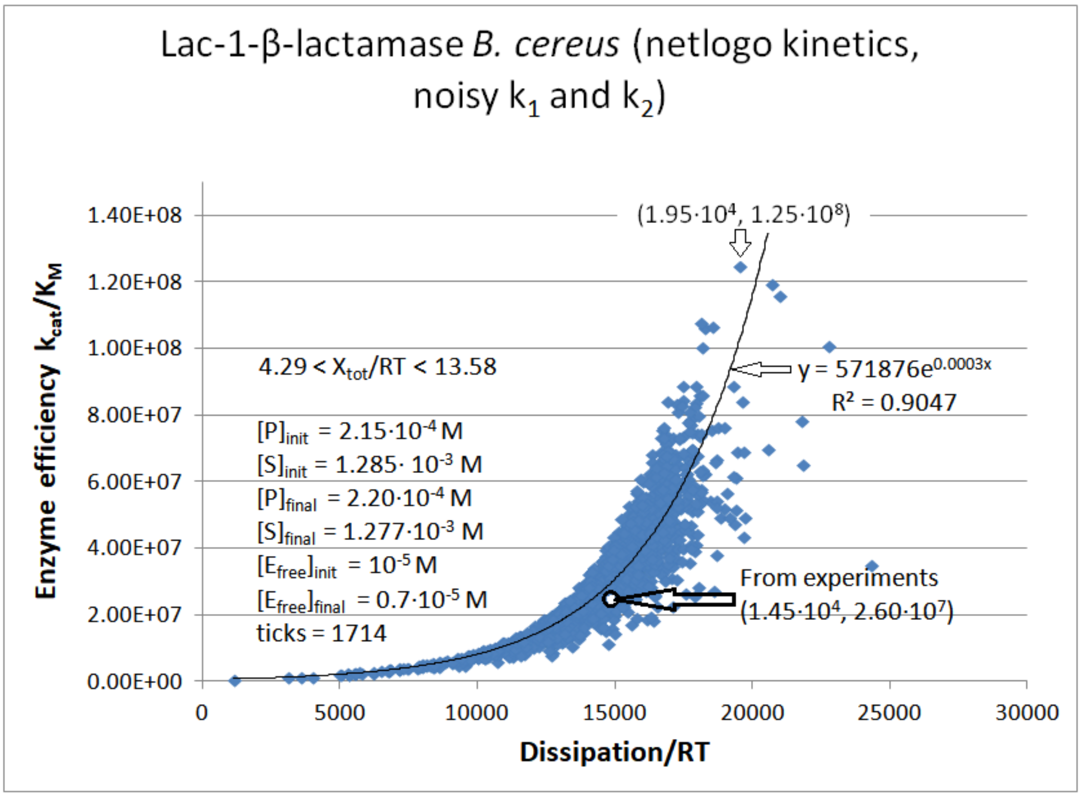

Figure S15. The catalytic efficiency dependence on dissipation when noise is introduced twice in the NetLogo simulation for Lac-1 β-lactamase kinetics - in the forward rate constant k_1 and the backward rate constant k_2. We used our Simulation-S24-Lac-1-NetLogo software to construct Figure S15.

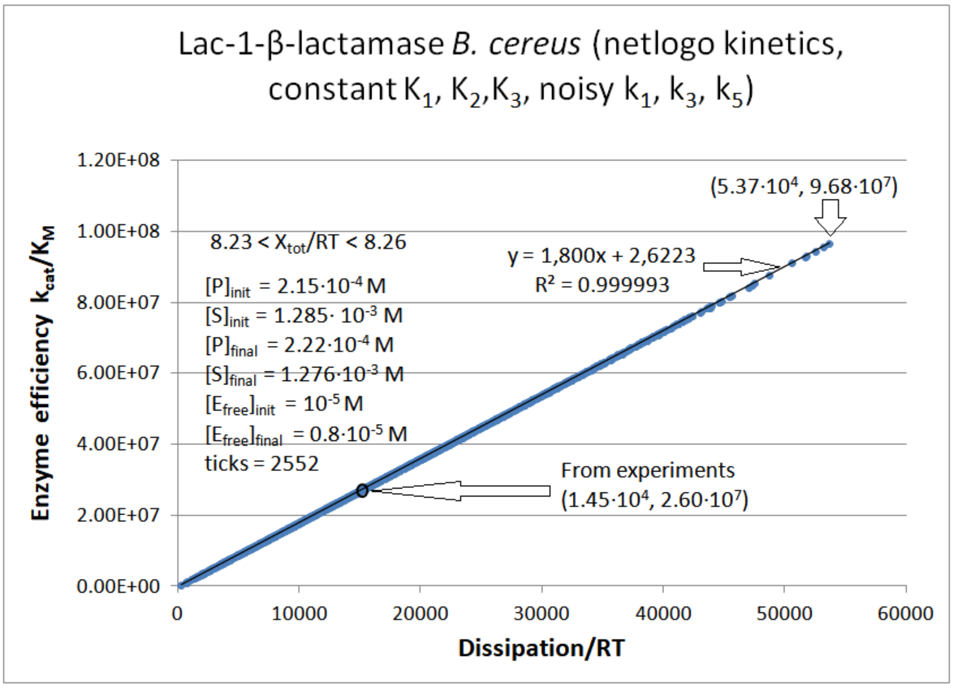

Figure S16. The catalytic efficiency dependence on dissipation when all forward rate constants are noisy but all equilibrium constants $K_1^* = k_1^*/k_2$, $K_2 = k_3/k_4$, and $K_3^* = k_5/k_6^*$ maintain their observed values [1] for the Lac-1 β-lactamase. The coordinates for the best point are ($5.37{\cdot}10^4$ s^{-1}, $9.68{\cdot}10^7$ $M^{-1}s^{-1}$). The corresponding optimal values for the rate constants are 3.7 times higher than the initial values (Table 4). We used our Simulation-S25-Lac-1-NetLogo software to construct Figure S16.

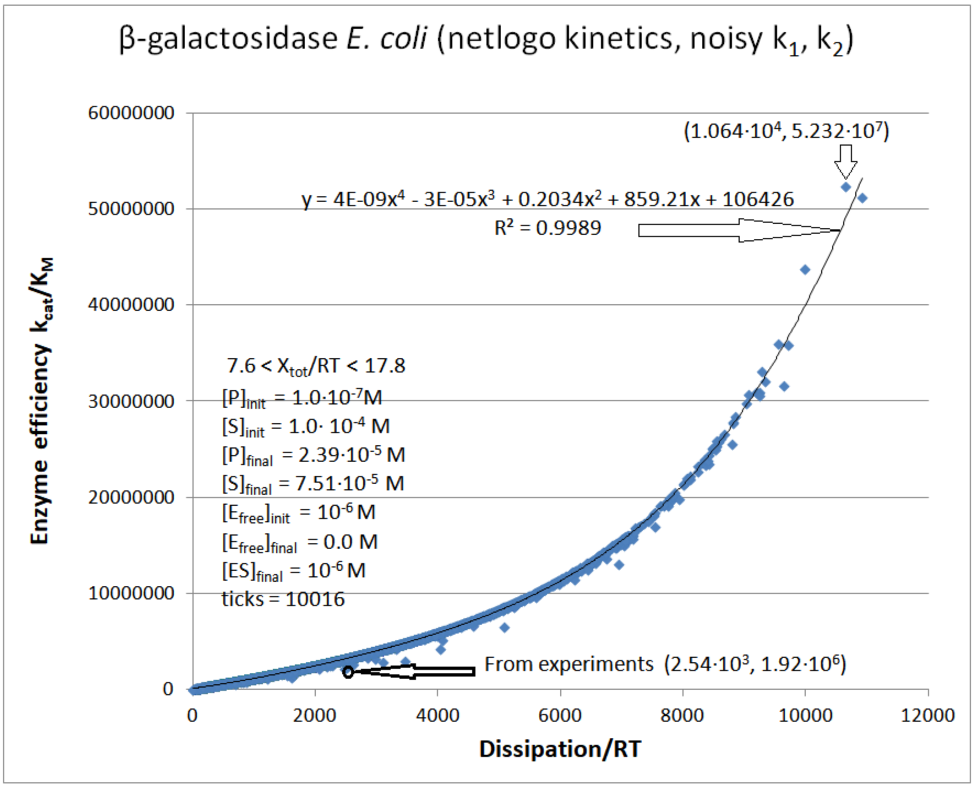

Figure S17. The catalytic efficiency dependence on dissipation when normal noise is introduced twice, in the forward rate constant k_1 as $k_1 \cdot g_1$, and, independently in the corresponding backward rate constant k_2 as $k_2 \cdot g_2$ for the first catalytic step (E+S↔ES) in the case of β-galactosidase kinetics (see Methods). The initial concentration of enzymes was higher than the initial concentration of products, but that quickly changed after less than 100 ticks when product concentration increased about 24 times. The subscript "free" means the enzyme is free from substrate or product. We used our Simulation-S26-beta-GAL-NetLogo software to construct Figure S17.

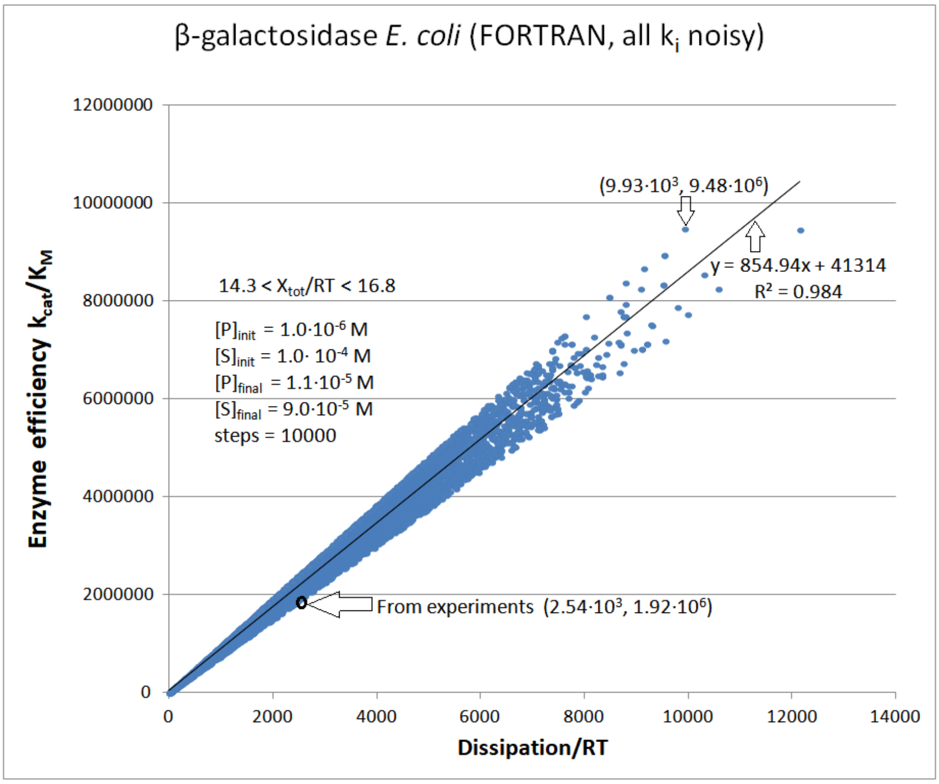

Figure S18. The simulation of enzyme efficiency dependence on dissipation in the presence of noisy rate constants and small changes in the substrate and product concentrations. We used our Simulation-S28-beta-GAL-FORTRAN software to get the kinetic and thermodynamic parameters we needed to construct Figure S18. We called Gaussian noise once (see Eq. (32) in Methods). Thus, straight-line efficiency-dissipation dependence exhibits slight changes only due to concentration changes despite introduced noise in all rate constants.

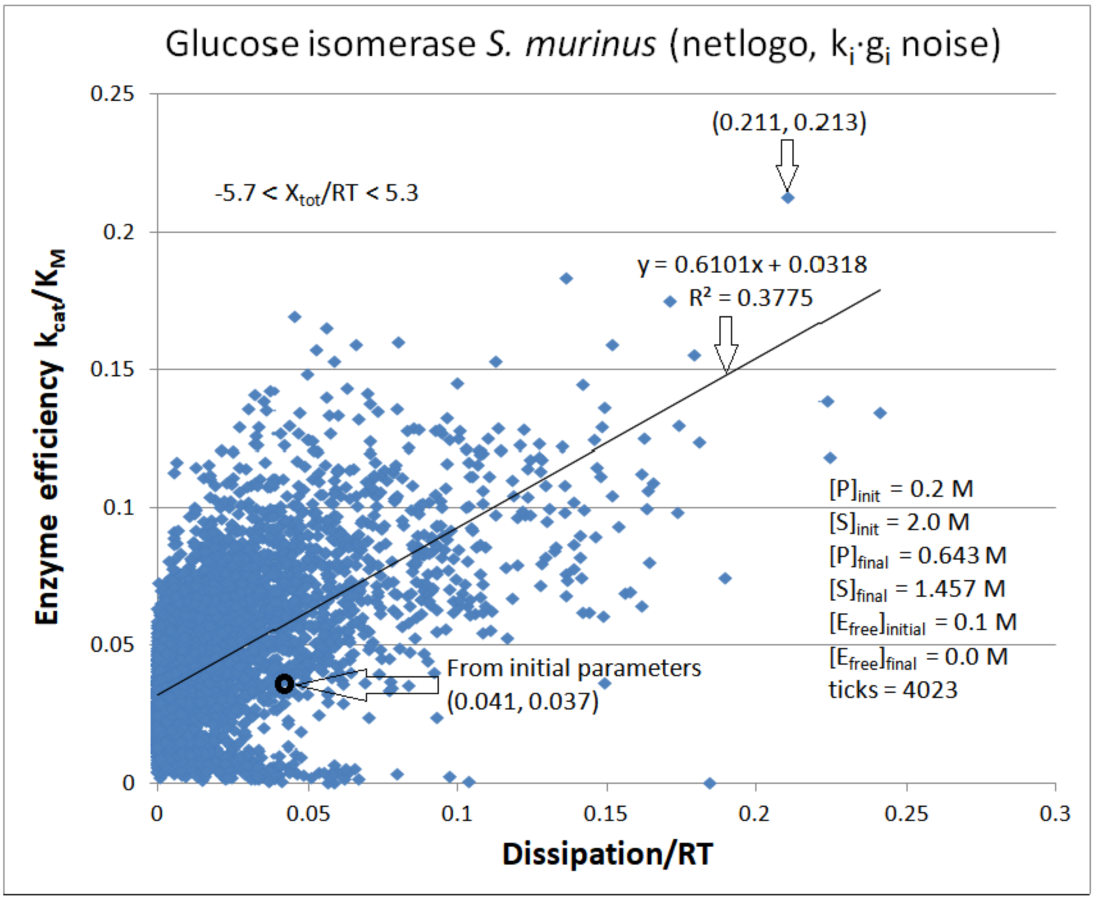

Figure S19. The catalytic efficiency dependence on dissipation in the case of glucose isomerase kinetics when normal noise is introduced independently in all rate constants k_i. The assumed temperature was 65 $^{\circ}$C. Data for referent kinetic constants (in moles) are from Table 6, while initial substrate $[S]_{initial}$ and product $[P]_{initial}$ concentrations of, respectively, 2 molar and 0.2 molar, are chosen so that the enzyme works mainly in the forward direction. The simulation with 100 enzymes corresponds to a 100 mM initial concentration of free enzymes. That number quickly drops to zero $[E]_{free}$ concentration because all free enzymes are converted into the 100 mM [ES] complex concentration after only 29 ticks. Due to discrete NetLogo simulation steps, $[E]_{free}$ = 0 can be any concentration smaller than 0.5 mM. The maximal value of catalytic efficiency of 0.213 $M^{-1}s^{-1}$ is reached in the 1715th step (tick). That efficiency is 5.8 times higher than the value calculated from initial parameters in the absence of noise. We used our Simulation-S30-GI-NetLogo software to get the kinetic and thermodynamic parameters we needed to construct Figure S19.

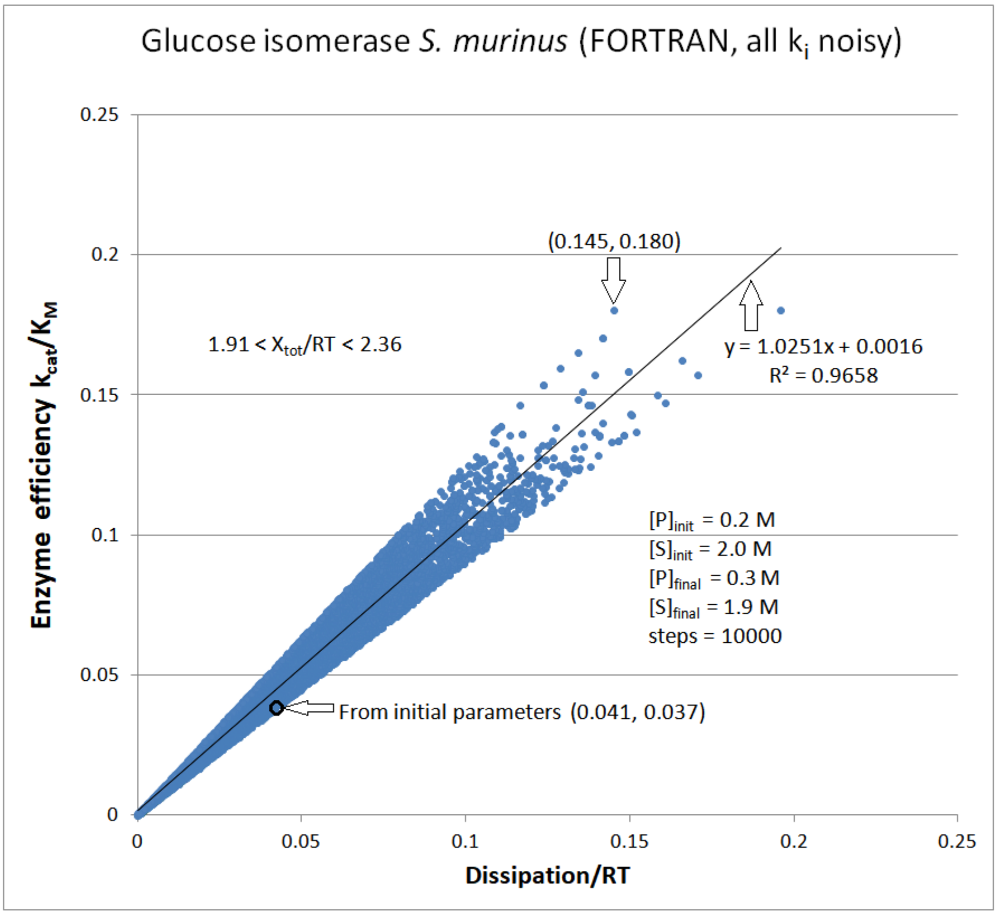

Figure S20. The catalytic efficiency dependence on dissipation when rate constants k_i (i = 1, 2, 3, 4) are noisy and small stepwise changes are allowed for the substrate and product concentrations. Box-Muller transform g_1 with shift +1 (see Methods) was called only once and multiplied with each k_i. The main loop from our Simulation-S31-GI-FORTRAN software went through the 10000 steps. We used its output to construct Figure S20.

Table S1. Kinetic and thermodynamic parameters for the NetLogo runs for noisy k_1, k_2, k_3, and k_4. The first run corresponds to Figure 18. The second run corresponds to Figure 19. The "Best B" notation is for the parameters in the quasi-steady state with the highest catalytic efficiency. The "Last L" notation is for the steady state when we stopped the program after concentrations did not change for many ticks.

Run # Best	Total ticks	B or L tick*	$[S]_{free}$ (M)	$[P]_{free}$ (M)	k_1 (s^{-1})	k_2 (s^{-1})	k_3 (s^{-1})	k_4 (s^{-1})	X_{tot}/RT	K_M (M)	Dissip/RT (s^{-1})**	k_{cat}/K_M ($M^{-1}s^{-1}$)
1	12049	390	$9.996\cdot10^{-5}$	$1.3\cdot10^{-7}$	14109	467	726	$2.25\cdot10^{-7}$	25.3	$8.45\cdot10^{-6}$	16948	$8.59\cdot10^{7}$
2	8021	373	$9.996\cdot10^{-5}$	$1.3\cdot10^{-7}$	14325	251	784	$1.26\cdot10^{-6}$	24.3	$7.22\cdot10^{-6}$	17759	$1.09\cdot10^{8}$
Run # Last												
1	12049	12049	$9.530\cdot10^{-5}$	$3.80\cdot10^{-6}$	2843	$3.428\cdot10^{4}$	224	$1.04\cdot10^{-4}$	12.1	$1.16\cdot10^{-3}$	207<br>Av: 1310	$1.94\cdot10^{5}$
2	8021	8021	$9.564\cdot10^{-5}$	$3.46\cdot10^{-6}$	5084	4390	780	$8.32\cdot10^{-5}$	16.2	$9.73\cdot10^{-5}$	6268<br>Av: 2852	$8.02\cdot10^{6}$

* The last state, „L, “ is our subjective choice for ending the program run. It does not have any special meaning.

** An average (Av) dissipation/RT value is calculated only for the last 10 ticks.

Supplementary Text

Section S1. Linear Specificity-Dissipation Relationships

We examined the conditions for nearly perfect efficiency to dissipation proportionality. Examples are kinetics simulations for some enzymes, which illustrate the remarkably linear dependency of catalytic efficiency on overall entropy production (Figures 3,S2,5,6,8,S5,S10,S12,S16,S18,S20). For other enzymes we studied in this paper, such linear dependence was not explicitly shown, although we obtained it when identical assumptions were used. Since such proportionality can be the previously unmentioned hallmark of all enzymes exhibiting generalized Michaelis-Menten kinetics, it is worthwhile to analyze the assumptions and their mathematical consequences.

We first give the expressions for the dependence of efficiency on dissipation for two states

$$\frac{k_{cat}/K_M}{Dissipation} = \frac{1}{K-1}\frac{K_2+K+(K_1+K)\frac{k_3}{k_1}}{[S]\left[\frac{1}{K_1}+\frac{k_3}{k_1}\right]RTlnK} \quad \text{(S1)}$$

three states

$$\frac{k_{cat}/K_M}{Dissipation} = \frac{1}{K-1}\frac{(K_3+K_1K_3+K)+(K_1+K_1K_2+K)\frac{k_5}{k_1}+(K_2+K_1K_2+K)\frac{k_5}{k_3}}{[S]\left[\frac{k_5}{k_1}+\frac{1}{K_1}\left(\frac{k_5}{k_3}+\frac{1}{K_2}\right)\right]RTlnK} \quad \text{(S2)}$$

and four states

$$\frac{k_{cat}/K_M}{Dissipation} = \frac{K_1}{K-1}\frac{(K_2+K_2K_3+K_2K_3K_4+K)+(K_1+K_1K_2+K_1K_2K_3+K)\frac{k_3}{k_1}}{[S]\left[1+K_1\frac{k_3}{k_1}+\frac{1}{K_2}\frac{k_3}{k_5}\left(1+\frac{1}{K_3}\frac{k_5}{k_7}\right)\right]RTlnK}+$$

$$\frac{K_1}{K-1}\frac{(K_3+K_3K_4+K_1K_3K_4+K)\frac{k_3}{k_5}+(K_4+K_1K_4+K_1K_2K_4+K)\frac{k_3}{k_7}}{[S]\left[1+K_1\frac{k_3}{k_1}+\frac{1}{K_2}\frac{k_3}{k_5}\left(1+\frac{1}{K_3}\frac{k_5}{k_7}\right)\right]RTlnK}\ . \quad \text{(S3)}$$

The next task is to examine the conditions when these expressions for the efficiency to dissipation slope are less likely to change due to variations in the microscopic rate constants. When equilibrium constants for each catalytic step are kept fixed to their observed values, the change in slope can happen only in the case of variable temperature, variable concentrations, or variable ratios of rate constants belonging to different catalytic steps. For selected quasi-steady states, there should be no change in temperature, but the substrate and product concentration do change for an active enzyme if not fixed to their initial values. It all depends on how jumps between steady states are defined. When random numbers s_1 and s_2 are called only once in the Box-Muller transform (see Methods), the slope never changes for the constant concentration of ligands because the noise cancels in all ratios of rate constants k_i/k_j, where i and j do not have to be from the same catalytic step. There are slight changes in the slope after allowing for minor changes in the concentration of substrates, products, and enzyme-ligand complexes within the same assumption that identical noise is introduced into

one or more catalytic steps. Still, the slope only undergoes a limited increase due to the increase in product concentration and slight force decrease. An entirely different situation occurs when noise is introduced independently two or more times in one or more pairs of rate constants. The constraint of fixed or slightly variable equilibrium constants in catalytic steps is then abandoned, and the goodness of the straight line fit for the proportional dependence of enzyme efficiency on dissipation varies among enzymes but is still strongly affected if any other constraint is imposed.

Figure 8 from the main text illustrates the example of concentration changes causing jumps among specificity-dissipation lines (TPI enzyme, Simulation-S8-TPI-NetLogo software). We imposed the constraint of fixed equilibrium constants K_1^*, K_2, K_3, and K_4^* , where $K_1^* = K_1/[S]$, and $K_4^* = K_4[P]$. Due to slight changes in the substrate and product concentrations, the equilibrium constants K_1 and K_4 also underwent small changes.

Section S2. Flux-Turnover Relationships

We give the expressions for the dependence of turnover number k_{cat} on net flux for two states

$$\frac{k_{cat}}{J} = \frac{K_2+K+\frac{k_3}{k_1}(K_1+K)}{K-1} \tag{S4}$$

three states

$$\frac{k_{cat}}{J} = \frac{1}{K-1}\frac{(K_3+K_1K_3+K)+(K_1+K_1K_2+K)\frac{k_5}{k_1}+(K_2+K_1K_2+K)\frac{k_5}{k_3}}{1+\frac{1}{K_2}+\frac{k_5}{k_3}} \tag{S5}$$

and four states

$$\frac{k_{cat}}{J} = \frac{1}{K-1}\frac{(K_2+K_2K_3+K_2K_3K_4+K)+(K_1+K_1K_2+K_1K_2K_3+K)\frac{k_3}{k_1}}{1+\frac{k_3}{k_7}+\frac{k_3}{k_5}\left(1+\frac{1}{K_2}\right)\left(1+\frac{k_5}{K_3k_7}\right)} + \frac{1}{K-1}\frac{(K_3+K_3K_4+K_1K_3K_4+K)\frac{k_3}{k_5}+(K_4+K_1K_4+K_1K_2K_4+K)\frac{k_3}{k_7}}{1+\frac{k_3}{k_7}+\frac{k_3}{k_5}\left(1+\frac{1}{K_2}\right)\left(1+\frac{k_5}{K_3k_7}\right)}. \tag{S6}$$

In the case of two states, the combination of expression (24) for K_M and the expression for the current (S4) yields a new expression for the current equal to

$$J = \frac{k_{cat}[S]}{K_M\frac{K+K_1}{K-1}+[S]} \quad \text{(S7)}$$

which is analogous to the Michaelis-Menten equation but still differs from it because we work with the reversible product-release step and forward k_{cat} and K_M.

It can be seen from this expression that if [S] is small compared to the first term in the denominator, then [S] can be ignored in the denominator. With K_1 and K_2 constant, the flux becomes $\boldsymbol{J\sim[S]k_{cat}/K_M}$. On the other hand, if [S] is large compared to the first term in the denominator, the first term in the denominator can be ignored, and the flux becomes $\boldsymbol{J=k_{cat}}$. For the first case, the flux slope is a linear function of k_{cat}/K_M that varies with [S]. k_{cat} gives the flux at a very high substrate, while k_{cat}/K_M determines the flux at a very low substrate concentration.

References

1. Juretić, D.; Bonačić Lošić, Ž.; Domagoj Kuić, D.; Juraj Simunić, J.; Dobovišek, A. The maximum entropy production requirement for proton transfers enhances catalytic efficiency for β-lactamases. *Biophys. Chem.* **2019**, *244*, 11-21. doi: 10.1016/j.bpc.2018.10.004.
2. Juretić, D.; Simunić, J.; Bonačić Lošić, Ž. Maximum entropy production theorem for transitions between enzyme functional states and its application. *Entropy* **2019**, *21*, 743. doi: 10.3390/e21080743.
